# Supplementary material for: Genome-Wide Identification and Characterization of Lectin Receptor-Like Kinase Gene Family in Cucumber and Expression Profiling Analysis under Different Treatments
Source: Genes (Basel). 2020 Sep 2;11(9):1032. doi: 10.3390/genes11091032 (PMC7564967; doi:10.3390/genes11091032)
Supplement: Supplementary file 1 [file genes-11-01032-s001.zip › genes-891962-supplementary/Additional file1 Figure S1.pdf]

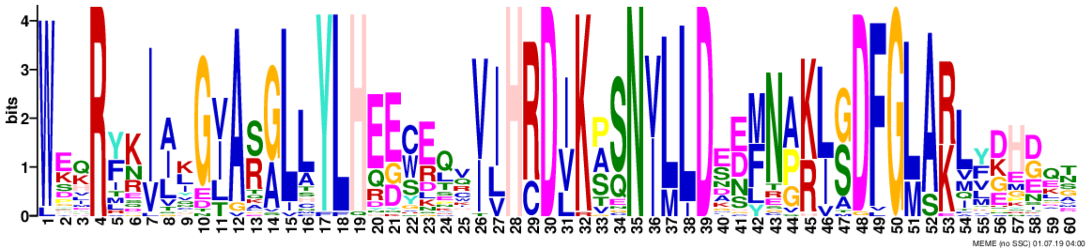

Motif 1：

Length: 60

Annotation: Catalytic domain of the Serine/Threonine kinases

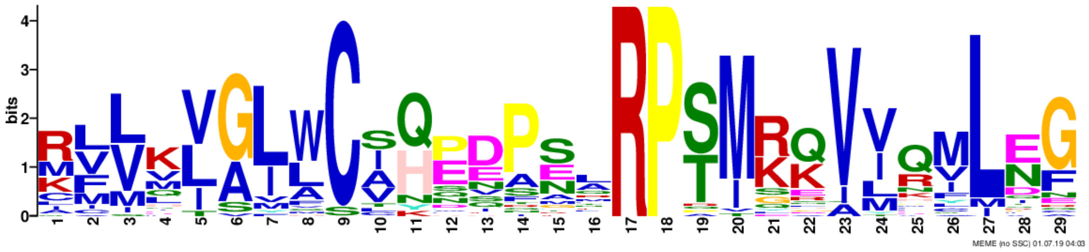

Motif 2：

Length: 29

Annotation: Catalytic domain of the Serine/Threonine kinases

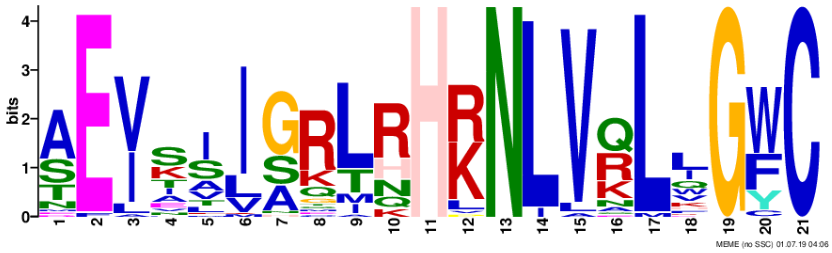

Motif 3：

Length: 21

Annotation: Catalytic domain of the Serine/Threonine kinases

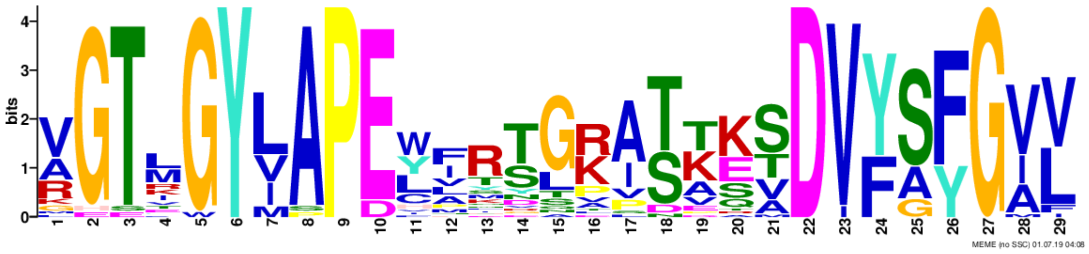

Motif 4：

Length: 29

Annotation: Catalytic domain of the Serine/Threonine kinases

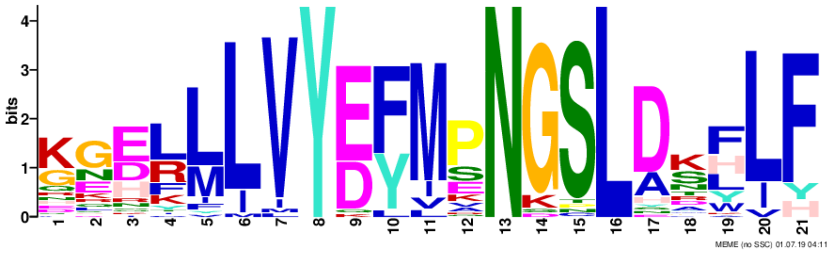

Motif 5：

Length: 21

Annotation: Catalytic domain of the Serine/Threonine kinases

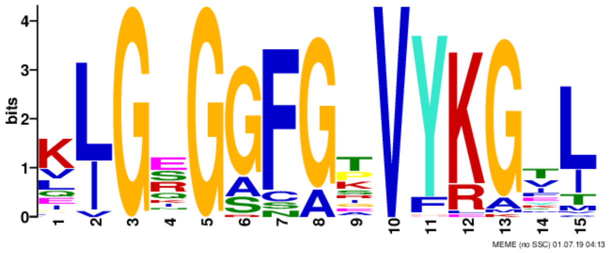

Motif 6：

Length: 15

Annotation: Serine/threonine-protein kinase SNT7

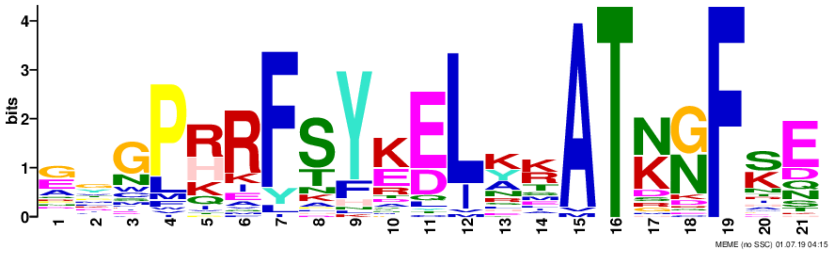

Motif 7：

Length: 21

Annotation: None

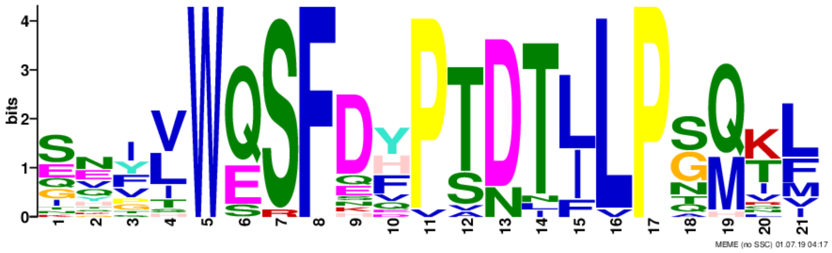

Motif 8：

Length: 21

Annotation: D-mannose binding lectin

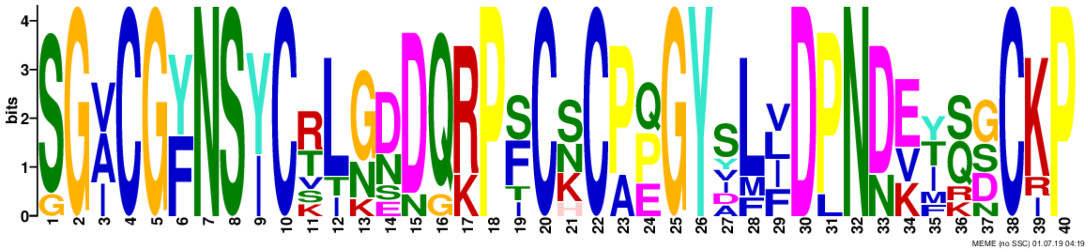

Motif9：

Length: 40

Annotation: None

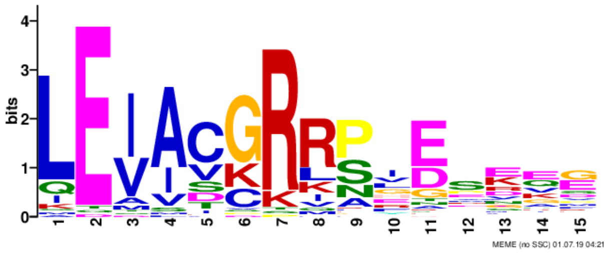

Motif10：

Length: 15

Annotation: None
