# Supplementary material for: Genome-Wide Identification and Characterization of Lectin Receptor-Like Kinase Gene Family in Cucumber and Expression Profiling Analysis under Different Treatments
Source: Genes (Basel). 2020 Sep 2;11(9):1032. doi: 10.3390/genes11091032 (PMC7564967; doi:10.3390/genes11091032)
Supplement: Supplementary file 1 [file genes-11-01032-s001.zip › genes-891962-supplementary/Additional file3 Table S1.pdf]

Table S Primers for qRT-PCR

Gene	F	R
Csa1G004050	'5-GATTTCATTCTCATTAGCGACG-3'	'5-GAAGCCTGGACTAGTAGCG-3'
Csa1G056960	'5-TAACGAGTCAGGAATTGGGT-3'	'5-CTCTTGATATGTTACCAGAGCTG-3'
Csa1G071150	'5-GCTTATAGTTGGCCTGAGTGT-3'	'5-GTGGCAGTCTCAATCTTCGT-3'
Csa1G071160	'5-GGAAGCAGCTTATAGTTGGG-3'	'5-ATAATTCGTGGCAGTCTCAATC-3'
Csa1G071170	'5-GCCTCACATGGTTTAATAAGTTG-3	'5-GCTTATCAGTGACGCAACAG-3'
Csa1G071270	'5-CAATAGTGCTACCAACGACC-3'	'5-CACTTTCGTCCCCAATCATG-3'
Csa1G073890	'5-GGTACGCTCAACAGACTTAG-3'	'5-GAACTCGTGAACCCCATATAC-3'
Csa1G605730	'5-GTGAAGATGGTGAGAACAATTC-3'	'5-TCTCTATATACAACTGCCACAC-3'
Csa1G605740	'5-CATGTGAAGGTGATGAGAACAAT-3	'5-CTATTATCTCTATATACAGCCACC-3'
Csa1G605750	'5-GGATCAGTTTGGGAGAGCTT-3'	'5-GGGTAAATTTATGGTGTTTAGAAC-3'
Csa2G439150	'5-AGTGATCCTGCATATTCCTGTC-3'	'5-GCTGAGTTTGGAGGGATATG-3'
Csa2G439210	'5-GGGATTCTGAAACAGCAAAACTC-3'	'5-CCAAGAAACCCACCAGCTG-3'
Csa3G048440	'5-CTGACTCTCCTTGGCGATTC-3'	'5-GTTGGTGATGGAGAAAGAGAATC-3'
Csa3G099580	'5-CGGTTTTGGTTTGGCTATCAT-3'	'5-CCCAAAGCCACCTTCTCCT-3'
Csa3G115060	'5-GCTTCTTACGCTCAACCCG-3'	'5-GCCAAATCCGAGCTAAATAATCC-3'
Csa3G115090	'5-CCATGAGGGTGATGATAGTGA-3'	'5-TCGTGGAAGTATAGTAGTGCTG-3'
Csa3G730910	'5-ATGATGGCCGTAGTAAGGTAGT-3'	'5-AACTCCAACCTGAAATGTAGTGG-3'
Csa3G730920	'5-TTCAGGTGCAGGAAGTGGG-3'	'5-GAAATGTCATTGATGTCGATCCC-3'
Csa3G733860	'5-GGGAAGATGGAGGATGCGA-3'	'5-TAGATATGCGATGAGATTCTGG-3'
Csa3G733880	'5-CACGGATTATGGGTTAGGTCC-3'	'5-GCAAGAACAAGTCGGTAATGG-3'
Csa3G734030	'5-CTACATTTTCGACGACCCGG-3'	'5-GCTTTCCATTCATCTCGCTGT-3'
Csa3G734040	'5-GGATAGTTACATATTCGGCAAAC-3'	'5-TTCATAGAGTTTGGCGAGGC-3'
Csa3G734050	'5-GTGCAGTACTTGGAAGGAGA-3'	'5-CAAGTGAAGAAGTAGAATATGCAAT-3'
Csa3G736960	'5-GTCATTGTCGATTTCACTGCCA-3'	'5-TGGGTTTAGCGTAAAGGACTC-3'
Csa4G005510	'5-TGAGTATATGAGCAATGGATCC-3'	'5-GCAGTGGATTATCTTTGATTCGC-3'
Csa4G288620	'5-TTGGGGCGTGGCTCTTTTG-3'	'5-CACCAACATTCTGTGCTCTC-3'
Csa4G289620	'5-GGATTACGCCAATTTCAACCCA-3'	'5-AATAGAGCTTCCTACTAGAACTG-3'
Csa4G289630	'5-GGGTTATTGATGATTGTATGGAC-3'	'5-AAATCACTTCAATTCTCTCATACC-3'
Csa4G289640	'5-GTCTGGCTCCATTTATGTCTC-3'	'5-GAAATCTGAAACACTCTTCCAAG-3'
Csa4G289650	'5-CTCACTTTATTGATCGGCTGTC-3'	'5-CCAACTTTTTAACTGCCACCAAG-3'
Csa4G290150	'5-CTCTTTAAGCCTTCAAGACCAA-3'	'5-ACAAACCTCTCCACTCTCTTC-3'
Csa4G296230	'5-GATTGGGGGATTTTGGTTTGG-3'	'5-CTTCTTCCACAGGCCACTTC-3'
Csa4G296250	'5-CTACCCTGGTTTCTATGGATC-3'	'5-GACAAAACAAGTGGAGAAAGAGG-3'
Csa5G550210	'5-GTGTGGTAGCCGTCAAGTG-3'	'5-TATCTCCTTCATCTTCTCCTCCA-3'
Csa5G550230	'5-CAGGTCAAATCAACAGAGATAATC-3'	'5-CAACTCTACATGTGCTTGCC-3'
Csa5G648630	'5-GCCCTCATAGATTCAACTTCAAG-3'	'5-CCTTCATTCCTTGGCTTGAG-3'
Csa6G052130	'5-ATGGCTCTCTTGATGGATTGAT-3'	'5-ACCCTACTAACTTGGTCCATATC-3'
Csa6G338050	'5-ACAGCCATATACCCCTTCCC-3'	'5-GCCATGAGATCGAAAATCCAC-3'
Csa6G516770	'5-CGTGTGTTGCATATTCCGATG-3'	'5-ACATATCGCCACTATCACACC-3'
Csa7G029930	'5-CAAGCAATTCTCCAGGGATAG-3'	'5-TCTGCTCATCAACATCATATAGG-3'
Csa7G045520	'5-CTGCTTGTTGGATCTGGTCT-3'	'5-GCTAAGTGGATAAGGAATGAGG-3'
Csa7G048050	'5-ACTGGAATCAATGGAACTAGCTC-3'	'5-GAGAATTCAAATCCACACCCACA-3'

Csa7G067400	'5-TGCTGCGACTGGAACTCTAT-3'	'5-CCACAGACTAAGAACACTACAC-3'
Csa7G067410	'5-GGGATGAAGGCTCTGGAAAC-3'	'5-TCGGGATCATAGCCAATACC-3'
Csa7G067430	'5-CGTCGGCGTTGATGTTGAC-3'	'5-GTCAAATTATGGTAGCTTGAGTTG-3'
Csa7G446780	'5-GGGTGCAAATAACTGTGGCTC-3'	'5-ATATCATCCCCATCCCCCG-3'
CsaACTIN	'5-TCGTGCTGGATTCTGGTG-3'	'5-GGCAGTGGTGGTGAACAT-3'
