# Supplementary material for: Genome-Wide Identification and Characterization of Lectin Receptor-Like Kinase Gene Family in Cucumber and Expression Profiling Analysis under Different Treatments
Source: Genes (Basel). 2020 Sep 2;11(9):1032. doi: 10.3390/genes11091032 (PMC7564967; doi:10.3390/genes11091032)
Supplement: Supplementary file 1 [file genes-11-01032-s001.zip › genes-891962-supplementary/Additional file4 Table S2.pdf]

C-Type

gene: Csa1G056960
ATGGCTCCGACAGAGGTGAAAACGCTATTTCTTTCTCTTTTCGTGTTAATTTGGTTGTCC
AGAATGGGCGTCTCTGATACTATGTTTAACGAGTCAGGAATTGGGTATTCGGTTGTTTC
CAGAAAGGAAATTTCAAAAGGACGGTGCCCACATGGTTGGATTATTAGCCCAAGCAAG
ACTAAGTGCTTTGGTTTCATGTCCAGCCCTAAATCATGGAATGATTCAGAGACCCAATG
CAATAGTTTTGGTGGGAACTTAGCAGCTCTGGTAACATATCAAGAGTTTAGCTATGCAC
AAAATCTGTGTAATGGAACTCTTGGTGGCTGTTGGGTTGGTGGCAGAGCGTTCAACTC
TTTGAATGATTTCGTTTGGAAGTGGTCAGATAATGTTTCAAAATGGAACGATTCCATTTT
TCCCAGCGCAACTTTGCAATCGAACTGCAAAAATGCCTCTTGCCTCCGGAATGATTCG
GTTGAAACATGTACATTAATATTTGGTGGACCAGCAACACCATTCCTTAGGGACGAGAA
ATGCAACAGTTCTCACCCTTTTATATGCATGATAAACCTAGATGATAGATGCCACCGAAT
GCACTGTCACAAGGAGTATCTAGTTATTCTAGCTGTTGTGAGTGGGTTGATATTTTGCAC
AACTTTAGCTGTGGTGATTTGGCTTCTAGCCCACAAGAGGAGCAAAAAGCGGAGAAG
GTCGCGGAAGCCATCCAACCCTGCAGCTTCTGCACTGGTACCGCCTTTGTGGAGGGTG
TTTACCAAAGAAGAGCTAAGATCAATGACAAAAAACTTCAGTGAGGGAAACCGTCTT
CTCGGAGATGCAAAAACAGGCGGTACATACAGTGGACTTCTACCTGATGGTTCAAGAG
TTGCAATTAAAAGGTTGAAGAAGTCCAGCTTTCAGAGGAAAAAGGAGTTTCATTCAGA
AATTGCAAGAGTTGCTAGGCTTCGTCACCCAAATTTAGTTGCCCTGAAAGGATGCTGCT
ATGACCATGGTGACCGCTACATTGTTTATGAATTCATTGTTAATGGGCCCTTAGATAGAT
GGCTTCATCATGTACCAAGAGGTGGTCGAAGCTTAGATTGGACTATGAGAATGAAAATT
GCCACGACTCTTGCCCAAGGGATTGCGTTCCTTCACGACAAGGTCAAACCACACGTGG
TACATCGTGATATCCGTGCGAGTAATGTGCTTCTCGATGAAGAATTTGGAGCACATCTG
ATGGGAGTTGGCCTATCGAAATTGGTTGCATATGAAGTGATGCATGAGAGGACAGTGAT
GGCAGGAGGAACCTATGGATACCTTGCTCCTGAATTTGTCTACAGAAACGAGCTAACA
ACAAAGAGTGATGTCTACAGTTTTGGGGTCCTATTGCTTGAAATTGTAACCGGACGAA
GGCCTGCACAGGCAGTTGATTCAGTTGGTTGGCAGAGCATTTTTGAATGGGCAACCCC
GCTCGTGCAGGCTCACCGTTACCTAGATCTATTGGATCCTCATATAACAGCCACTTCAAC
TTCTGAAATCCCAGAGGCTGGCATTGTTCAAAAGGTTGTGGACCTTGTTTATGCTTGCA
CACAGCATGTCCCGTCCATGCGACCTAGAATGTCACACGTCGTTCATCAACTGCAACAA
TTGGCTCCCTCTCCTCTCACTACGAAGTAA

G-Type

gene:Csa5G550210
ATGTTTTTCGACGCCGCCCCTCTCTCCATCCTCCTCCTCTTACCTCTGGCCTCCGCCCAA
CCCACAACAAACCCCCGCAAATTCTCCTCCTTCTCAATTTCCCAATCTCCATGGCGGCC
CACCCAGAACCTCACTCTCCTTTCCCCCAACTCCCTTTTCGCCGCCGGCTTCCACCCAC
TACCCAACAACTCCAATCTCTTCATCTTCTCCGTTTGGTACTTCAACATCTCAACCGAC
AACGTCGTCTGGTCCGCCAACCGTCTTCATCCGGTCAACCGTTCAGCAGCTCTGGTCA
TCACAGCCACCGGCCAGCTCCGTCTCAACGACGCCTCCGGTCGCAACCTCTGGCCTTC
CAACAATGTCTCAGCCCATTCAAACTCGACCCAATTAATCCTTCGCGACGATGGTGACT
TGATTTACGGCACATGGGAAAGCTTCCAATTCCCCACCAATACTTTCTTGCCTAATCATA
CATTTAATGGAACCTCAATCGTCTCCAACAACGGCAAATATTCGTTTGTAAACTCTGCT
AATTTGACGTTTGGTACAGAGACTTATTGGTCCTCTGGAAATCCATTCCAGAATTTTCA
AATTGATGGTCAAATTATCATAAACAATCAAATCCCAGTTATCCCTTCTGATTTTAACTC
GACCCGGTTCCGGAAATTGGTTCTTGATGATGATGGGAACCTCAGGATTTTTAGTTTCA
ACCCCAATTGGCCTCGGTGGGATGTAGTTTGGCAAGCACATGTAGAGTTGTGTCAAATT
TTAGATACTTGTGGCCCAAATTCTGTCTGTATGAGTAGTGGCAGTTACAATTCCACTTAT
TGTGTCTGCGCACCCGGATTCAGCCCCAATCCTCGAGGAGGAGCGCGGCAGGGATGCC
ACCGGAAACTCAACGTATCAAACAAACCCAAGTTTCTTCAACTGGATTTTGTGAATTTC
AGAGGCGGGGTTAAACAAATCTCTTTGCAAACTCCAAATATTTCAGTCTGTCAAGCGG
ATTGCTTGAAGAATTCGAGCTGCGTGGGCTATACATTCAGCTTCGACGGCAACGGCAA
CGCCCACGCCCAGTGTGTGCTTCAGCTAGACATTTTGTCGAACGGGTTGTGGTCTCCT
GGGATGAAGGCAGCTGCCTTTGTGAAGGTCGACAATTCGGAAACAGATCGCTCAAATT
TCACCGGAATGATGTACAAACTCCAAACTACATGTCCAGTTCGCATAACCCTCCGGCCC
CCACCGGTAAATAAAGACAACACCACCAGAAACATATTGATAATCAGTACCATATTCGT
TGCCGAACTAATTACCGGTGCAGTTTTCTTCTGGGCATTCTTGAAGAGATTTGTAAAAT
ACAGAGACATGGCTCGCACTCTTGGTCTCGAATCACTTCCCGCCGGCGGCCCAAAGCG
GTTCAATTATGCCGAGCTAAAGACGGCCACAAACGACTTCTCGACCTGCATCGGAAGA
GGGGGATTCGGCGAAGTCTTCAAGGGGGAGCTGCCAGACAAACGTGTGGTAGCCGTC
AAGTGCTTGAAAAACGTCGCGGGAGGTGACCGTGACTTTTGGGCGGAGGTCACCATC
ATCGCGAGGATGCACCATCTTAACTTGCTTCGATTATGGGGATTCTGCGCAGAAAAGGG
ATGGGCATTCGAGAAGGCGTTCGTGGAGGAGAAGATGAAGGAGATATTAGACGGTCG
GATAAGGGAAGAATACGAGAGGGGGGGTAATGTATGTATTGTTAATCGAATGGTGGAA
ACGGCGATGTGGTGTCTTCAAAACCAACCGGAGAAGAGACCGTCGATGGGGAAAGTG
GTGAAGATGTTGGAAGGGAAGCTTGAAATTCCTCCTCCAGAAAAGCCTTCTATTTACTT
CTTGTCTCAATGA
>gene:Csa5G550230
ATGTCTCTCGCCGTCGCTTTCTCCGCCCTCTTCCTTCTCTTCTTCCCATCTCCGGCCGCA
GCCCAGTCTCCGAAACCCACCAATTTCTCTGCCTTCTCTATCTCCCAATCTCCATGGCG
ACCCTCCCACAATCTCCTTCTCCTCTCCCCCAACTCCCTTTTCGCCGCCGGCTTCCGCC
CACTACCCAACAACTCCAATCTCTTCATCTTCTCCGTTTGGTACTTCAACATCTCAACA
GACAATATCGTCTGGTCCGCCAACCGTCTTCATCCCGTCACCCGTTCAGCGGCTCTGGT
CATCACAGCCACCGGCCAGCTCCGTCTCAACGACGCCTCCGGTCGCAATCTCTGGCCT
TCCAACAATGTCTCTGCCAATTCAAATTCGACCCGATTAATCCTTCGCGACGATGGTGA

TTTGATTTACGGAACATGGGAAAGCTTCCAATTCCCCACCAATACTATCTTGCCTAATCA
GACATTGAATGGAACCACCATCATCTCTAACAACGGCAAATATTCGTTTGTGAATTCTG
TTAATTTGACGTTTGGAACAGAGAGGTATTGGTGGACTGATAACCCTTTCAAGAATTTC
GAAAATACAGGTCAAATCAACAGAGATAATCAAAACCCAATTTACCCTACTGATTTTAA
TTCGACCCGG
CTCCGGAAATTGGTTGTTGACGATGATGGGAACCTCAAGATTTTGAGCTTCAACCCCA
ATTCTCCGAGGTGGGATATGGTTTGGCAAGCACATGTAGAGTTGTGTCAAATCTTTCGT
ACTTGCGGCCCAAATTCTGTCTGTATGAGTAGTGGCAGTTACAATTCCACTTACTGTGT
CTGTGCTCCTGGATTCAGCCCCGATCCTCGCGGTGGAGCACGTCAAGGATGCAACCGG
AAACTCAACGTATCGAACAAATCCAAATTTCTACAACTGGATTTTGTGAATTTTAGAGG
TGGGGCTAATCAAATCTTCATGGAAACCCCAAATATTTCAGTCTGTCAAGCGAATTGTT
TGAAGAAT
TCGAGCTGTGTGGGCTATACATTCAGTTTCGAGGGCAACGACCAGTGTGTGCTTCAGCT
AGACATTTTGTCGAACGGGTTCTGGTCGCCTGGGATGAAGACGGCTGCCTTTGTGAAG
GTCGATAATTCGGAAACAGATCAGTCAAATTTCACCGGAATGATGTACAAACTCCAAA
CCACATGTCCAGTTCACATCAGTCTCCGGCCACCACCGGATAATAAAGACAATACCACC
AGGAACATATGGATAATCGTCACCATATTCATAGCCGAACTAATCTCCGGGGCGGTTTTC
TTCTGTGCATTCTTGAAGAGATTTATCAAATACAGAGACATGGCTCGCACGCTTGGTTT
CGAATCACTACCCGCCGGTGGACCGAAGCGGTTCAGCTACGACGAGCTGAAGATTGCC
ACCAACGACTTCTCAAATCCCGTTGGAAAAGGTGGATTCGGCGAAGTCTTCAAAGGG
GAATTGCCGGACAAACGTGTTATCGCCGTGAAATGCCTGAAAAACGTCTCTGGCGGCG
ATGGGGACTTTTGGGCGGAAGTCACCGTTATCGCCAGAATGCACCATCTCAACTTGCTC
AGATTGTGGGGATTCTGTGCCGAAAAGGGTCAAAGAATGTTAGTCTACGAGTACATCC
CCAATGGATCTCTCGACAAATTCCTCTTCGTCAAATCTTCGTTCTCTGATTCGATAGAGA
TAGATGGAGAAAACCCATTGCTGGATTGGGGAATCCGTTATAGAATCGCCATTGGAGTT
GCAAGAGCAATCGCGTACTTGCACGAGGAATGCCTGGAATGGGTATTGCATCGAGACA
TAAAGCCCGAAAATATCCTTCTAGACAACGATTTCTGCCCGAAACTAGCGGATTTTGGG
TTGTCGAAACTAAAGGAAAACGACGGAACAGCGGTGAGTATGTCTCGGATCAGAGGC
ACCCCCGGTTATGTGGCACCGGAGCTAGTAAAATTGGGTTCAAATTCAATCACGCCGA
AAGCAGATGTGTATAGTTTCGGAATGGTGCTGCTGGAGATTATCAGTGGGACCAGGAAT
TTCGATACAAAAGAAGGATCGACAGTTGAGAGCGCGTTTTGGTACTTTCCGAGCTGGG
CATTCGAAAAAGCGTTTGTGGAAGAGAAGATTGAAGAAGTTTTAGACAGCCGGATCA
GGAATGAGTACGATAGTGGAGGCCATTTTGCGATTGTTAACCGTATGGTGCAAACGGC
AATGTGGTGCCTTCAGAGCCAGCCGGAGATGAGACCATCGATGGGGAAAGTGGTGAA
GATGTTGGAAGGGAAGTTGGAGATTCCAAACCCAGAAAAGCCCTCCATTTACTTTCTC
TCAGAAGGACAGGAAGGTCCTAAACATCAAATAGCCATGGTGGTCGATTCTGTAGATT
CCATGGATTCTGACTTTCCTCCAGCTGAATACAGCTCAACTAGTCTAAGTTTCGGGTAA
>gene:Csa1G071270
ATGAATTCCGCCTTTCCTCTTCTTCTTCTTCTTTCATTAGTTACGACATTCTTCTCTTCAA
AGATCTTCGCCTATGGCAGTACTGATACAATCACATCAACAAATTTCATCAAACACCCT
TCAACCATAATTTCCAATGCCGATTCCTTCCAATTGGGCTGGTTCTCACCTCTTAATTCC
ACAGCACAATACGTCGGAATTTGGTACCATCAAATTTCCATACAAACTCTAGTATGGGT
TGCTAACAAAGACACCCCTCTCAACAATACTTCCGGAATTTTCACTATCTCCAACGATG
GAAATCTTGTGGTCTTAGACGAATACAACACCACCATTTGGTCTTCAAATATTACTTCA

CCCACAGCCAATACAACCGCTCGGATTCTGGATTCAGGCAACCTTGTCTTGGAAGATC
CTGTTTCAGGGGTTTTATTTGGGAGAGCTTCGAACACCCTTCCAATTTACTCTTGCCT
GCCATGAAACTTGTCACAAATAAGAGAACTCAACAGAAGCTCCAATATACCTCATGGA
AAACCCCTTCTGATCCATCTAAAGAAGGGATTTTGGAGCAACAGTTCTGGAATCAATC
GAAGGGAAATTGGGAACAGAGTTGGTCGGCTTTCAGCACAGAATGTGATTATTACGGT
GTTTGTGGGGCGTTTGGGGTATGTAATGCAAAAGCAACCCCTGTTTGCAGCTGTTTAAC
TGGGTTTAAACCGAAGGATGAAGATGAATGGAAACGAGGAAATTGGAGTAATGGGTG
TGAGAGAATTACGCCATTGCAGTGTGAGAGCAGCGCTAGAAACAACAGTAGAGTTGA
GGAAGATGGATTTCTGCATTTGGAAACGGTTAAAGTTCCATTTTTGGTAGAGTGGTCGA
ATTCGTCTAGTTCAGGGAGTGATTGCAAACAAGAGTGCTTTGAGAATTGCTTGTGTAAT
GCTTATGCATATGAAAATGGCATTGGTTGTATGCTATGGAAAAAAGAGTTAGTTGATGTA
CAGAAATTTGAAAACCTTGGAGCCAATCTTTATCTTCGACTGGCCAATGCAGAATTGCA
GAAAATTAATGATGTAAAAAGATCAGAGAATAAAGGAACTGTTATAGCAATAGTGCTAC
CAACGACCCTAGTGATCTTCATCATTATTGTCATTTACTTCTGTTGGAGATGGAAGGCTA
ACAAAAATGAATATATCAAAAACGGGAAGAGATTGAAGTTGAGAAAGGACGACATGA
TTGGGGACGAAAGTGAATTGAAAGAACTACCTCTTTATGATTTTGAGAAGCTGGCAAT
CGCAACAGACAGTTTTGATTTAAGTAAGAAACTTGGACAAGGTGGTTTTGGTCCTGTG
TATAAGGGAACATTGTTAGATGGACAAGAAATAGCAATAAAGAGGCTTTCAAGAGCTT
CTAATCAAGGGTACGAGGAATTTATAAACGAAGTCATTGTGATTTCAAAACTACAACAT
AGAAATCTTGTACAGCTTCTCGGTTGTTGCATTGAAGGAGAAGAGAAGATGCTAATCT
ACGAGTACATGCCCAACTCAAGTCTGGATGCTTTTATCTTTGGTAAGATCTATCGATGTC
TTTCCTCTGAATTTCAAGATTTCTCTATATTCTGCTTTAGAATAACTAATCACACTAACCA
CAATGCAAACATTTTGCATGTAGGTTCTGCTAAACAAAAACTCTTGGATTGGAGAAAA
AGATTTAACATCATCAATGGAATTGCTCGAGGTCTCCTTTACCTTCATCGAGATTCAAGA
TTGAGAATTATTCATAGAGATTTAAAGGCAAGTAATATTTTACTAGACAAAGATATGAAT
CCTAAAATTTCAGACTTTGGTATGGCAAGAATTTTTGGCAGCAACGAAGTGGAAGCCA
ATACTATAAGGGTCGTTGGAACTTATAGAAATTATCCAACTAACAATGATTAA
>gene:Csa1G605750
ATGATTCCACCAACTGTTTCTCTTCTTCTCTTCCTAACTTCCCTCCTTCTATATGCTCAAA
GCAACGCAACTCAAATCCCCACTGGCAGCTCTTTAATTGCCGGCACCTCTTCCCTTCAC
CCATGGCTTTCTCCTTCAAACCATTTCGCGTTTGGATTCCAAAACCTCGATAACGATAA
CCGTTATCTTCTCGCAATTTGGTTTTACAAAGTACCTGAAAACAACATCGTTTGGTTTG
CAAAATCAGACGACGACGACAACAACAACAACCCTGTTTTCGCTCCAAAAGGGTCCA
AGATTCAGCTCACTGCTTCAACCGGATTGGTTCTTCGAAACCCTAACGGTGAAGAAAT
TTGGAAATCGAAGCCTATTACATCCTCTATTTCATTCGCTACTTTGAACGATACGGGTAA
TTTCATGCTTGTGGATTCAATCAATGGATCAGTTTGGGAGAGCTTCTCATATCCAACAGA
TACATTGCTTCCTTCGCAGAAATTGGAAGTAGGAGGTGTTCTTTCTTCTCGTAAATCGC
TAGGTAATTTCTCCCTTGGAAAATTTCAGTTCCGTTTGCTTGAAGATGGAAATGCTGTT
CTAAACACCATAAATTTACCCTATGGTTATCATTATGATGCATATTATATTAGCAATACTTT
CGATCCTGCTAGTACACAGAATTCTGGTAGTGAAGTGATTTTTGATGAAGTTGGATTTT
TGTATGTGCTGAAAAGAAATGGAGTACAAGTTAACATTACACAGTTTAGTGTTGGTAAT
CCTGTTGAGGCTTTTTATTACAAAGCCACCATGAATTTTGATGGAGTTTTGACTGTAAG
TTCTTATCCTAAGAACACTAATGGAGTAGTTGCTAATGGAAGTTGGAAAGATTTGTTTA
GAATACCTGATAATATCTGTCTTTCTAATGAGAATCCCATTACAAGACTTGGTTCTGGAA

TCTGTGGATTTAATAGTATTTGCTCATTGAAATCTAATGGAAGGCCGAGTTGTAATTGTG
CACAAGGATATTCTTTCGTTGATCCGAACAATGAATTCAGTAACTGTAAACCATTTATTG
CACAGGGTTGTGAAGACGAAGATGACAAATTCAACCAGAATCTGTATGAAATGGTGGA
TCTTCAATACACTAATTGGCCAATGTATGATTATGAGCGGTTTCCCACCATGAATGAACA
AACTTGCAAAAGTTCTTGCTTGGAAGATTGCTTTTGTGTATTAGCAGTGTTTGGAGGTA
GAGATTGTTGGAAGAAAAGGTTACCACTCTCTAATGGAAGACAAGATGCAAGTATTAC
ATCTATTTCTTTCCTTAAATTAAGAAAAGATAATGTCTCTCTTGAGAGTTTCCCAAATGG
TGGTGGAGCACAAAAGAAACAAACCACAATAATCCTTGTCATCACTGTACTCCTTGGT
AGCTCTGTTCTTATGATCATATTATTGTGTTTCTTTGTCTTGAAAAGAGAGATATTAGGTA
AAACTTGCACAAAGAATTTCTCTTTGGAATGCAATCCGATCCGTTTTGCATATATGGATA
TTTACAAAGCAACAAATGGTTTCAAGGAAGAACTAGGCAGGGGATCTTGTGGGATTGT
TTACAAAGGAACAACAGAATTAGGTGATATTGCTGTCAAGAAATTGGACAGAATGTTT
GAAGCAGAAAGAGAGAAGGAGTTCAGAACTGAAGTGAATGCAATTGGCCAAACACA
CCACAAAAATTTGGTTCGTCTACTCGGATACTGCGATGAGGGTAACAACCGAATGCTC
GTTTACCAATTCATGAGCAATGGCTCATTATCTACTTTCCTCTTCAATAATGATCCCAAA
CCAAGTTGGAAGCTTAGAACTCAAATAGCCTACGAAATCGCCAGAGGACTCTTGTATC
TGCACGAAGAATGTGGCACACACATCATTCACTGCGATATAAAGCCTCAAAACATACTT
CTCGACGACAATTACAACGCCAAGATCTCTGATTTCGGGTTGGCAAAATTGTTAAAGAT
GGATCAAAGTAGAACCCAAACTGGCATAAGAGGAACAAAAGGGTATGTGGCTCCAGA
TTGGTTCAGATCTTCGCCGATAAATGCCAAGGTCGACGTGTACAGCTATGGAGTGTTGT
TGCTAGAGATCATATGTTGTAGAAGGAATGTGGAGATGGAAGTTGGTGATGGTGCTCA
AGGAGAGAGAGGGGTTTTGAGTGATTGGGCTTATGATTGCTATGAACAAGGGAGGTTG
GATATTTTAATTGAAGGAGATACGGAGGCCATAGATGACATTGTTAGAGTTGAAAGATT
TGTAAAAGTAGCAATTTGGTGCATTCAAGAAGAGCCATCCAGAAGACCAACCATGGAG
AATGTTATGTTAATGCTTGCCGGGAATTTAGAAGTTTCTCTTCCTCCATGTCCTTACCAC
TCATTTAGCTCCATTGTTTAA
>gene:Csa1G071170
ATGGGAAAACTGATATGGAGATTCAGTGTTTCGATTTTTCTGTTTTTCTGGATGACCATG
GCTTTTTTATCAAGAAAATCATTGGCAATTGATAGCATAAAAGCAGGGGAATCCATCAA
TGGAAATACCCAGATTTTAGTTTCAGCTCAACAGAAGTTCGTGCTGGGAATTTTCAATC
CTAAAGACTCCAAATTTGGTTACTTGGGAATATGGTACAAGAACATCCCACAAACAGTT
GTGTGGGTAGCAAACAGAGACAGCCCACTTGTAGATTCCTCTGCCAGATTAACACTCA
AAGGACAAAGCCTGGTTCTTGAGAATGAAAGCGATGGAATTCTATGGTCTCCCACTTC
TTCAAAATTCCTGAAAGATCCAATAGCTCAACTACTTGATAATGGTAATTTGGTTATAAG
AGAATCTGGGTCTGAACATTATGTGTGGCAGAGTTTTGATTACCCTTCTGATAATCTGTT
ACCTGGCATGAAAGTGGGTTGGGACTTGAAAACCAGGATGAACTGGAAGTTAACGTC
ATGGAAAAGCTCGAATGATCCGTCTTCAGGGGATTTCACTTATGGTATGGACCCTGCTG
GGCTTCCCCAGCTTGAAACTCGCAGAGGAAATGTAACAACATACAGGGGTGGCCCATG
GTTCGGTAGGAGGTTTAGTGGCACTACCCCTTTTAGAGATACAGCTATTCATTCTCCAC
GGTTCAATTATAGTGCGGAAGGAGCATTCTATTCATACGAGTCTGCAAAAGATCTTACT
GTAAGATATGCACTGAGCGCAGAAGGGAAATTCGAACAATTTTATTGGATGGATGATGT
AAATGATTGGTACCTTCTGTATGAATTACCGGGAGATGCCTGCGATTACTACGGACTCT
GCGGAAATTTTGGTGTTTGTACATTTTCTACCATACCGCGCTGCGATTGCATTCATGGGT
ATCAACCAAAATCGCCAGACGATTGGAATAAGCGTCGCTGGATAGGAGGCTGCGTTAT

AAGGGACAATCAAACCTGCAAAAATGGAGAGGGGTTTAAAAGAATCAGCAATGTAAA
ATTGCCAGATTCTTCAGGGGATTTGGTGAATGTTAATATGAGCATTCATGACTGCAAAG
CGGCGTGCTTGAGTAATTGCTCTTGCTTGGCCTATGGAATGATGGAGCTTTCCACAGGA
GGATGTGGTTGCCTCACATGGTTTAATAAGTTGGTGGATATTAGAATCCTTCCTGATAAT
GGGCAGGATATCTATGTGAGGTTGGCTGCTTCAGAATTAGAATCGGATAAAAGGAAGC
TTACAGTTGTGCTTTGTCTGTCTGTTGCGTCACTGATAAGCTTCTTGATTTTTGTTGCTT
GCTTTATCTTTTGGCGTAGAAGGACGATTAAGGGTAATGAGGTTCAGTCTCATGAAAAC
GAAGCTGAGATGCCACTTTATGATTTTTCCATGCTCGTAAATGCCACAAATGATTTTTCT
CTCTCAAATAAGATTGGGGAAGGTGGTTTCGGACCCGTCTACAAAGGAGTGCTTCCAT
GTGGACAAGAAATTGCAGTAAAAAGACAGGCAGAGGGTTCAAGCCAAGGGCAAACG
GAGCTAAGAAACGAGGTTCTATTGATCTCCAAACTCCAACATCGTAATCTTGTCAAGCT
GCTTGGTTTTTGCATTCATCAACAAGAAACATTGCTGGTCTATGAATATATGCCCAACAA
AAGTCTTGACTATTTTCTCTTTGGTTGGTGCTTAACTTGA
>gene:Csa1G605730
ATGGCTTGTATGATTTCTCATATTTTTCTCTTGCTGCCTTCAGTGGTTTATGCTCAAAGTA
ATAGCATGCTGAATGTTGGTGGCTCTCTTATTGCTGGTGATGCTTCTGCTTCTCCATGGA
TTTCTCCAGCCGATCACTTTGCATTTGGGTTTCGGGAAGTCGATGACGGGCTATTCTTA
CTTTGTATATGGTACAACAAAATAGATGAAAAAAATATAGTTTGGTTTGCTCAACATGAT
CAAAATCCAGTCCCAAAGGGTTCAAAGGTGGAAGTAACTGCTTCAAATGGTTTATTGC
TTAAAAGCTCTCAAGGTGGAGAACTTTGGAAATCAGGCCCTATTTCAAGTGTGGTAGC
CTTTGGTACAATATATGATACAGGAAATTTGGTGCTTCTAGATTCAAATACTACTCCCTTA
TGGGAGAGTTTCAACCAACCTGTGGACACTTTGCTTCCTACTCAAAAAATGGAGGTAA
AGGACTTTCTTTCTTCGCGCAAATCACAAAATACTTACTCATTAGGAAAGTTCCAACTT
CGTTTCTCTGAAGGTAATCTTGTGCTCAATATGAGAAGTTTGCCCACCACGTATGCCTAT
GAACCTTACCATGTCATACAAGCTTTTGAGGGTAATCAAGTGGTCTTTGATGAAGATGG
TTTCTTGTATATAATCCAAAGAAATGGAAAGAGAGTAAACATTAGTGAACCCGAAAGT
GCTTACCCGGCTAACACTCATTATTACCAAGTCACTCTCAATTTTGATGGTGTGGTCACT
GTAAGTCACCATACAAGGAATCCCTCAGCTTTTAATGCAACATGGATTCACTTTAAGAC
TATACCGAATAATATATGTGTTGCTATGCGAGGAAACTTGAGCTCTGGAGCTTGTGGATA
CAATAGCATCTGCACATTAAACAATGATCAAAGGCCAAGTTGCAACTGCGCACCTGGTT
ATTCATTAATTGATCTGAATGATAAGTACAGCGACTGCAAACCAATTATCCAGCCAATAT
GTGAAGATGGTGAGAACAATTCTACCACTGATTTATATAGACTACAAGATCTTCCAAAT
ACTGATTGGCCAACTCAAGATTATGAGTTATTCAAGCCTTTTACAATTGAAGAGTGCAA
GAATGCTTGCTTGCTTGATTGCTTCTGTGTGGCAGTTGTATATAGAGATAATAGTTGTTG
GAAGAAGAAACTGCCCCTCGCTAATGGGAGGAAGGATAGCGGTGAAAAATCAATTTCT
TTCCTAAAACTAAGAAGAAACATTAGCTCTATCGGACAAGACTCCAATCTTCCAAGATC
TAAAGGAAAGAAGAACCACGACACATTAGTTCTTGCACTTTCTATATTGCTCAGTAGCT
CACTGTTGATCATTCTAGTATTGGCTAGTTTTATATCTCGAGGTTTCATCTCCCACCACA
GAAAGAAGCATACAAGTGATTTCCTACCAAGAGGAAATTTTGGTAGTATGCAGAAATTT
ACATTTAAAGAACTTAGAGAAGCTACAAATGGGTTTAAAGAAGAACTAGGACGAGGAT
CTTGTGGCGTTGTTTACAAAGGGGTAACTGAAGTTGGCTCTGTTGCTGTTAAGATATTC
AATGATATGTTTGAAGATAGTGAGAAAGAATTCAAAACTGAAGTGATTGTGGTTGGTG
AAGCACATCACAAAAATATCGCTCGACTACACGGATATTGTGATGATGGTAAAAGGTGC
ATGTTAGTTTATGAGTTTCTGAGCAATGGCAGCCTAGCAAGCTTTCTTTTTGGTGATTCA

AAGCTCAGTTGGGATCTCAGAACCAAAATAACCTATGGAATAGCAAGAGGCCTCTTGT
ACTTACACGAAGAATGCAACACTGAAATCATCCATTGCGATATAAAACCCCAAAATGTA
CTTCTTGATGAACACTACAATCCCAAAATTTCTGATTTTGGATTGGCTAAGCTATTGAAA
ATGGATCAGAGTAGAAATAGAGTTGAAACTAATATCAAAGGAACGACAGGGTATATCG
CTCCAGATTGGTTCAAGTCAACCCCGGTG
ACTACTAAGGTTGATGTGTATAGTTTTGGTGTCCTGATGCTAGAAATTATATGTTGTAGA
AGGAATGGAGATATGGAAGTTTATGAACAAGGAAGAGAAATATTGGTAGATTGGGCCT
ATGACTGCTATCAGCAAGGAAGATTAGATGTTTTAGTTGAAGGAGATTTTGAGGCCATT
GATGACATGGGGAGGTTAGAAAGGTTTGTGGTGGTTGCAATTTGGTGTATTCAAGAGG
ACCCATATCAAAGACCCACAATGAGACAGGTCATTCCTATGCTTGAAGGCATAGTTCCT
GTTTCTACTCCTCCAAGTCCCTGCTCATTCAGCTCCACCTCCTGA
> gene:Csa1G605740
ATGGCTTGTATAATTCCTCATATTTTTCTCTTTCTGCCTTCAATAATCTATGCTCAAAGTG
ATAGCATGTTATACATTGGCAGCTTTCTCATAGCTGGTGATCCTTCTTCTTCTCCATGGA
GATCTCCTGCTGATGAATTTGCATTTGGATTTAAACAAGTTGAAGGTGATCTCTTCTTGC
TTTCTATTTGGTACAACAAACTAGATGAAAAATCCATTGTTTGGTATGCTATACATGATC
AAAATCCAGCCCCAAGAGGTTCAAAATTGGAAGTAACTGCTTCAAATGGCTTATTGCT
TCAAAGTTCTCAAGGTGGAGAACCATGGAAACCAAGTCCAATTTCAGGTGTGGTCGCA
TTTGGTAAGATTAATGACGATGGCAATTTGGTGCTTCTAGATTCAAATTCTAATACCGTA
TGGGAGAGTTTCAAACAACCTGCAAACATTTTACTTCCTACTCAAACAATCGAGGTAA
ACGACTTACTTTCTTCACGCAAATCACAAAATAGTTACGCGTTAGGAAAGTTCCAACTT
CGGTTGTCTGAAGGTAATCTTGTCCTCAATATCATAAGTTTGCCCAGCACCTATACTTAT
GAACCTTACCATGTTATACAAGCCTACGAAGGTAACCAAATTGTGTTCGACAAAGGTG
GTTTCTTGTATATAATGCAAAAAAATGGAACGAGAGTAAATATTAGTGAACCCGAAAGC
GCATACCCAGCTAACACTCATTATTACCAAGTCACTCTCAATTTTGATGGTGTGATCACT
GTAAGTCACCATACAAGGAATCCCTCAGCTTTTAATGCAACATGGATGGACTTTAAGAA
GATACCACATAACATATGTGTTACTATGCGTGGAAACTATAGTTCGGGAATTTGTGGATA
CAATAGCATCTGCACATTAAACAATGACCAAAGGCCAAGTTGCAAGTGCCCACCTGGT
TATTCACTAATTGATCCGAACAATAAGTACAGCGACTGCAAGCCAAATATCCAGCCAAC
ATGTGAAGGTGATGAGAACAATTTAACCAACAATCTATATAGTCTACGAGTTCTTCCAA
ATACTAATTGGCCAACACAAGATTACGAGTTATTCTGGCCTTTTACAGTTGAAGAGTGC
AAGAATGCTTGCTTGCTTGATTGCTTCTGTGTGGTGGCTGTATATAGAGATAATAGTTGT
TGGAAGAAGAAGCTACCACTCTCTAATGGGAGAGAAGATAACAATGAAACATCTGTTT
CTTATTTAAAACTCAGTACCAGTTCCATTGGGCAAGGCTTTGATCTTCCAATGCCGAAA
GGAAAGAAGAAACCGAACACGTTAGTTCTGGTGCTTTCTACGTTACTTGGTAGCTTTGT
ATTGATCGTTCTCATATTGGTTAGTTTGATATGTCGGGGTTACACCTTCGACCACAAAAA
GCAACTTATGGGTAATTTCCATCCAAGAGAAAGCTTTGGAAGTAGCATGCAGAAGTTT
ACATTCAAAGAACTTAGTGAAGCTACAAACGAGTTCGAAGAAGAACTAGGAAGAGGA
TCTTGTGGCATTGTTTACAAAGGAACGATGGAAATCGGCCCTATTGCTGTTAAGAAATT
TCATATGTCTGAAGATGGTGAGAAAGAATTCAAAACTGAAATTAATGTGCTTGGTCAA
ACACATCACAAAAACATTGTTCGTCTATTCGGATACTGTGATGACAATAAAATCTACTTT
TTGATTTACGAGTTTATGAGCAATGACAACCTAGCAAGATTTCTTTTCAGTGATACGAA
GCCCAGTTGGGACATCAGAACCAAAATAACCTATGGAATTGCAAGAGGACTCTCGTAC
TTACACGACGAATGCAACACCCAAATCATACATTGCGATATAAAACCTCAAAATGTACT

TCTAGATGAATGCTACAATTCCAAAATTTCCGATTTCGGATTGGCAAAGCTACCGAAAA
TGGATCAAAGTAGAACCCGAATCGAAACCAACATCAAAGGAACGACAGGGTATATCGC
CCCAGATTGGTTCAAGTCGACCCTCGTGACAACC
AAGGTCGATGTGTATAGTTTTGGTGTCCTGCTGCTAGACATCATATGTTGTAGAAGGAAT
GGAGAAGATGTGGAAGTTTCTGAAGAAGGAAGGGAAATATTGGCGGATTGGGCCTATG
ATTGCTTTGAACAAGGAAGATTAAATGTTCTAGTTGAAGGAGATTTGGAGGCCATTGGT
GACAAGGAGAGGTTGGAAAGGTTTGTGAAAGTTGCAATTTGGTGTATTCAAGAGGAC
ACATCTCGAAGACCAACAATGAAAGAGGTAATGTATATGCTGGAAGAAGTAGTTCCTG
TTTCTACTCCTCCAAGTCCTTGCCCATTCAACTCCATTTGTTGA
> gene:Csa1G071160
ATGGCAAAACTGATTTCGATTTGTCTGTTTTTCTGGACGACCACCGCTCTGTTTCCAAG
AAAATCATTAGCAATTGATAGCATAAAAGCAGGGGAATCCATTAGTGCCAGTGCCCAGA
TATTAGTTTCAGCTCAACAGAAGTTTGTGTTGGGAATCTTCAATCCCGAAGGCTCCAAA
TTTAAATATTTGGGAATATGGTACAAGAACATCCCACAGAGGACTATCGTATGGGTTGC
AAACAGAGACAACCCATTTGTAAGTTCCTCTGCCAAATTAACATTCAATGAAGAAGGG
AACGTTATTCTTGTCGATGAAACAGATGGAGTTTTATGGTCTTCCACTTCTTCAATATAC
GTGAAAGAACCGGTCGCTCAACTTCTAGATAACGGTAACTTGGTATTAGGAGAATCTG
GGTCTGAAAATTATGTGTGGCAGAGTTTCGATTATGTCTCTGATACTCTGTTACCCGGCA
TGAAACTTGGTCGGGACTTGAAAGCTGGTATGACCTGGAAGTTAACGTCATGGAAAAA
CCAGAACGATCCCTCCTCTGGGGATTTCACTTATGTTATGGACCCTGGTGGGCTTCCCC
AGCTTGAAATTCACAGAGGAAACGTCACAACGTACAGGAGCGGCCCCTGGTTGGGTA
GTAGGTTTAGTGGCGGTTACTACCTTAGAGAAACGGCAATTATAACTCCACGGTTCGTT
AATAATTCGGATGAAGCATTCTATTCATACGAGTCTGCTAAAAATCTCACTGTCAGATAT
ACACTGAACGCAGAAGGCTACTTCAACCTATTTTATTGGAACGATGATGGAAATTATTG
GCAGAGTTTGTTTAAATCACCAGGAGACGCCTGTGATGACTACAGACTCTGTGGAAAT
TTTGGTATTTGTACGTTTTCTGTCATAGCTATCTGCGATTGCATTCCTGGGTTTCAACCA
AAATCGCCAGATGATTGGGAAAAGCAAGGCACGGCTGGTGGGTGCGTTAGAAGGGAC
AATAAGACCTGCAAAAATGGAGAGGGGTTTAAAAGAATCAGCAATGTGAAATTACCAG
ATTCTTCTGCGAAGAATTTGGTTAAGGTTAACACAAGCATTCAAGACTGCACAGCGGC
GTGCTTGAGTGATTGCTCTTGCTTGGCCTACGGAAGGATGGAGTTTTCCACAGGAGAC
AATGGCTGCATCATATGGTTTGAGAGATTGGTGGATATGAAAATGCTTCCTCAATATGGA
CAGGATATCTATGTAAGGTTGGCTGCTTCAGAATTAGAATCGCCTAAAAGGAAGCAGCT
TATAGTTGGGCTGAGCGTGTCCGTTGCTTCACTGATAAGCTTCTTGATTTTTGTTGCTTG
CTTTATCTATTGGCGTAAAAGAAGGAGGGTTGAGGGTAATGAGGTTGAGGCTCAAGAG
GACGAAGTTGAATTGCCACTCTATGATTTTGCGAAGATTGAGACTGCCACGAATTATTT
TTCTTTTTCAAATAAGATTGGCGAAGGTGGTTTCGGTCCTGTGTACAAAGGAATGATTC
CATTGGGACAAGAAATTGCAGTAAAAAGACTGGCAGAGGATGACAAGAAGCGATCTT
TACTTAGTTGGAAAAAAAGGATGGATATTATAATTGGAATAGCTCGAGGTCTTCTTTATC
TCCACCGAGATTCAAGGCTTATAGTTATACATAGGGATCTCAAAGTGAGTAACATCTTAC
TAGATAACGAAATGAATCCAAAAATTTCGGACTTCGGTATGGCTCGCATGTTCGGTGAA
GACCAAACCATGACACAAACTAAAAGAGTTGTTGGGACCTACTTTTTGACGTAA
> gene:Csa6G516770
ATGGGTAGTGATTGCAGAAAGGTTGTTGGGTTTCTTCAATTCTTTGTTATTTCATTCTTC
CTCTGTTCTTCGCCTCTGTTTTGCGATGCTGCAGATTCAATTACAAAGGGTAGAGGTTT

AAGAGACGGTAGTAATGAAACCCTCGTATCCCTGGATGACTCCTACGAACTGGGTTTCT
TCAGCCCCATAAATTCTTCACTACGATACGTTGGAATATGGTATCACAAGATCGAGGAA
CAGTCTGTTATTTGGGTTGCGAATAGAGATAGGCCACTCCGTAATAGAAATGGGGTTTT
GATAATCGGAGACGACGGGAACTTGGTCGTTCTGGACGGTAACAACTCTGTTTGGACA
AGCAATATCACAGCGAATTCGTTTGAGCCCAGAAACTTAACTCTTCTTAACCATGGAGC
ATTGGTTCTCTCGAGTGGAGACGACTTATCGAAAGTTCATTGGAGCAGCTTCGAACAC
CCCACGGATACATTTCTTCCCAATATGGTGGTGAAAGTGAACCCACAAATGGGCGAGA
AACGAATGTTTATGTCGTGGAAATCAGAGACAGATCCGGCCGTCGGAAATTACTGTCT
GGGTGTGGATCCCCGTGGAGCTGTGCAGATCATCGTTTGGAATGGGAATAATCGATGGT
GGAGAAGCGGCCACTGGGACAAGCAGATTTTCTCTGGGATTCCAACTATGCGTTCTAC
GTCGTTGTACGGGTTTAAGATTACCTCTGATGATGGAAATAACATTAGCGTAACTTTTGA
AGCACTGAATGATTTGGATAAGCTGAAATTTCAAATTCAGTGGGATGGTAAAGAAGCG
CAGCAACGGTTGAATGAAACAACTCGCAAATGGGATACCATTCGTTTACTGCCTTCGA
ACGATTGCGATTTCTATAATTTTTGTGGAGATTTTGGGGTTTGTTCTGAAAACAGTCGTC
TTAAGTGTAGCTGCCCTCAAGGGTTTATACCCAAAAACAAAGAACGATGGGACAAAGG
GATTTGGTCAGATGGGTGTCGGAGGAAGACTCCATTGCTTGAGCAGAGAATGAAAAGT
AGTCCAAACGGAACTATTGAAGATAGTGAGCAAGATGGATTTGTAGATGTGCTATTTGT
GAAATTGCCCGATTTCATAACTGGAATATTTGTGGTGGAGTCCTGTAGAGATAGATGTT
CCAGCAATTCTTCGTGTGTTGCATATTCCGATGCTCCTGGAATTGGGTGCGCTACTTGG
GATGGACCCTTAAAAGATATTCAGAGATTTGAGGGTGCTGGGAATACTTTGCACCTTCG
TATCGCTCATTCTGATTTGACACCTGTAGATAGCGAGAGCAAATTGTCAACTGGTGTGA
TAGTGGCGATATGTTTTGGAGGAGCAGCTGCCATAGCGATAATAGCATTGCTGCTATGG
AAATTCAGAGGCAAAACGAAAGCTGCTACTACAAGTGAACCTCAGAACAAAACTGAA
GTACCAATGTTTGACCTGAGCAAGAGCAAAGAACTTTCAGCAGAGCTTTCAGGGCCAT
ACGAATTAGGCATAGAAGGTGAAAATTTGAGTGGACCAGATTTGCCAATGTTCAATTTC
AACTGTATAGCTGCAGCTACCGATAACTTTTCTGAGGAAAACAAGCTTGGCCAAGGAG
GCTTCGGCCCTGTATACAAGGGAAAGCTTCCATGTGGACAAGAAATTGCTGTCAAGAG
GCTTTCAGTCCGGTCTGGCCAAGGTCTAGAAGAGTTTAAGAATGAGATTATACTAATTG
GAAAGTTACAGCACCGAAACCTCGTCAGATTGTTGGGCTACTGCATTCAAGGAGAGGA
CAAGTTGCTGCTCTATGAATATATGCCAAACAAAAGCTTGGACTGGTTTCTTTTCGATC
CAAACAAGCAGGCACTACTAGATTGGAAAAAACGGTTGTCAATCGTTGAGGGAATTGC
ACGAGGGCTGCTATACCTTCATCGAGACTCGAGACTTCTTATTATTCATAGAGATTTGAA
AGCTAGCAATATTTTACTAGACGAAGACATGAATCCAAAGATATCGGACTTTGGCATGG
CTAGAATATTTGGTGGAAACCAAAATGAGGCGACGAATACAATTCGAGTTGTTGGCAC
ATACTATGTAAGCTTGGAAACTTTGGAATGA
> gene:Csa6G052130
ATGAACTTCCATACCCATTTGTTTCCTTGCTTTATTGCTTTTGCTTTTGCAGTGTCTTTCT
CGGAGGCTGCTATAACTCTAGGTTCTTCTTTGAGAGCTTCGGATCCCAATCAGGCCTGG
AATTCCTCTAATGGAGATTTCTCTTTGAGTTTCACTCCATTGGGTTCTTCTTCTTTTAAA
GCCGGCATAGTCTTCACTGGTGGCGTTCCTACTATATGGTCTGCTGGTGGCGGCGCTAC
AGTGGATGCTTCCAGTGCTCTTCACTTTCAATCTGACGGTAACCTCCGTCTCGTCAGTG
GTTCTGGTGCCGTTGTTTGGGAATCTCACACTACTGGCCTTGGCGTTTCCTCCGCTGTG
CTCGAGGATACCGGCAACCTCGTTCTTCTAAATAGTAGTTCCCAGCCGGTTTGGTCTTC
GTTCGATCATCCGACGGATACGATTGTTCCATCGCAGAATTTTACTTTGGGAATGGTACT

GCGATCTGGTCAGTATTCTTTTAAACTACTTGATGTTGGGAATATAACTCTGACTTGGAA
TGGAGATGAAGGTGATGTTATTTATTGGAATCATGGGTTAAATACATCGATCGGTGGAA
CTTTGAATTCTCCTAGTTTACGCTTACATTCTATTGGGATGTTGGCTGTTTTTGATACAAG
AATACCAGCTGGTTCATTTGTAGCTTATAGCAATGATTATGCTGAAAATGCTGAGACTAC
TTTTAGGTTTCTAAAGTTAACAAGTGATGGAAATTTAGAGATTCATAGTGTTGTTAGAG
GAAGTGGGTCTGAAACAACAGGATGGGAAGCTGTTTCTGATAGATGTCAGATATTTGG
GTTTTGTGGGGAACTTAGTATTTGTAGTTATAATGATAGAAGTCCAATTTGTAATTGCCC
ATCTGCAAATTTTGAACCTTTTGATTCAAATGATTGGAAGAAAGGATGTAAGAGGAAG
TTGGATCTCGGAAATTGTAGCAATGGCATTAACATGTTACCATTGGAGAATACAAAGCT
TTTACAATATCCATGGAATTTTACAGGCATACAACAGTACTCCATGCAGATATCGGGGTG
TCAATCGAATTGTCGACAAAGTGCTGCCTGTGATTCTTCTACTGCACCATCAGATGGGA
GTGGGTTTTGTTATTACATACCTTCAGGTTTTATTAGGGGATATCAAAGTCCTGCTTTGC
CAAGCACTTCATTCCTCAAGGTCTGTGGGGATGTGGACCTAAACCAACTGGAATCTTC
TGATGTTTCGAGGCCAGGTGATAAGGTGAAGGTTTGGGTTTTGGCTGTTGTGGTTTTGG
TTACCCTTTTTGCCATGATTGCTTTTGAGGCTGGTTTATGGTGGTGGTGTTGTAGACACA
CCTCCAATTTTGGAGGGATGTCCAGCCAATATACTCTTCTTGAGTATGCTTCTGGTGCTC
CAGTGCAGTTCTCATATAAAGAACTCCATCGTGTGACGAACGGTTTCAAGGACAAGCT
TGGAGCTGGTGGATTTGGTGCTGTTTATAAAGGGGTTCTTACTAATAGGACAGTTGTGG
CAGTGAAACAACTCGAGGGAATTGAGCAGGGAGAGAAGCAATTTAGGATGGAGGTAG
CAACTATAAGTAGCACACACCATTTGAATCTGGTGAGATTGGTTGGTTTTTGCTCAGAA
GGACGGCGTAGGCTATTAGTATATGAGCTCATGAAGAATGGCTCTCTTGATGGATTGATA
TTCAAGGGAGAAGAAGGGCAATCTGGGAAGTTCCTTAGCTGGGAAGATCAAACAAAT
CACAAGCGGTTCTCTTTGTGGGCTTACGAAGAGTTCGAAAAAGGAAATCTCATAGAAA
TTGTTGACAAAAGACTTGTGGATCAAGAGATTGATATGGACCAAGTTAGTAGGGTGGT
TCAGGTGAGCTTTTGGTGCATCCAGGAGCAACCGTCTCAGAGGCCAACGATGGGAAA
AGTCGTGCAGATGATCGATGGAGTCATCGACATCGAGAGGCCTCCTGCACCAAAAGTA
ACATCCATGGTTTCCACAAGTGGAACAACAAGCACTTATATCAGCAGCAATTTGAGCA
ATTTCTCCACCACACCCACAACCGAAACACCGGCC
TCATTTTCGTCATCTCATGCTGCACTAGACATGACTCCTGGTGGCAGCAAGATTGAGAA
AACAAGTTCATCCCTCCTCCAATCAAGGTACGACTAA
> gene:Csa3G733880
ATGTTCTTTTCTTTTCCGTTTCTTTCTTCATTACTTCTTGCATCCACCGCCGTTTGGGCGG
CTGCTTCGGCGGGGTTGCAGAGTCTTACTCCTGGAAACTCCATCGCCGTTGAGGATGA
GAATCAGTTCCTAATATCACCAAATGGAACTTTTTCATCTGGGTTTTATCCTGTGGGCAA
CAATTCTTATTGCTATTCCATTTGGTACACAAAAAGCTTCGAGAAAACTGTAGTATGGAT
GGCAAATCGAGACAAACCAGTTAACGGTGCGAAATCCAGATTGACACTCAACATCGAT
TCCAATTTGGTTTTAACTGATGCAGATGGCACAATCGTTTGGTCCACCGATACAGTTTCT
AATGGTGAAATTCAACTCCGACTTCTTGAAACTGGAAATCTCGTAGTAATGAATCAATC
CCAAAATTTCATTTGGCAGAGCTTTGATTTCCCTACAGATACTCTGCTTCCACAACAGA
GGTTTCTCAAGACATCAACTTTAGTCTCAATGCAAAATCGAGGTGTATATTTATCAGGAT
TTTACTTCTTCAAATTCAACGATTACAATGTATTGAATCTCTTATACAACAGTCCTTCACT
TTCCGGTATCTATTGGCCTGATACAATGGTGACGGTTTTCGTCAATGGTAGATCTCCATA
TAACAGCTCCAGAATTGCAATTCTAGACGAAATGGGTGGGTTTGAATCCAGTGACAAG
TTGAAATTCAATGCCACGGATTATGGGTTAGGTCCGAAGAGGAGATTAACGGTTGATTT

TGATGGGGTTTTGAGATTATATAGCTTGGTTGAATCAACTGGCAACTGGACAGTCACGT
GGATTCCTTCAGGTGCGCGTATCGATCCCTGTTTGGTTCATGGGTTATGTGGGGATTATG
GTATTTGTGAATATGACCCATTACCGACTTGTTCTTGCCCTCCTGGTTTCATTAGAAACG
ATCCTTCAGATTGGACTAAAGGGTGTAAACCACTTGTGAATTTGACTTGTAATTCTATTA
ATCCTTCCAAAGAAATGGATTTCATTGCTCTTCCTAATACGGATTACTTCGGCCATGATT
GGGGTTATGTAGATAAATTCTCCATTGAAATGTGTAAGGATTGGTGTCTTAGCAGCTGC
GAGTGTACAGGATTTGGGTACGCACTCGATGGTACAGGACAATGTTATCCCAAAATGG
CTCTTCGTAATGGGTATCGAAAGCCCAGTACGGCTGTGCGTATGTTTATTAAGGTCACG
AAAGATGAGTACTCATTGTCGTTGGCGCTACGACATTCAACAAATGAATTGAATTGCTC
CGTTTCACAGATTGTTTTAGGGACAGAGCATGTGTATGCAGAGAAGAGTAATAAGTTTC
GATCAATGGGATTGTTAGTTGGAGTGGTGGTTGCTATTGGGATTAGTGAACTCATTTTC
GTTGGTTTTGGTTGGTGGAATGTCTTCCGTAAACGAGTGAATGAAGAATTGGTTAATAT
GGGTTACATTGTATTAGCCATGGGATTCAAAAGATTTTCATACGATGAACTGAAAAGAG
CTACTAAAAATTTCAAGCAAGAGATCGGGAAAGGAGGGTTTGGAACTGTTTACAAAG
GAGAATTGGATGATGGAAGAGTTGTGGCAGTGAAGAGATTGGATGGTGTTTTACAAGG
AGAAGCAGAGTTTTGGGCTGAAGTTAGCATAATTGGGAAGATTAATCACAAAAACTTG
GTGAAATTATGGGGTTTTTGTGCAGATAAACACCATAAAATGTTGGTTTATGAGTATGTA
AAAAATGGGTCATTGGACAAATTTCTATTCTCTGATTCATCTCAAGTATTGGGATTAGAA
CAGAGATATGAAATTGCAGTTGGAACAGCAAAAGGGTTGTCTTATTTACATGAAGAAT
GTCTTGAGTGGGTTCTTCATTGTGACGTCAAGCCTCAAAATATACTTCTTGACGAATCC
ATGGAGCCAAAAGTTGCTGATTTCGGGATGTCGAAGCTGTTTAGAGAGATTAATGAAA
GTGGTTTCTCAAAGGTGAGAGGGACCAGAGGTTATTTAGCTCCAGAGTGGATGATGAA
TCTTAAAATTGATGCAAAGGCAGATGTTTACAGTTATGGGATTGTTGTATTGGAACTTTT
AAGTGGAAAGACTGCATACGGTTTTGAATCGTCTACTGTTTGTAAAGATGGTGGAAGG
AATATAGATATGGTGAAATGGGTAATGGAAGTTGCAGAGAAAGGTGAGGTTGAGAAAG
TGATGGATCCAAGATTGAAGGTGGAAGATAAGCAAAATAAGAAGAAGATTGACATATT
GTTGAAAGTGGCTTTGTTATGTGTGAAGGAAGATAGAAATATGAGGCCTGCTATGAGTA
GAGTTGTTGAACTTCTTACTGGCTACGAAGAACCAAGCTCACATGGAGATGTTTGTTG
A
> gene:Csa3G733860
ATGTTTATCTCTGCCCTTCTAATTTCTCTCCTTCTTTCACCATCCTTGGCTTGGCCTGAAG
GAACGACGACCTTAACTCAAGGAAACTCCATTGACGTTGAGGACGAGAATCAGTTCTT
AACTTCCACAAATGGAATATTTTCTTCTGGGTTTTACAAAGTGGGCAACAATTCCTTCT
CCTTTTCAATATGGTTCGCAAGATCTGCTGATAAGACCGTCGTTTGGATGGCAAACAGA
GACAACCCAGTTAACGGAAAGCAATCAAAATTGAGACTCAACTTCAACGGAAATTTG
GTACTCACCGATGCCGATGGTTCGTTCACATGGTCTACCAACACCATCACTACACAGCA
AGTTGAGCTTAAGCTTCTTGACAATGGAAATCTTGTACTGGTGAATCAAATTGGGGTTT
TTCTTTGGCAGAGCTTCGACTTCCCAACAGATACTCTGCTTCCACAGCAACAATTTCTC
AAGAATTCCACTTTAGTCTCCATTAAAACTCCAGGTACTTACTCATCTGGGTTTTATTTC
TTCAAGTTCAATGACGACAATGTTCTAAATATCATTTATAATAGCCCTTCGCTTTCTAGTA
TCTACTGGCCTGATCCTGGTAAGAATGTGTTTGATAATGGTCGAAGTCGTTATAACAGTT
CCAGATAGCCATTTTGAACGATATGGGGAGGTTTGAATCCACTGATAACTTGAATTTCA
ATGCTATTGATTATGGGTTTGGTCCAAAGAGGAGATTAACCATGGATTTCGATGGAGTTT
TGAGATTGTACAGCCTTGTTGAATCAACTGGCAGTTGGGAAATCACTTGGCTTCCTGAT

GGACCGTTAGATGCTTGTTTGGTTCATGGGTTGTGTGGAGAATTTGGGATTTGTTCGTAT
ACTCCATTACCTACGTGCATTTGCCCCCCGGGTTTCATTAGAAACCATCCATCGGATTGG
AGTAAGGGCTGCAAACCTTCTTTTAATTTGAGCTGTGATTCCAAGGATTTGGATTTCAT
TCAACTTCCTCGTACGGATTATTATGGTTATGATTTGGTGGGTTTCGCCAGAGGAGTCA
GTGTTGAAACTTGTCGGAACTCTTGTCTCAACAGTTGTCAATGTTTGGGGTTTGGATAT
TCAACGGATGGCTTAGGACTATGCTTTCCTAAAGGAGTTCTTCGTAATGGGAATCGAAA
ACCCGATACCATGAGACTAATGCATATCAAAATTCCAAAGGGCAGACCAAAAACGGAG
TTGAAGGAAGAATTTTCAAATGATTTGAAATGCTCTGCTTCAGAAATTGTTCGAAACAC
AGAAATATTCCCAGAAAACAAAATTAAATTTCGGTATATGGGATTATTGATTGCGTTTGT
AGCCATTGCTGGGTTCATTGAGTTAATTTTCTTCGGTTTCGGGTGGTGGAATGTCTTTCG
AAAGCGAGTTAACGAAGAGTTGGTTAACATGGGTTACATTGTTTTAGCCATGGGATTCA
AAAGATTCACATACGCAGAAATGAAGAGAGCAACAAGAAATTTCAAACAAGTAATAG
GAAAAGGAGGATTTGGAACAGTTTATAGAGGAGAATTAGACGATGGAAGAATCGTGGC
AGTGAAGAGATTGGAAGGCATTTTACAAGGAGATGCAGAGTTTTGGGCAGAAGTTAG
CATAATCGGAAAGATAAACCACAAGAACTTAGTGAAATTATGGGGTTTTTGTGCTGAG
GAAAAGCATAAGATATTAGTTTATGAGTTCGTGAAAAATGGGTCTTTAGATAAACTTCTA
TTCTCCAATAATTCATCACAACCATTGGGATTGGAACAGAGATATGAAATAGCAGTTGG
AACAGCGAAGGGTTTAGCGTATTTACACGAAGAATGTCTAGAATGGGTTCTTCATTGTG
ATGTGAAGCCTCAAAACATACTTCTTGATGAAGAGTTAGAACCAAAAGTGGCAGATTT
TGGAATGTCGAAGCTATTTAAGGAGATCGATGAAAATGGATTCTCAAGAGTGAGAGGG
ACAAGGGGTTATTTGGCTCCAGAATGGATGATGGATCAAAAAATTGATGCAAAGGCAG
ATGTTTACAGTTATGGGATTGTTTTGTTGGAGCTTGTGAGTGGAAAATCTGCATCTAATT
TTCAATCATCTTCAAATTCTATGGATTTCAGATACAGTAATTTAGTGAGTTGGATGATTG
ATAATGTGGAGAAAGGGAAGATGGAGGATGCGATTGATCCTAGATTGGAAGAAAGTGA
GAAGGATGTTAGAAAGATTGAGATGTTGCGCCACTTTTTGGAGTTAAAAATAGAGGCA
GAGGAAACACCAGAATCTCATCGCATATCTATATCGTATTACTTCTTCATTCCCTTTCCG
GCTTTCCTCCGCCGTCTCCCCCACCCTCACCGCCTATATAGTTTATTCTATTCCATCATTT
GGATACTGGCGGCTTCCGTAATCGAATTTGTAACCGCTACCGTTTCTTTTTTTGGTTTCT
TTGGGAAAGCTGAGTTATAA
> gene:Csa3G099580
ATGGACTCTTTTTCTGCAACACTCCTTGCTTTTAACTTAGCCCTTCTTCTCTTCAGGTCT
GCAGCAATCTCCGATTCCTTAACGGCTCAAAACCCATATCTCAGAGATGGCCTTAGTCT
GGTCTCTACTAATGGAACTTTTGAATTGGGTTTCTTTAGTCCTGGACTTCCCAGTAACC
GTTACTTGGGAATTTGGTACAAGAATCGCCGAGGTCCAACATCTGTTTGGGTTGCCAAT
AGGAAAACCCCCATTAGTGGTTCGTCTGGTGTACTGGTGATGAACATTACAACAGGAA
ATCTTACACTCTTCGGCCATAATTCCACTGTTGTTGTTTGGTCCGCCAGGTTACTGAGA
AAAGTCCCCAATGGGGTACTCCAACTATTGGACACTGGAAATCTTGTGCTGAGAGATA
GGGATGATGAAAATCCCCAAAACTATTCCTGGCAAAGCTTTGATTACCCTTCTGATACT
CTCTTGCCTGGAATGAAGCTAGGTTGGGACTTGAGATATAACATTGAGAGAAGATTAG
AGGCTTGGAACAATCTCAATGACCCATCTCCTGGAGACCTCAGTTGGAGGATGGAGCT
TCATGAATATCCCGAGACTGTCATGTGGAAATGTTCCCGAAAGTACGTTAGGCATGGCC
CATGGAATGGTGTGAGACTCAGTAGTAGACCACTAGCTGCAGCGCCAATTTTGAACTT
CAATTTTGTTTCAAATGAGAATGAGGTTTACTACCAAATCTCTGTTGTAAATAAGTCTCA
TTCAGTGATGTTGGTGATGAACCAATCGACTTACACGCGCATATTATACTTGTGGTCTGC

AGCTGAAAGACGGTGGAGAGTTTACACTTCATTGCCAAGAGATTACTGTGATAATTATG
CCCTGTGTGGCCCATATGGTTATTGTGATATAAGGGTAACTCCATCTTGTAAATGTCTAG
AAGGGTTTAAGCCAAGATCGCCTGATACTTGGAAAACTGGGGAATTTGCAGACGGTTG
TGAACGAAACAAACTGATGAACTGCGGTGATGAAGTTGGGTTTGCACATCTCAACCAG
ATGAAGTTGCCAGATACAAAGCATACTTGGGTCAACAAAAGCATGAATCTTGAAGAGT
GCAGGCAAAAATGCTTGAGAAATTGCTCATGTATGGCTTATGCAAATACAAATATTAGC
GGCAGTGGCAGTGGCAGTGGTTGTGCCTTGTGGATTGGTGATCTAATTGACTTGAAAC
TGATCCCCGATGCAGGACAGGATTTATATGTCAGAATGCTGGCATCAGAACTAGTCATG
CATAGGAAAGCACACAAGACTGGAAGATTGAATTCGAAAGTGAAGATTGCTTTGTTTG
CAATTTCCGGTTTTGGTTTGGCTATCATCTTCTTCATAGGCGTATACATTTTCAGAAGGA
GATCAACCATTAAAGATGGCCATGAGAAAATAGAAGCTCATCACCTGGAGCTTCCCTT
GTTTGATTTATCCTTGATTAACAGTGCCACAGATAACTTCTCACTTAATAATAAGCTAGG
AGAAGGTGGCTTTGGGCCAGTATATAAGAAATATATATATAAAAGTTTGAATTCCAGGGT
AAGCTTACAAATGGACAAGATGTTGCACATATGGTGTCTTGGTGTTTACCTTTCATTTTT
ACTGGACTTAGTAAATTTTAAATTGACAGATAAAACACAGCGACAATTATTAAGCTGGT
CAAATCGGTATCGCATTATTTGTGGAGTTGCTAGAGGACTCATGTATCTCCATCAAGATT
CGAGATTGAGGATTATACATAGAGATTTAAAAGCAAGTAATGTTTTACTTGACATGGATA
TGAATCCAAAAATCTCTGATTTTGGACTGGCTAAAACTTGTGGTGGAGATCAAACGGG
AGGACGAACATTAAGAGTGATTGGAACCTATTGTTTATTACAATTCTTCTATTCAAAGTT
TTATGATTGGAAAAAAAATTCAACCCTACATTTCTTGAATGATAGGCATGGCGATTATGG
AAAGAAGGAAATATGTATCAATATCAGTTTGTTATGTGTTCAACAACATCCTAATGATCG
ACCCACAATGTCATCGGTGGTTATGATGCTAGGCTGTGAAATTCCTTTGTCACAACCAA
AACAACCCGGATTTTTCATAGAAAATGAAGGCATTGAAATGAAAAGATGCTCAAGTGA
AGATAAATCAACGTCAACTAATGAATTGACTGTTACGCTCCCAGATCCAAGGTAA
> gene:Csa4G289630
ATGGCTTCTCATCAGAATGCATTCTCCTTCTTCTTCCTTTTTCTTTTTCTCTTTTTACCAT
CCTTTTCAGTTTCTGCTCAGCCATATAAAAATGTAACTCTAGGCTCAACTCTCACTGCA
CTCAACAACAACAATGACTCCTATTGGTCCTCCCTTTCTGGTGACTTTGCTTTTGGGTT
CCTACAATTTGAAAGCAAAGGCTTTTTGTTAGCCATTTGGTTCAACAAAATACCTCAAC
AAACCATTGTTTGGTCAGCCAAACCAAGTGCTTTGGTACCTGCAGGATCCACAGTTCA
ACTTACCAACACTCAACTTGTTCTCAAAGACCCTGCAGGCAAACAGATATGGAGTAGT
AATGATAATAATAATGTGGGACTTGGATCAGTTTCTTATGCTGCCATACTTGATACTGGC
AATTTCATTCTTACTGCCACCGATTCCCAAGTTTTGTGGCAAAGCTTTGATCATCCTACT
GATACAATTTTACCATCACAGACTTTGAATTCAAATCTTGTTTCTTCTTATTCAAAAACC
AACTACACTGAAGGAAGATTTCTTTTCTCAATGGGAACTGATGGAAATCTTGTTTCATC
TTACCCCAGAATCGTCCCTATGCGTTGGTCTCCCTTAATTTATTGGGAAAGTGAAACATC
AGGCTCTGGCTTCAATCTTGTTTTCAATCTCTCTGGCTCCATTTATATCTCTGCACCAAA
TGGAAGCGTAGTTAAAAATTTGTCCTCCAATACACCTTCAACTGACGATTTCTACCATC
GAGCTATTTTGGAATACGATGGAGTTTTCAGGCAATACGTGTATCCAAAGACTGCTAAG
ATTACAGGCAATGCCACTCCTTCTCCTTGGCCCAAGGATTGGAGTCAGGTCTCAGATTC
CATTCCTCCAAATATGTGTTTGCCAATAACCAATGGCTTAGGAAGTGGAGCATGTGGGT
ACAATAGCTATTGTAGAATTGGGGATGATCAAAGACCAACTTGCCATTGTCCTCAAGGC
TATGATTTACTTGATCCAAATGATGAAATTCAAGGTTGTAAACCAATTTTCACTCCTCAA
AGCTGTGATGATGAAGAAACTGATGCCTTTGAATTCTTTTCAATTGAAAATTCAGATTG

GCCTGATGCTGATTATGAGGCTTTCTATGGTGTAAATGAGGATTGGTGTAGAAGAGTTT
GTTTGGATGATTGTTATTGTTCTGCGGTTGTTTTTAGAGGGACGCATTGTTGGAAGAAG
AAATTTCCTCTCTCATTTGGAAGAATTGATCTGGAGTTTAAAGGTAAAGCTTTGATTAA
AGTTAGGAAACAGAACTCAACCTCCATAATTGTTAATCAAGCCTACAAGAAAGTGAAG
GACAAGACTTTGGTCCTTGTTGGGTCAATTTTCTTAGGGACTTGTGGATTTCTGATTGC
TACTCTTCTGATTGCTTACCAATTCAATATAAAGAGAACAGAGCTACTCATTGAGAAAA
ACTTGCCAGTACTCCAAGGTATGAACTTGAGAATTTTCAGCTATGAAGAGCTTCACAA
AGCCACAAGTGGATTCACAGAAAAATTGGGAAGTGGTGCTTTTGCTACAGTTTATAAA
GGGGTTATTGATGATTGTATGGACAAGGAGATTAAAAACTTGGTAGCTGTTAAAAAATT
GGAGAATATGGTGAAAGAAGGAGACCAAGAATTCAAAGCTGAAGGTTCATTAGCGGA
TTATCTTTTTGGATGTACAAAAAAACCAAATTGGTATGAGAGAATTGAAGTGATTTTAG
GAACAGCAAGAGGGCTATGCTATCTACACGAAGAATGGTATGTGGCTCCAGAGTGGTT
CAGAAATCTTGCCATAACAACAAAGGTAGATGTTTATAGTTTTGGGATTGTGTTGTTGG
AGATTATCAGTTGCAGAAAGAGTTTGGAGGTAGAGGGAGAAGATGAGTTAGTGGTGTT
GGCAGATTTGGCTTATGATTGGTTCCAAGAGAGGAAATTAGAGATGTTGGTGAGGAAT
GATGAGGAAGCGAAAGAAGACATGAAAAGAGTGGAGAAGTTTGTTAAGATTGCAATT
TGGTGCGTTCAAGAAGAGCCATCATTTAGACCATCCATGAAGAAAGTGGTGCAAATGC
TTGAAGGTGCTGTTGAAGTTTCCACTCCACCCCACCCATATTCATTTATCACTGCAATTC
ATTAA
> gene:Csa_4G290150
ATGGCCTCACTCTGCTTTCCATCCCCATCTCTTCTTCTACTTCTTCTACTTCTTCTTACCC
CAGCTTTTACTGTTGCTCAGACAACAAACCCAAATATAACTTTAGGACAATCTCTCACT
GCCCATTCCGCCAATTCCTTCTGGTCCTCTGCTTCTGGTGATTTTGCTTTCGGTTTCCGT
CAATCTGGAGGGGGTGACTATTTGTTAGCAATTTGGTTCAACAAAATTTATGATAAAAC
AGTTGTTTGGTCTGCCAATCGGAACAAACTGGCGCCGGAGGGTTCCACCGTTCTACTT
ACAACAACTGGTCAACTCCTTCTCAACGACCCTGCCGGCAACCTAATCTGGGCTTCAC
CTACTAATCAATCTGTTTCCTTTGCTGCTCTTCTCGATAATGGAAATTTCATTCTCGCTGC
AAACAATTCTGAAATTGTGTGGCAAAGCTTCGACTACCCAACCGATACTATTTTACCGT
CACAGATTCTTAACCAGGGCGACAGTCTGGTTGCGTCTTATTCAGAAACCAATTACTCA
AGTGGGAGATTTGAATTTTCTGTGCAACCTGATGGGAATGTCATGCTTTACACCAGAAA
CTTCCCTTCCGAATTAATAAGTCAAGCTTATTGGTCAACTGGCACTGTGAGCTTCGGCT
TTCAGGTC
GTCTTCAACCTCTCTGGTTCTATTGTTCTCATCGCAGAGAATAAAACCATTCTCAACAC
TCTCTCATCAAATAATCCCACGGCTCAAACTTTCTACCAGCGAGCAATTTTGGATCATG
ATGGGGTTTTCAGACATTATATTTATCGAAAGGGAGACACTGGAAGCACTTCATCTTGG
CCTAAAGCTTGGTCGTTGTCGAAATCTATTCCTTCCAACATTTGTTTGGCAATTAGTCAA
GGTTCGGACAGTGGAGCTTGTGGGTTCAATAGCTACTGCAGGCTTGGGGATGATCAAA
AGCCATTTTGCAGTTGTCCAGAAGGGTATGCCTTGTTTGATCCAAATGATGTAACACGA
AGTTGTAAACCCAATTTTGTTCCTCAAAGCTGTGATAAATCGTTTCCTGAAACTGATGA
CTTCTATTTCGTTTCTATGGACAATACGGATTGGCTTCTAGGCGATTACGGCCATTACCT
ACCAGTCAATGAAGATTGGTGCAGAAACGAGTGTTTAAATGATTGTTTTTGTGCGGCA
GCCATTTTTAGAGATGGGAATTGTTGGAAGAAGAAGTTCCCACTTTCATTTGGGAGAAT
GGATTACAGCGTGGGAGGAAAAGCTCTTATCAAAGTCAGGAGAGGCAATTCAACTCTA
CAATCTCAAAACCTTGACAGGAATTGCAATAACAAAACTAAGATAATCATTGGATCAGT

TTTGTTAGGAAGCTCTTTATTTCTTAATATCCTCTTATTTCTACTTACTTTATTGATCAGTT
ATCGATTTAGCAAAAGGAAATTATTAAAGTTTAATGGAGGAGACCCTTTCATTTTAGGT
GTGAATCTAAGAGCTTTTAGCTATGAAGAGCTGAACAAAGCCACTAAGGGATTCAAAG
AGCAACTGGGAAGTGGTGCATTTGCTACTGTTTATAAAGGGACGACTCTTGGTTCTGTT
GATGACAACAACTTGGTGGCAGTTAAAAAGTTGGAGAATATAGTGAATGAAGGATCAG
GAGAGAATGAATTTAAAGCTGAAGTAAGTGCTATTGCTCGAACAAACCATAAGAATTT
GGTTAAGTTAGTTGGTTTTTGTAACGAAGGAGAACACAGAATGTTGGTTTATGAGTTCA
TGGAAAATGGGTCTCTTGCTGATTTTCTCTTTAAGCCTTCAAGACCAACTTGGTATAGA
AGAATTCAACTTGTTTTAGGAATTGCACGAGGGTTAAGCTACTTACATGAAGAGTGCA
GGAAAATGGAGAAGTTGATAAGAGAAGATGAAGAAGCAAGGAGTGATATGAAGAGAG
TGGAGAGGTTTGTTAAGATTGGAATATGGTGTATTCAGGAGGATCCATCGCTAAGGCCA
TCCATGAAGAAAGTGATACAATTGCTTGAAGGAGATAAAGAGACATGGTGTATGTGGA
TCAGTTCTTGTTTCAAAACTTAA
> gene:Csa4G289640
ATGGCTTTCCCCTCTTTCTTTCTTCTTCTTCTTCTTCCTTTGTTTTCTTTACCCTCCTTTTC
ATTTTCCCAGCCATACAAAAATATAACTCTAGGCTCATCTCTCATTGCATCTCCACGCAA
CCACACCAATCATTCTTACTGGTCCTCCCCTTCTGGCGACTTCGCTTTCGGTTTCTTAGA
CACTGGAACTAATGGCTTTCTACTTGCTATTTGGTTCAACAAAATCCCTGAAAACACCA
TCGTCTGGTCCGCTAATCCCAACCATTTAGTCCCCTCTGGATCCATTCTCCAACTCACCA
CTCATGGTCAGCTGGTGCTTAATGACTCAGCAGCCAACCAAATATGGGCCGCAAATTTC
CAAACTGAAAACACAACGGTTTCCCATGCTGCCATGCTTGACACCGGCAACTTCATCC
TTGCCGCTGCTAACAACAATTCCCAAGTAGTTCTATGGCAGTCCTTTGATGAACCCACT
GATACTATTTTACCATCACAGGTTATGAAACCGGACACCATTCTCATTGCTCGCTTTTCA
AAAACAAATTACTCAGACGGAAGATTTCATTTGAGGATGGAATCTGATGGGAATCTCGT
GCTGTATACAAGAATTGTTCCTCTGGGTTCTCAAGGGAATCCTTATTGGTCAAGTAACA
CCGTGGGTTCTGGGTTTAACCTTGTTTTTGATTTGTCTGGCTCCATTTATGTCTCTGCAA
AAAATGGAACTGCTTTAACCTATTTAACCTCAAAAAATCCTTCCTCAAATCAACACAAT
TTCTACCATCGAGCCATTTTTGAGTACGATGGTGTTTTCAGGCAATACATTTACTCCAAG
AGTGATGAGGCTTGGAAGAGTGTTTCAGATTTCATTCCTTTGAATATTTGTGCGAGTATA
AATAATGGGTTAGGAAGTGGAGTTTGTGGGTATAATAGTTATTGTGTAACAGGGGAAGA
TCAGAGACCAATTTGCAAATGCCCACAAGGGTATTACATGGTTGATCCAAATGATGAGA
TGCAAGGTTGCAGACCAAGTTTTATTCCTCAAATATGCTCACTTGCTGAAGCAAATTCA
TTCGATTTTTTTTCTATTGAGAGATCAGATTGGACTGATTCTGATTATGAAGGTTACTCA
GGAACAAATGAGGATTGGTGCAGAAGGGCTTGTTTGGATGATTGTTTCTGTGCTGCGG
TCGTTTTCGAAACCGGAAATTGTTGGAAGAAGAAGTTTCCTCTTTCATTTGGTAGAGTT
AATCCTGATTTTAGAGGTAAAGCTTTGATCAAAATTAGAAGGGATAACTCTACTTTGAT
AGATGATAATCTTGTTAAGAGAGGAAAGGACAAAACTTTGTTAATAATTGGATTAGTCC
TGTTGGGTAGTTCTGGATTTCTAATCTTCATTTCATTGCTGGCTGTTCTAATTGTTTACCG
TATAAAGAAAAAGAGATCCGAAGGTGTTATGGGAAAGGTAGCAGCCTCCATAGGTGTG
AATGTAAGAGCTTTCAGCTATGAAGAACTAAACAAAGCCACAAATGGATTTACAGAGA
AGTTGGGCAGTGGTGCTTTTGCCACTGTTTATAAAGGGATTCTTGATGATGATGATTGT
CTGGACAAGGACAACAAATTGGTAGCTGTTAAGAAGTTGGAGATTGAAGTGAAAGAA
GGAGAGCAAGAATTTAAAGCCGAGGTGAGCGCGATTGCTCGAACAAACCACAAGAAT
TTGGTTCGACTGCTCGGTTTCTGCAACGAACATCTACACAGACTGATAGTGTATGAGTT

TATGCCTAATGGGTGTCTTGCAGATTTTCTTTTTGGGCCATCCCAATTAAATTGGTACGA
GAGAATTCAACTAGCTAGAGAAACAGCTAGAGGGCTTTGTTATTTACATGAAGAATGC
AAAACTCAGATTATTCATTGTGATATTAAGCCTCAAAACATCCTTTTAGATGAGTCATTG
AGGGCAAGAATATCAGACTTCGGTTTGGCGAAACTTTTGAAGGAAAATCAAACTCGAA
CCACGACTGCAATAAGAGGAACTAAAGGGTATGTGGCTCCAGAATGGTTTAGATCAAA
CCTTCCCATTACAGTGAAAGTTGATGTTTATAGCTTTGGGATCGTGTTGTTGGAGATTAT
AAGTTGTAGAAGAAGTTTTGAGTTGGAAGTTGAGGATGAAAATGAGATGGTGTTGGCT
GATTGGGCTTATGATTGCTTCAAAGAGAGGAGAGTTGATATGTTAGTAAGAAAAGATGA
TGATGAAGCAAAGGGTGATATGAAAACAGTGGAGAAATTGGTTATGATTGCAATTTGG
TGCATTCAAGAGGAACCATCTTTGAGGCCTTCCATGAAGAAAGTTCTACAAATGCTTG
AGGGAGTTGTTGAAGTTTCAATCCCTCCTGATCCATCTTCATTTATCAGCACAATTCAGT
AA
> gene:Csa4G005510
ATGGGTTTTTTCAAAATTGCAACTTTCCTCTTCGTCTTCTCCCTCCTCTTCATTCAAACA
AACACTGCCATTGTCAAATCCCAGAGCATCGATCAGATCAATCCAGGCTTTAGAGCTTC
TGCATCAGAATTCAACCACACAAATGGAGTTTTCCTCTTATCGAAAAGATCCGTTTTCG
CTCTTGGCTTCTACGCCGGTGCTAAAGACAACACATTCTCACTCGGAATCATACACATT
TTCAGTTCAAGAGTAATCTGGACAGCGAACAGAGATTCTCTGGTCAACGACTCTGCCT
TTTTTGTGTTCAATGAAACAGGGGATGCTTATTTGGATGTTTCCGGCCAGAATCAGACT
ACGGTTTGGTCGACTGAGACGGCTAATGAAGGCGTCGTTTCAATGCAGCTGTTGGATT
CTGGAAATTTGGTTTTAAAGAGTAAAAATGGAAGTTTTATTTGGCAGAGCTTCCATTTT
CCGACCGACACCCTTTTGCCCGGCCAGATTTTTTGGGAAGGATTGAAGCTCAAAAGTT
ATCCAAATGATAATGATCACTCGAATTTTTTAGAATTCAAACAAGGCGATTTGGTTCTTT
CCGCTGGTTATCAGAATCCCCAAATTTATTGGGCTCTGTCAAATGATAGCCGGAAAATC
CAGAGGGCCACCACCGGCGGCAGTGGGTATAGTCTGTTTGCGATTTTGGAGTCTAATTA
TTGGAATTTCTACGGCACGAACGGGGAATTGTTATGGAGTTTCAAAATTTTCTGGCAAT
TGAATAGGAAAGACAGATGGATCTCTGTTTTAAACACCGATGGAACGATTTCATTTCTG
AATTTGGAGAACAGGAAATCAGCAGAGCCGGAGCCAATTCGTATTCCGGCAGAAATTT
GTGGCGTTCCGGAGCCTTGCAATCCTCTTTTCATCTGTTACTTCGACAATCATTGCCAAT
GCCCTTCGACTGTTTTCGAAAAAAATTTCAATTGTAAACTCCCTTCAGTTCCCTGTAAT
GGCAGTTCTAATTCCACCGAGCTTCTGTATTTGGGGGAAAATCTTGATTATTTCGCTCTT
CGTTTCTCAACGCCTGCGTTCAACTCTGATTTGAGCTCTTGCAAAACAGCTTGCTCTAG
TAATTGCTCTTGCAATGTAATGTTCTATGAACCAGTTTCGAGGAATTGCTACTTCTTCAA
TGAAATCGGAAGTTTCCGGCGATCAGAAGGAGGTTCTGGTGGGTACATTTCTTATATGA
AAACAAACCTCCCGATCAATGGAAACAACAGCGAAACGAACCCAAGTCCAAACAGA
AGAAAGCACATTGTGCTTATGAGTTTACTTATGGCCGCAATGACTCTGGGGTTCATGGG
GTTGCTATGTTTTTTGTTCTACCGCCAGAAAATGAAGGAGTTGTTAAGCTCCATTGATG
AAGCGACAGAGGAAGATATATTTCTCAATGAGATCTCTGGTGGGCCAATCCGTTACAGC
TACCGGCAGCTCCGGAGAGCGACAAAGAATTTCTCCACAAAAATCGGGGATGGAGGA
TTCGGGTCGGTTTATTTGGGAAAGATGGGAGATGGGTCGAGATTGGCAGTGAAGAAAT
TGGAAAGAATTGGACAAGGAGGGAGAGAGTTCCGAGCAGAGGTGAGTTTGATAGGCG
GGATCCACCATGTGAATCTTGTGAAGCTGAAAGGGTTTTGTTCCGAAAGTCTCCATAG
GCTTCTGGTTTATGAGTATATGAGCAATGGATCCTTGGATAAGTGGATTTTCAACAAGA
AAGAGGATGATCTGTTTTTGGATTGGAGCACAAGATTTAACATTGCATTGGGTACAGGG

AGGGCGTTAGCATACCTGCATCAAGAATGCGAATCAAAGATAATCCACTGCGATATAAA
GCCAGAGAACATCCTTTTAGATGAAAACTTCACACCCAAACTCTCTGATTTTGGAATGG
CCAAATTAATGGACAAACAACACACCTCCATCTTCACACAGCTTCGTGGAACCAGAGG
CTACGTCGCTCCGGAATGGATCACAACCCTCGCCATTTCCGACAAGAGTGATGTCTACA
GCTACGGCATGCTTCTCCTCGAGATAATCGCCGGTAGGAAGAGCTACGACGCCGATTAT
CCGCCGGAGATGGCGCATCTCCCGTCCTACGCGACTAGGATGGTGGGGGAGCAAAAG
GGGTTTCGGGTGTTGGACTCAAGAGTGGCGGGGGAGGCAGAGGGTGATTGGCGGGTG
GAGGCGGCAGTGCAGGTGGCGGTGTGGTGTGTCCAGGAGGAGCCAAGTCTACGGCCG
CCGATGAGGAAGGTGGTGCAGATGCTAGAAGGGGTTAGTCCGGTGCCAATGCCTCCGT
GTACGGCGGAGATGGGGGCGAATTTCTGGTGGAGCAGTGATGGATTGGGAATGAAATT
GAATGGGTGTTATAGTGAAGTGAGGTTATCGGATGTTCGATTGTCGGGGCCTCGATGA
> gene:Csa4G289650
ATGGCTTCCCACTGCTTTCCATTCCCATCTCTTCTTCTTCTTCTTCTTTTTCTTCTTACCC
CTTCTTTTACCGTTGCTCAGGCAACAAGCCCAAATATAACTTTACGCAAATCTCTCACT
GCTCGTTCTAGCGATTCCTTCTGGTCCTCTGCTTCTGGTGCTTTTGCTTTCGGTTTTCGT
CAAGCTGTAGGGGGAGACTATTTGTTAGCAATTTGGTTCAACAAAATTGATGAAAAAA
CCGTCGTTTGGTCTGCTAATCGGGATAAATTGGCGCCGGGGGGTTCCACCGTTGTACTT
ACAACAAGTGGTCAACTCATCCTCAACAACCCTGCCGGCAAGCAAATCTGGTCTTCAA
CTTCTACGGCTCCTAATAAGTCTGTTTCCTCTGCGGTCCTTCTTGATAATGGAAATTTCA
TTCTCGCTGCTAACGATTCTGAAATTGTTTGGCAAAGCTTCGATGACCCAACCGATACG
ATTTTACCGTCACAGATTCTTAAGAAGGGAAACAAACTGGTTGCTTCTTATTCAGAAAC
GAATTACTCAAGTGGGAGATTTGAATTTTATATGCAAACTGATGGGAATCTTTTGCTTTA
TATAAGAAATTTTCCTTACGATGCAATAAGTAACTATTACTGGTCAACTGACACTGTGAA
CTTCGGCTTTCAAGTCGTCTTCAACCTCTCTGGTTCTATTGTTCTCATCGCAGAGAATAA
AACCATTCTCAACACTCTCTCATCAAATAATCCCACGGCTCAAACTTTCTACCAGCGAG
CAATTTTGGATCATGATGGGGTTTTCAGACATTATATTTACCCAAGGGGTGGCACTGGA
AGAAATTCATCTTGGCCTAAAGCTTGGTCGATATCGAAATCCATTCCTTCCAACATCTGT
ATGACAATTGGTCAAAGTTCGGACGGTGGAGTTTGTGGGTTCAATAGCTACTGCAAGC
TTGGGGATGATCAAAAGCCATTTTGCAGTTGTCCAGAAGGGTATGTCTTGTTTGATCCA
AATGATGTAACACAAAGTTGTAAACCCAATTTTGTTCCTCAAAGCTGTGCGTTTCCTGA
ACTTGATGACTTTGATTTTGTTTCTTTGGACAATTCGGATTGGCCGCAATCTGATTACGG
TGATTACGGCCATAACATACCAGTCAATGAAGATTGGTGCAGAAACGAATGTTTAAACG
ATTGTTTTTGTGTAGCAGCCACTTTTAGAGATGGGAATTGTTGGAAGAAGAAGTTCCCA
CTTTCATTTGGGAGAATGGATTACAGCGTGGGAGGAAAAGCTCTTATCAAAGTCAGGA
GACGCAATTCAACTCTGCAATCTCGAAATCTTGACAAGAATTGCAATAACGAAACTAA
GATAATCATTGGATCAATTTTGTTAGGAAGCTTATTCCTTAATATCCTCTTATTGCTACTC
ACTTTATTGATCGGCTGTCGATTCAGCAAAAGGAAATTAAAGTTTAATGGAGGAGACCC
TTTCATTTTAGAGCAACTGGGAAGTGGTGCATTTGCTACTGTTTATAAAGGGACGACTC
TTGGTTCTGTTGATGACAACAACTTGGTGGCAGTTAAAAAGTTGGAGAATATAGTGAAT
GAAGGATCAGGAGAGAATGAATTTAAAGCCGAAGTAAGTGCTATTGCTCGAACAAACC
ATAAGAATTTGGTTAAGTTAGTTGGTTTTTGTAACGAAGGAGAACACAGAATGTTGGTT
TATGAGTTCATGGAAAACGGGTCTCTTGCAAATTTTGTTTTTAAGCCTTCAAAACCTAC
CTGGTATACAAGAATTCAGCTTGTTTTAGGAATTGCAAGAGGCTTTGGGATAATGTTATT
GGAGATGATCTGTTGTAGAAAGAATTTTGAAATGGAAACAGAAGATGAAGATGAAAG

GATACTGAGTGATTGGGCTTATGATTGTATGAATGAAGGGAAAATGGAGAAGTTGATAA
GAGAAGATGAAGAAGGAAGGAGTGATATGAAGAGAGTGGAGAGATTTGTTAAGATTG
GAATATGGTGTATTCAGGAGGATCCATCGCTAAGGCCATCCATGAAGAAAGTGATACAA
ATGCTTGAAGGTGTAGTTGAAGTTTCAACTCCTCCCGATCCATCTTCATTTATCAGTGCT
ATTAATTAA
>gene:Csa7G446780
ATGTTTCTATTTCCTCAACTCTTAATTCTATTCTGCTGTTTCTTTGTAGCGCTTTTCATGG
CCAAGTTTTCCCATGGCCACACCACACTAGCAAATGATGTTCTTGCTCAAGGCCAACA
CCTATCAATTGCATTTTACATTCCTCCAAGTTCTAATTCCATTTATTTGGGAATTTCCGAC
AACACAAATGACCAGAAACCAATCTGGATAGCCAATCGAAACTCCCCATTTCCCAACA
ATTCTGCTTCGATCAGCCTCACAATTGACGTCAACGGCAGCTTGAAAATCCAAAGTGG
GAATTGTTCCTTTTCACTTTTCAATGGCGGACAACCGACCACCAGCAGCGCCATATTAC
AAGACAACGGCAACTTTGTACTACGAGAGCTGAACAGAGATGGGTCAGTAAAGCAGA
TTGTGTGGCAGAGTTTTGATCATCCAACAGATACTCTGCTTCCCGGAATGAAAATTGGG
ATCAATCACAAAACTAACTCCACTTGGTCATTAATATCATGGCGAAACTATAAATCTCCG
AAGCCGGGAGGTTTGAGTCTGGGAATGAATCCGAACAATACATATGAGTTAGTGGTTT
GTGTCCGAGGAGAACTGCTTTGGAGGACTGGGAATTGGAAAGAGGGTTCGTTCGAAT
TCTTGGAAAAAGATAAAGGATTCAATTTCGTTCGAGTTTCGAATGAGAACGAAACGTA
TTTCATTTACTACGCCCGGGAACCCAATGGATATTCTCTGTATAGAAATTCATATTATCAC
GGAGAGTCAGGGGAACTTATTTTATCCCAAATCAGATTAGAAAATAATGGGAATGTTAG
AATCAACAATGAAATATATGACTCTCCCTGCCTCTTGACTTCGAATGAGATCCGTGGTG
CTTGTGTGTGGAGAGAGCTGGACAAAATACCGGAGTGCAGGAATAAGTTGTCTCATGG
CTATGGTCCGTACATTTCGCAGATTAATGGTTACGAGTTGGAGAGGATTAATGGTTCTGA
TTATTACTACAAACTAAGTGGAAACTTAACCATGTTTGAGTGCCGCAGTATTTGCATTAA
TGATTGCGATTGCATCGCTTTTGGGATTCCAGCGTACGAGAGTGACTCCGGCTGTGAGT
TTTGGAAGTCAGGTGCCAACTTTATTCCAGAGAACGACAGTTTACAAATGCTTTGGTC
ACTGGACACAGATAGCGAGTTTCTAGACACAGATCACGAGTTTTCAAACACAAATGAC
GAGTCTCCAAATGGAAAATGGAAGGTTTGGGTGCAAATAACTGTGGCTCTAACCCTAC
CTGCAACTTTTCTCCTACTCTGTTTTATTATATACACCAAATGGAGAACACAAATATTCA
AAGCTATCGGAAAAGTAAAGAAAGGTTTTCTTCGGGGGATGGGGATGATATCCGAATG
TTACAACATATTAAGAATAATGATCATACAAATTAGAGATGGAAAAAAGAACCCCGAAT
TGCAATTTTTTGACTTCGAAACCATACTCTCTGCTACGAATAATTTTGGTGAAGAGTATT
CGGAGAAGAAGTTAATATTTGATTGGGAAAAACGTTTGCACGTTGTCCAAGGAATAGT
TCAAGGACTACTCTACCTTCACTACTACTCAAGAGTACGAATAATTCATCGAGATTTAA
AAGTTAGCAACATTTTACTGGATGATGAAATGAATGCAAAAATATCGGACTTTGGTATG
GCTAGAGTCTTTAAGCCATCTGACAACGAAGCAAACACAAGTCGAGTGGTTGGTACAC
ATCAAAAGAACTACCACAATTATGACACAGAACGACCACTTAACCTCATAGGGTATGTA
ATTAAAAATGATAATGTTTGTATTTTTCAGGCATGGGAATTGTGGGTGAATGGGAGAGG
AGAAGAGCTGATTGACTTGGGTTTATGCAATTCTGATGATCAAAAAGCAAAGGCCCTA
AGGTGCATTCATGTTAGTCTTCTATGTGTTCAACAAATCCCAGGGAATAGGCCGACGAT
GTTGGATATTTATTTCATGATCAACAATGATTCCGCTCAACTTCCATCTCCCAAACAACC
TGCTTTTTTTATTGCTCAAAGCCCAAGTTCCTCCCAACGGGAAATAGAAGAGGTGGATA
GTGAAACTCATACGACCCATAGAAGCAATTTATTCATTGAATTCTATGACACTCTCAAC
GATGGTTGCAAGTCTTCTATGGCGGACATACTACATCAAGGCCAAGAATTAACAACTGG

GTCTCAGTTAATTCCAGCTACCGTCATCTTTGTTCTAGGCTTTAACTATTATCCACCAAAT
ACCAATACCATGACCCTCCCCATCGACACCACCAGTGCGATGTTACAAGACGACGGTG
ATTTCGTATGGCGAGAGCTGAAACCGAGATGGATCAGTAAAGCAAATTCTGTGGCAGA
GCTTTGA
> gene:Csa1G071150
ATGGTTCTGTTTCCAAGAAAATCATTTGCAATTGACAGCATAAAAGCAGGGGAATCCAT
CAATGGCAGTAACCAAATATTAGTTTCAGCTCAACAGAAGTTTGTGCTGGGAATTTTCA
ATCCTAAAGACTCCATATTTCACTACTTGGGAATATGGTACATGAACATCCCACAAACG
GTTGTGTGGGTAACAAACAGAGACAACCTACTTTTAAATTCCTCTGTCATACTAGCATT
CAAGGGAGGAAACCTGGTTCTTCAGAATGAAAGAGAGGGAATTATATGGTCTTCCATT
TCTTCAGAATTTGTTAAGGTACCAGTTGCTCAACTACTAGATAATGGTAATTTGGTTATA
AGAGAATCCGGGTCTGAAAATTATGTGTGGCAGAGTTTTGATTACCCTTCTGATACTCT
GTTACCGGGCATGAAACTCGGTTGGGACTCAAAAACCGGTATGAAGTGGAAGTTAACG
TCATGGAAGAGCTTGAACGATCCGTCATCTGGGGATTTCACTTTTGGTATGGACCCTGA
TGGGCTTCCCCAGTTTGAAACTCGCAGAGGAAACATCACAACATACAGGGATGGCCCA
TGGTTTGGCAGTAGGTTTAGTCGCAGTTCTTTTTTTAGTGAAGTGGAAATTACTTCACC
ACAGTTCGATTATAATGCGGAAGGAGCATTCTTTTCATACGAGTCTGTGAACAATCTCA
CTGTAATATATGCACTGAACGCACAAGGCTACTTTCAAGAATTGTATTGGAAAGATGAT
GCAAATGATTGGTTCTCTTTGAATGAATTACCAGGAGATGGCTGTGATGACTACGGACA
CTGTGGAAATTTTGGTATTTGTACATTTTCCTTCATACCCCTCTGTGATTGCGTTCATGG
GCATCGACCAAAATCTCCAGATGATTGGGGAAAGCATAACTGGTCAGGTGGCTGCGTT
ATAAGAGACAATCGAACCTGCAAAAATGGAGAGGGGTTTAAAAGAATCAGCAATGTG
AAATTGCCAGATTCTTCATGGGATTTGGTGAATGTTAATCCGAGCATTCATGACTGCGA
AGCGGCATGCTTGAGTAATTGCTCTTGCTTGGCCTATGGAATAATGGAGCTTCCCACTG
GTGGCAATGGCTGCATCACATGGTTTAAGAAGTTGGTGGATATTAGGATCTTTCCTGATT
ATGGACAGGATATCTATGTGAGATTAGCTGCTTCAGAATTAGTTGTAATTGCAGATCCTT
CAGAATCAGAATCGCCTAAGAGGAAGCTTATAGTTGGCCTGAGTGTGTCTGTTGCTTCA
CTGATAAGCTTCTTGATTTTTTTTGCTTGCTTTATCTATTGGCGTAGGAGGGCTGAGGGT
AATGAGGTTGAGGCTCAAGAGGGTGACGTTGAGTCGCCACTCTATGATTTTACGAAGA
TTGAGACTGCCACAAATTATTTTTCTTTTTCAAATAAGATTGGCGAAGGTGGCTTTGGT
CCTGTGTACAAAGGAATGCTTCCATGTGGACAAGAAATTGCAGTAAAAAGACTGGCAG
AGGATGACAAGAAGCGATCTTTACTTGGTTGGAAAAAGAGGCTGGATATTATAATTGG
AATAGCTCGAGGTCTTCTCTATCTCCACCGAGACTCGAGGCTTATAATTATACATAGAGA
TCTCAAAGTGAGTAACATCTTACTTGATAATGAAATGAATCCAAAAATTACGGACTTCG
GTATGGCGCGTATGTTTGGTGAAGACCAGGCTATGACACAAACGGAAAGAGTTGTTGG
GACCTAG
> gene:Csa_4G289620
ATGACGTCCCAACAAAAACCAACTTGGAAAGTGAAAAGAAATTTATATACCAACTGTT
ATCTCAGTATCAACGCCATTCATCTTCACAAAAGCCTTACCTTTCTTCAGCTTATGGCTT
TTAAAATCACACCATCTTACTTCCTCTTTCCCCCATTTCTTCATTCTCTTCTTCTTCTTCT
TCTTGTTTTACCAACTTGTTCCTTTTCTCAGCTTTTTAAAAATGTAACTCTGGGTTCATC
TCTCACAGCAACTCAACTAAATGATCACCACAACTATTGGGTCTCCCAATCTGGTGATT
TTGCTTTTGGGTTTCTACCCTTGGGAAGTCAAGGTTTCTTGTTGGCCATTTGGTTCTAC
AAAATTGATGAAAAAACTGTGGTTTGGTCAGCTAATCGTGATAAATTGGTTTCTAAAGG

GTCCACAGTTCAATTTACTAGTGCCGGCCAACTTGTGCTTAATGATCCTGGAGGCAACC
AAATATGGACGGCCACTGCAAGTTCCTCTGGAAATACTAATAGATCCGTTTCTTATGCTG
CGATGCTTGACAGTGGAAACTTTGTTTTGGCTGCGACTGATTCTGAAATTTTGTGGCAA
AGATTTGATGTGCCTACTGATACAATTTTACCATCACAAACTTTGAATATGGGTGGAGCT
CTTGTTGCTCGTTATTCAGAAACTAATTGTAAGAGTGGAAGGTTTCAACTTTTGATGCA
AACTGATGGGGATCTTGTGCTTTTCCCCCATCCTCTTGAAAAAACAAACATATCTTACT
GGGCAAGTAACACTACTCGCTCTGGCTTTCAGCTTGTGTTCAGCCTTGCTGGTTCCATT
TATGTGATTGCAAAGAATAATACCATTCTCACAACTGTGGTACCCAATACCCTTTCACCA
CAAAATTATTACCTCCGGGCGATTCTTGAGCACGATGCTGTTTTTAGATTGTATGTTTAT
CCGAAAGTGACAAGTAATTCAACAATGCCTAAAGCTTGGACTCAAGTATCAGACCCTG
TAAACATCTGTATTATGGTCAGTAATGGTACAGGAAGTGGAGTTTGTGGATTTAATAGCT
ATTGTAAGGTTGCTCAGAGGTGCGATAAATCCTTTATTGAAACTGATGACTTCGAATTT
GTTGCTATGGAAAATACGAATTGGCCTCACGGGGATTACGCCAATTTCAACCCAGTAAG
TGAGGACTGGTGTAAAAACGAATGTTTGAATGATTGTTTTTGTGCGCTTGCTACTTTCA
GAAATGAATCAAAGGAACAAAACAACAATCATTGTATCAGTTCTAGTAGGAAGCTCTA
TTTTCCTAAACTTTCTCTAAACTTCATCTTATTCCTTCTCACTTTATTCATTTGTTACCATT
TCAGGAAAAGGAAATCGGATGTGGTTGAAAAAGACCCTTCCATTTTAGGTGTACTGAA
TATCAGGATCTTTAGCTACGAAGAGCTCAACAATGCCACAGGTGGATTCATCCAACACT
TGGGGCGTGGCTCTTTTGCTACTGTTTATAAAGGGATTATTGACTCTGACAACAACAAT
AACTTGGTAGCTATTAAAAAGTTCGATAATGTGGTTCCAGATGGAGAACAAGAATTTAA
GGCTGAAGTGATTGCTATTGCTCGAACAAACCACAAGAACTTAGTTCGATTGCTTGGC
TTTTGCAACGAAGGAGAACACAGAATGATGGTGTATGAGTTCATGCCTAATGGGTCTCT
TGCAGATTTCCTTTTTGGGACTTCGAAACCAAATTGGCATAGCAGAATTCGAATTATTTT
AGAGACGGCCAGAGGACTATGTTATCTACACGAAGGGTGTAGTACTCAAACCATTCATT
GTGATATTAAGCCTCAGAACATCCTTCTGGACGAGTCGTTTTCCGCAAGGATTGCAGAC
TTGGGGTTGGCCAAACTTTTGAAAAAAGATGGAGCTCGGACCACCCCAATGACAATG
ACAATGACAAATGGAGAAAGCAAAGGATATGTAGCTCCAGAGTGGTTCAGAGGCCTC
CCCATCACAGTAAAGGTGGATGTTTATAGTTTTGGGGTAGTGTTGTTGGAGACTATATGT
TGTAGAAGGAGTTTGGAGGAGAAAGAAGAGAATGAAAAACAAAAGGTGTTGAGAGA
TTGGGGTTATGAGTGCTTGAAAGAGATGAAAGTGGAGATGTTGGTAGAGAAAGATGA
AGAAGCAAAGATGGAGTTGAAAAAAGTGAAGAAGTTTGTGATGATAGCAATATGGTG
CATTCAAGAGGAACCATCTCTAAGGCCAAGCATGAAGAAAGTGTTACAGATGATGGAG
GGTGGCATTGAAGTTTCCTTTCCTCCTCATCCATCTTCCTTCATTAGTTCAATTTCTTAG
>gene:Csa_7G045520
ATGGAGGAAGATAGAAATAGTGGAGAGGGAACATGTTTGTTGCATAACAGAAGTAGTG
AAATGGTGGCCAAAAGAAGTAGTGTCAAGAAATTGGTCACCATCAGCTGGTTTGCAGA
GCATTTGATGTCTTTTTTCCATTTGTACTCATTTGTGTTTTTAATTTTCGTTGTTAATTGCT
TTGCTAAAGATACCTTAGAGTTTAAGAGTTGCATAAGTCATGGGAGTGGGGATACTCTT
GTATCGGCAGGGTCGAGATTCGAACTTGGATTCTTTCAGCCATATGGCAGCTCTCATAG
CAGAAGATACTTGGGAATATGGTATTACAAATCAAATCCAATTACGGTTGTTTGGGTTG
CCAATCGAGACAGACCACTTCCTAGTTCAGATGGTGTCTTGAAGATCGAGGATGATGG
CAACCTCAAAGTATATGATGGAAATCAGAATCTTTATTGGTCAACAAACATTGGAAGTT
CTGTACCTGATCAAAGGACCCTGAAACTAATGGATAATGGAAATCTGGTTTTGAGCTAT
GTAGATCAAGAAGACTTGTCTGAGCACATCCTCTGGCAAAGTTTCGATTATCCAACTGA

TACATTTCTTCCTGGCATGTTAATGGACGATAACTTGGTGCTGGCTTCATGGAAGAGCTA
TGATGACCCGGCCCAAGGGAACTTCACTTTTCAGTTAGATCAGGACGGAGGTCAATAT
GTCATTTGGAAAAGATCAGTTAAATTCTGGAAAAGTGGAGTTTCAGGTAAGTTTATTAC
CACTGATAAGATGCCTGCTGCATTGTTATACCTTTTGTCAAATTTCTCATCGAAAACTGT
CCCAAACTTCTCTGTGCCACATCTCACATCATCATTGTATATTGATACAAGGCTAGTATT
GAACAGCTCAGGTCAGCTTCATTACCTAAACTGGGAAGATCACAAAGTCTGGTCTCAG
ATTTGGGTGGAGCCGAGAGATAGATGCAGTGTGTATAATGCGTGTGGAGATTTTGCTAG
CTGTAATAGTGAGTGTGGTATGGCTTGCAAATGCCTGCCTGGTTTTGAGCCTACCTCTC
CAGGGAGTTGGAATATTGGAGATTATTCAGGTGGATGCATTAGGAAATCACCAATATGC
AGTGTGGATGCTGACAGTGACACTTTTTTGAGTCTAAAGATGATGAAAGCTGGAAACC
CGGACTTCCAGTTTAATGCAAAGGATGACTTCGATTGCAAATTGGAGTGCCTTAACAA
CTGCCAGTGTCAGGCTTACTCGTATTTGGAAGCTAATATCACAAGGCAGAGTGGGAATT
ATAATTCTGCTTGTTGGATCTGGTCTGGAGATCTCAACAATCTTCAGGATGAGTTTGATG
ATGGTCGTGACCTCAATGTTCGAGTAGCAGTTAGAGACCTAGAATCAACTGCCAGAAA
CTGTGGAACATGTGGAACAAACCTCATTCCTTATCCACTTAGCACAGGACCTAAATGCG
GTGACCCCATGTACTTCAATTTTAACTGCAACTTGGCTTCTGGCCAGGTAAACTTTGAA
GCAGCAGGTGGAACATACAAGGTTAAATTCATTGATTCAGAAGCACGAAAATTTTACAT
CCAAACCAAGGAACCGGGTGATTGTGGTGATAAAAACTGGATAACCAAAGCCCTTCA
GCTGAACCAGTCATCTCCTTTCCGTGTAACCAGCTGGTGCAACTTTAAGGAGACCAAT
CTCGAGGAGAATTTTTCTCTGAAAACTAGCAATGAAGTTGAAATCAGTTGGGAGCCTC
CACTAGAGCCAATTTGTTCTTCAACTACGGACTGCAAAGACTGGCCTTATTCAACTTGC
AACATGAGTAAAGATGGAAATAAAAGATGCCTTTGCATAACAGATTTTCATTGGAATGG
CTGGATCTTGAATTGCACTACAGATCACAACAAAGGAAAGGATGGAAAAGGCAAAAC
GACCTTTTCTGTGATTATTGTTGCAACATCCTTATGTATGGTCCTTCTGATGATTCTCTCA
TGTACCGTATTTTACATTTACTTCTCCAAAAGTGGGCTCATTGAAAGACAAGAAAGCAG
GGGAAACAGCCAAAAAGATTTAATGCTTCACTTGTACGATAATGAGAGACGTGTCAAA
GACCTGATTGAATCCGGTCGATTCAAGGAAGATGATACAAATGGAATAGACATTCCATT
TTTTGATTTGGAAACCATTCTAGTTGCAACAGATAACTTCTCAAATGCAAACAAGCTTG
GCCAGGGAGGCTTTGGGCCAGTTTACAAGATGAGTGTGGCATTGGATTGGGACATGCG
CTTCAACGTCATTTTGGGAATTGCTCGTGGCCTTCTTTATTTACACCAAGATTCGAGGTT
GAGAATTATCCATAGAGATTTGAAGACGAGCAATATTCTTCTAGATGAGGAGATGAATC
CCAAAATATCCGACTTTGGCTTGGCGAGGATATTTGGAGGGAAAGAAACTGCCACAAA
TACTAAAAGGGTGGTAGGCACTTAG
> gene:Csa4G288620
ATGGCTTTGAAAATCACATCATCTTACTTTCTCTTTCCCCCATTTCTTCATTCTCTTCTTC
TTCTTTTTATTCTTCTTGTTCTACCAACTTGTTCCTTTTCTCAGCTTTTTAAAAATGTAAC
TCTGGGTTCATCTCTCACAGCAACTCAACTAAATGATCACCACAACTATTGGGTCTCCC
AATCTGGTGATTTTGCTTTTGGGTTTCTACCCTTGGGAACTAATACTTTTTTGTTGGCCA
TTTGGTTCGACAGAATTGATGAAAAAACTGTGCTTTGGTCAGCTAATCGTGATAATTTG
GTACCTAAAGGTTCCACATTTCAATTTACTAATGGCGGTCAACTTGTGCTTAATGATCCT
GGAGGCAATCAAATATGGACGGCAACTGTAAGTTCCTCTGGAAATTCTAATCGATCTGT
TTCCTATGCTGCGATGCTTGATAGTGGAAACTTTGTGTTGGCTGCGGCTGATTCTGAAA
TTTTGTGGCAAAGCTTTGATGTGCCTACTGATACAATTTTACCATCACAAACTTTGAATA
TGGGTGGAACTCTTGTTGCTCGTTATTCAGAATCCACTTATAAGAGTGGAAGGTTTCAA

CTTGTGATGCAAACTGATGGGAATCTTGTGATTTACCCAAGAGCATTCCCTTTGGATAA
AGCAAGTAACGCTTACTGGGCAAGTAACACTATGGGCTCTGGCTTCCAACTTGTGTTC
AACCTCTCTGGTTCCGTTGATGTCATTGCAAACAATAATACCGTTCTCTCAACTGTGCTA
TCAACCACCCTTTCGCCACGAAATTTTTACCTACGGGCGATTCTCGAGCATAATGGGAT
TTTTGGACTGTATGCTTACCCGAAGCCCACACATAGTTCGTCTATGCCGAGAGCTTGGT
CTCAAGTGTCAGACTCTATAAACATTTGTATCTTGGTGCAGACTGGTTGGGGATCTGGA
GTGTGTGGATTTAATAGTTACTGTAGGCTTGGTGATGATCAGAGGCCATTTTGTTCTTGT
CCTCCTGGCTATATCTTGCTTGATCCAAATGATGAGATCAAGGGTTGTATACCTAACTTT
GTTGCTCAGAGTTGCGATCAATCCTTTCATGAGACTGATAACTTTGAATTTGTTGCTATG
GAAAATACTAATTGGCCTAGTGCTAATTATGGCTATTTCAAAGTAGTAAGTGAGGAATG
GTGCAGAAACGAATGTTTGAACGATTGTTTTTGTGCGGTTGCCTTCTTTAGAAATGGTG
AATGTTGGAAGAAGCGGTTCCCTCTAGGTGATGGACGAATGGATCCTAGTGTTGGTGG
AAGAGCACTTCTCAAAGTTAGGAAACAAAACTCTTCTTTCCAACCAAATGATCTTGTC
CATAAACCTACAATAGTTGTCGTTGGATCAGTTCTATTAGGAAGCTCTGTATTTCTCAAC
TTCTTCTTATTCCTTCTCACTTTATTCATTGGCTACCGACTCAAGAAAAGGAAATCAAAG
CCTGTTCAACGAGATCCATCCATTTTAGATGTGAATTTGAGGATCTTTAGCTATGAAGAG
CTCAACAAGGCCACAAGTGGATTCATCCACCAGTTGGGGCGTGGCTCTTTTGCTACTG
TTTATAAAGGGACTATTGACTCTGAGGACAACAATAACTTGGTTGCTGTTAAAAAGTTG
GACAATTTAGTGCAAGAGGGAGACCAGGAATTTAAAGCTGAAGAGAGCACAGAATGT
TGGTGTATGAATTCATGCATAATGGGTCTCTTGCAGATTTCCTTTTTGGGACTTCGAAAC
CAAATTGATCAAACTAGAACTCTGACAGCGATTAGAGGAACCAAAGGATATGTGGCTC
CAGAGTGGTTTAGAAGTCTTCCCATTACAGTGAAGGTGGATGTTTACAGTTTTGGGATA
ATAATGTTGGAGATTATATGTTGTAGAAGGAGTTATGAGAAGAAAGTAGAGGATGAAG
AACAAATGGTGCTCACAGATTGGGCTTATGACTGCTTCAAAGACATGAAAGTGGAGAT
GTTGGTAGAAAACGATGAGGAGGCAAAGATGGACTTGAAGAGGGTGAAGAAGTTTGT
GATGATAGCAATTTGGTGCATTCAAGAAGAACCATCCCTAAGGCCTACCATGAAGAAA
GTTCTACAGATGCTGGAAGGTGCTATTGAAGTCTCTTTTCCACCTGATCCATGTTCCTTT
ACGAGTTCTAGTACAATTATCTGA

L-Type

> gene:Csa5G648630

ATGGCTCCCTTAACTTTTCTTATACTGTTCTTCACTGTGCCTCCTTACTTTTTTGCAGACT
CTAAATTTTTATACAATGGATTTCATGAAGGAAAAGGATTGAATCTTGATGGGGCGGCT
ATTGTTAAGCCATCTGGGGCGTTATGCCTCACCAGCAATTCACAAAATGTAGTTGGCCA
TGCGTTTTATCCTGATCCAGTTATGTTGTTTGATCCACGCTCTCCTTCAAATACTTCATCT
TTCAGCACAACTTTTGTGTTTGCTATTGATCCTTCAATCCCCGGTCACGGCGGCCACGG
CTTGGCCTTCACCTTGGCTCCATCAACCAGGTTTGATGAAGCTGAAAGTGGACATTACC
TTGGATTATTCAACCCTCTCAACGATGGAAATCCATCCAACCATATATTCGCTGTAGAAT
TCGACACTGTTAAGGGGCATGGTGGAGTAACGAATTCTAGAGGCAACCATATTGGGATT
AACATCAATGGCATTTCCTCAGTTAAATCTCAATTGGCTGCTTCTAGCTATTATGTTGAT
GATACGGTTTGGAAAGAAATCCAAATTGATTCTGGTGATCCAATCGTTGCTTGGATTGA
TTATGATGGTCGTAGTAAAAATTTGAGCGTCACTATTGGTCTTTTGGAGCTAAAGCCAG
AGAAGCCACTCATTTTATGTCATATTGATCTAACTTCTGTTATGAAGAATCAAATGTTCG
TGGGGTTCGCTGCATCGACCGGTATCGAAACAAGCGCTCATTACATTTTGGGATGGAGC
TTTGCAGTGGATGCAACGGCACGGCAACTGAAGTACTCTCAACTTCCCAACAGACCAA
ATGAGCAAAATATTTCTTCTTCCTCCAATAACAATTCCCAGTTGAAATCTGTTTTAGCAG
TGTCATCTATTATAGTCCTTATGGCAATTGTTATCTTAACCTTCTTGTTTATAAGAATGAA
GAAAGCAGAGAGCTTGGAGGATTGGGAAAAGGATTGCCCTCATAGATTCAACTTCAAG
GATATTTACACAGCAACAAATGGATTTAATGACAGCGCCCAAATTGGAATTGGGGGATT
CGGGTCAGTATATAAAGGCAAGTTAAGTTCAACAGGTGCTGAGATTGCTGTGAAGAGA
GTCAAACGGGACTCAAGCCAAGGAATGAAGGAATTTGCTGCGGAAATCGAAAGCTTA
GGACGATTAAGACACAAGAACTTGGTTAATCTTCAAGGATGGTGCAAGAAACAGAAC
GATCTTCTAATAGTTTACGATTATATTCCAAATGGAAGTCTTCATTCTCTTCTCCACACTT
CAAAACAGAGCGTGATATTGAAGTGGGAACAAAGATTCAACATCCTCAAAGGCATTGC
TGCAGGATTACTATATCTTCATGAAGATTGGGAGCAAGTAGTGATCCACCGAGATGTAA
AGCCGAGCAATGTTCTAATTGACGCCGACATGAATGCACGATTGAGTGATTTCGGATTG
TCAAGACAATACGACCACAACGAAATGTCACACACAACTCGAGTCGTGGGGACAATA
GGCTACATACCCCCAGAGTTGTTTCGCACGGGGAAGGCGTCAAAGAGCGCAGATGTGT
TTGCATATGGAGTTTTGCTTCTGGAAGTGGCGTGTGGAAGAAAGCCTCTAGGATCAAA
CCAATTCATATTGATGGATTGGGTGATGGAATGGTATGAAACAGGGGACATTCTTCATG
TGGCTGATCCAAAATTGGATTCCATTTATAAGGTAGAAGAGATGGAGATGGTATTACAA
CTTGGTCTTCTTTGTACTCATTGGAAACAAGAAGCTCGACCCTCAATGAGACAAGTTAT
GAGGTTCCTTAATAGAGAAGACCCACTTCCAGCATCCGATGCATGGACTAATTCTCAAA
GTATCTTTGAATCCAGTTCAAGATTGACAATGACTGATCGTTCTTCATCCATGTCTGTTG
GTCCGATCTCCTCTGCCTCCATAAACGAAGGAAGATAG

> gene:Csa7G067430

ATGTCTCACATGGAGGTCTCAATTCTCACCTCTCCAAGCTGTCTTCTACTCATCTTATGC
TACTCTAACATCTTTTGGAGTCTAACCAACCCAGGTTCAGTCTCAGCCATCATTTCTTAT
GAGAACTTAACATTCAACCTCACTGATTTCGGCCCAAATGACCACGACATTCATTATGA
AGGAGATACATACCCTTCCAACAATGTCATTCAGCTTACCATGAACCAGAGAGACATGC
CTCTCAATGGAAGTGTTGGTCGTGCAACCTACAGAGATCCTTTCCATCTTTGGGAGAGT
GGCCACAGGAATCTTGCAGATTTCACCACCCAGTTCACCTTCACAATTGACTCACAGC

ACAGTCGCACTTATGGCGACGGCTTTGCCTTCTTCATTGCCCCTGTGGAATCAAGGCTT
CCACCTCACTCAGGAGGTGGCAACTTTGGTCTCTTGAGTTCCAACAAGAGTGATCCAG
ATGTGGTTCCAACAGCCAACTTTGTTGCTGTCGAGTTTGATACCTACACAAATGCATGG
GATCAATCGGAGAATCACGTCGGCGTTGATGTTGACAATGTTAAATCTCTAAGCTCTAC
TTCATGGTGGTGGAGTGACATAGAAAATGGTGGGAAAGTTAAAGCAGCAATAAGTTAC
AACTCAAGCTACCATAATTTGACTGTTTTCTTGGTTGATGAAAGAGATTCTGAAGTTTC
TCCAACAAACTCTTCTACCTTCACATTCAATATTGATTTGAGAGAGCATTTACCAGAATG
GGTAACAATTGGCTTTTCAGGATCAACCGGATCTTTCTTCGAGATTCATACAATTAGCTC
ATGGAGTTTCAGCTCTATATTACAAGTTGAAGTCAATGTTACTACCACAACTGAACCAG
CTAGCTCTCCTGTTAACTCCAAGAAAGGAATCAACATGAAATGGTTCGGGATAATCTTC
ACCGTGGCACTTTCACTCTTTCTAATTTTGGGGTTCGTTTGGTTTGGGGTATGGATGAA
GAGAACTAGCAGAAGAAAAAGCATGAGAAGGAACCAAGAGGAAGATTTTGAAAACG
AAACAGGGCCAAGGAAGATTTCATATAAAGACCTATTAGCAGCAACCAACAAGTTCAG
TGATGAAAATGTGTTGGGACAAGGAGGCTTTGGTAAAGTATACAGAGGGTTCCTAGAC
AACAAGGAACTTGATGTGGCAGTGAAGAGAATCACCCCAAACAACCTCCATCAAGGG
TCAAGAGAATTCGCATCAGAAGTGAAAACAATTAGCAAATTAAGACACAAGAACTTAG
TAGAGCTAATTGGGTGGTGCTGCTGCAGTAAAGACCAAGAATATCTCATAGTCTACAAA
TTCATGCCTAACAAAAGCTTAGACTTCCACCTCTTTCAACAAAATAACCTCCTAACATG
GGATCACAGATACAAAATCGCAATAGGTTTAGCCTTAGCACTACATTACCTACAAGAAG
AACAAGATCCATACATTCTACACAGAGACATAAAATCAAGCAACATCCTATTAGACGCC
GAATTCAACGCGAAACTCGGCGACTTCGGACTCGCTAAGCTCGTTGATCATGGAAAAC
AATCAATAACCACCATACTGCGAGGAACAGAAGGTTATGTAGCACCAGAGTACCTGGA
AAGCAGCGTGGCAAGTAAAGAATCCGACATCTACAGCTTCGGCATAGTATGCTTGGAA
ATCGCCTGCGGAAAACAAGCCCTAGGTGAAGCGAGAGAAGACGGAAAGAGACGTTT
GATAAAACTGGTTGAATGGGTTTGGGATTACTACCGAAGGAGTGTGGAGGAGGCTGCG
GACCCGAAATTGCGGCAAAATTTCAAGAGAGAAGAGATGAAACAACTGTTGATCGTG
GGACTTGCTTGTGCTCAGCCGGATTTCCGTGTGAGACCTTCCATCAAACAGGTGATCG
ACATGCTGAACTTCAAATCACCATTGCCGAATCTACCACTGGAATATCCAGGATTGTCG
AGATCGGCAGTGTTTTTGAGTGCGGAAATGGAATCGTTACGAACATCGTCGTGTTTACA
AAGCGGAAATCTTCATGGAAAAAGTATTTCTTCCAAGAATTCCACTGCTTCTTCCACTT
TTTCGAATATGGGCTAA
> gene:Csa3G730920
ATGGCTATTGCAGCTCATCTGGCTCTTCTTTCTGTGTTTATCTTTTTCGAGGCTCATGCTT
TGCCGTCTTCTTTTATCTACCCTGGATTCAACAATACAAGCCTTGATCGTGAGGGGGCT
TCTGTTGTGAAGCCATACGGTGCGTTGAGGCTCACCAATATATCGCAAAATGTTATCGG
CCATGCATTCCATCCAACTTCATTCCGGATGTTTGAACAAAGTTCTGACTCGTCTCCGA
ATGTTTTGTCCTTCAGCACAACTTTTGTCTTTGCAATTGAACCTTCGAGCCCTGGGCAA
GGCGGCTATGGCTTAGCCTTTGCCATAGCTCCATCAACCAAATTTTCAGGTGCAGGAAG
TGGGCACTATCTTGGATTGTTCAACTCTTCCAACAACGGTAACCCTTCAAACCATATATT
TGCTATTGAATTTGACACTGTAAATGGGCATGGTGAAGAAAGGAACACCAAGGGAAAC
CATGTTGGGATCGACATCAATGACATTTCATCTGTTACATCCAAACCTGCTTCTTATTCA
GACTATGGCGAAGCTCATGAACACGACTTGCAAATGGATTCTGGTGATCCTATCATTGT
GTGGGTTGAATATGACGGCCCCAAGAAAATAGTGAACGTCACTATAGCTCCATTGAAG
CACAAAAGAAAACCAACAAAGTCACTCCTTTCTTATCCAATTGACCTGAAACCTTTCC

TAAAGGAACAAATGTTCGTCGGCTTCTCTGCTTCGACCGGAGATAAAACAAGCTCTCA
TTACATTTTGGGATGGAGTTTTGCAATGAATGAACCAGCACCTCCCCTGGATTACTCTC
TTCTTCCCAACCCACCAAAAGAGCAGGATCCTCCTTCTTCATCACCCAACTCCCGCTAT
AAGGTTTTTGTAGCAGTCGTTTCTGTTATAGCCATTTTGGGGATTTTTTTCTTAGCTTTCT
GGTATAGAAAAACCTGGCACACTGAGAGACTTGAGGATTGGGAAAGAGACTGTCCTC
ACAGATTCCACTACACAGATCTGTACACAGCAACAAAAGGATTTAAAAGTAGCGAACT
AATTGGAATTGGGGGTTTTGGCTCAGTGTATAAAGGTCAGATACGTTCAACAGGAATTG
AAATTGCAGTGAAGAGAGTAAGAAGAAATTCAGGCCAAGGAATGAAAGAATTCGCAG
CAGAAATCGAAAGCTTAGGACGATTAAGACATAAGAACTTGGTAAATCTCCAAGGATG
GTGCAAGAAAAAGAACGATCTACTCATAGTTTACGATTATATACCAAATGGAAGCCTCT
ATTCACTTCTATATCATCCAAAAAACAACATTATATTGAATTGGAAACAAAGATTCAACA
TCCTCAAAGGAATTGCAGCAGGATTACTATACCTTCACGAAGAGTGGGAGCAAGTAGT
AATCCACCGAGACGTGAAGCCCAGCAATGTTCTAATAGACGCGGACATGAATCCTCGA
TTGAGCGATTTCGGTCTAGCCAGGCAATACGACCACGACGAAGCATCGCACACGACGG
GCGTCGTCGGTACGATCGGGTACATAGCACCGGAGTTGGTACGGACAGGGAAGGCGTC
GAAAAGCACGGATGTGTTTGGATACGGAGTTCTGCTACTGGAAGTGGCGTGTGGAAG
AAAACCGCTTAAGTCGGATAACTTCATACTAGTGGATTGGGTGATGGAGCAATATGAAA
AGGGAAAAATTCTTGAAGCGGCTGATCCGAAGTTGAATTGGGAATATGAGGCAGAGG
AGATGAAGATGGTTCTGGTATTAGGACTTCATTGTAGTCACCAGATAGCTGAAGCTCGG
CCGACGATGAGACGGGTGATGAGAATCCTTGACGGAGATGATAAAATTGCGGCGGTGG
AGGGGTGGGATTGTTCTCAAAGTTACTCAAAATCTAACTCAAGAATGACTGAAGTGAT
TTCAGCAACGTCTTACCGTTCCTCCTCCATCGGCGATATTTCAGAAACATCCATAGATGC
TGGCCGATAG
> gene:Csa4G296250
ATGGCATCTTCTTCTTTTTTGTTCATCATTTTTTTCTTGTCTCTAATTTTTCTCTCCACACA
TACCTTATCTTCTCACTACACCCAACTCTTCTACCCTGGTTTCTATGGATCCATCGACATA
ACTTTGAAAGAATCTGCAGAAATTGAAAGCAATGGAGTTTTGAGGCTCACCAGTCGTA
ATTCCCGAAACAACATCGGCCAGGCCTTTTACTCCTCTCCAATCCAGTTCAAAAACTCC
TCCAGCGACGGTGGCCGTGGACCCTCTTTCTCCACTTGTTTTGTCTTCTGTATCATCCCC
GAGAACGAGGGCGGCCATGGATTTACTTTCGCCATTGTCCCTTCTAAAGATCTCAAAG
GCATTTCTCAGAGGTTTCTTGGACTCTTCAACGAATCCAATTTCTATGGGATACTTTCAA
ATCATATCTTCGCTGTGGAATTCGATACAATCTTCGATGTTGGGATCAAAGATATAGATA
ACGATCATGTGGGGATTGATTTAAACAGCTTGATTTCGAATGCCACTGTTCACGCTGCT
TATTTTGACGAATTGGGTAAAGTTCATAATCTCTCACTTCAAAGTGGGAAGCCGATTAA
AGTTTGGATTGATTATGATTCTGATGAAATCACTCTCAATGTTACGATTTCCCCTTTTAAT
TCAAAACCCAGAAACCCAATTCTGTCTTACAGAGTGGATCTTTCATCGATTTTTTATGA
AGAAATGTACATTGGATTTACTGCATCAACAGGCTTACTTTCTAGAAGCTCACAGTTCA
TTTTAGGTTGGAGTTTCGCCATTAATGGCCAAGCTCGAGATCTTGATATCTCTTCACTTC
CTTTACCCAAAAAGAAGAAAACAAGGGAGAAAATCAGTCTCCCTGTTTATGTATCTATA
ACAACTGCCAGTGTTTTTGTGATCTCTGTTTTCGTTTTGGGTTTTTATCTCTTACGAAAG
TGCAAAAAAATCCGAAGAAATCGAACCGTGGGAGCTTCAATTAGGACCTCATCGATATT
CCTACAGAGAATTAAAAAAAGCTACAAGAAATTTCAGCGAGAAGGAGCTTCTGGGGTA
TGGCGGATCTGGGAAAGTATACAGAGCAATCCTTCCAATTTCGAAAACCCAAATCGCC
GTTAAGCGAATATGTCACGATTCAAAACAGGGTCTCCGGGAATTCATGACGGAGATCG

CCACAATTGGAATGCTCCGTCACCGAAATCTGGTTCAGTTGTTAGGATGGTGTCGGCG
GGAACGAGATCTGCTTCTTGTTTACGAGTTTATGGAGAATGGAAGCTTAGATAATTACT
TATTCGACGATCCAGTAAGGATTTTGGATTGGGAAGAGAGATTCAAAGTGATTAAGGG
AGTTGCCTCTGCTCTGCTTTATCTTCACGAGGGGTATAAACAGGTGGTGATTCATAGAG
ACGTGAAGGCAAGCAATGTGTTATTAGACGGCGAATTGAATGGAAAATTGGGGGATTT
CGGGCTGGCAAAGGTGTACGAACACGGATCGGCACCGGATACCACTCGGGTAGTCGG
AACTTTGGGGTATTTAGCACCAGAGCTGCCAAGAACAGGAAAATCGACGACGAGCTC
CGATGTATACGCGTTTGGGGCTTTAATGCTGGAAGTGGCATGTGGGCGGCGGCCAGTG
GAGGTGAAGGCGTTGCCGGAGGAGATGACGTTGGTGGATTGGATTTGGGATAAATACA
GAGAAGGGCAGGTTTTGTCGGTCGTGGATTCGAAGCTCCAGGGAGTTTACGACGAGG
TAGAGCTGACGATGGTGTTGAAATTGGGGGTAATGTGTTCGAATAATGTGCCGGAGCA
AAGGCCGAGTATGAGGCAGGTAGTGCGGTGTTTGGACGGAGAAATTGGCGTCGTCGAT
GAATGGAAGTCGCCCGGCGGCGGAAGTAAGGGCGGTGGGGTGGGGGATTTTCTTGGG
TCATTTACGTCCACGTCCATCAGTGGAGAAAGCTCCGGTTAA
> gene:Csa4G296230
ATGGCGGCCATCGTCTTCCTCACCTTAATACTTTTGATTTCCATTTCTCCAGCTTCTGCA
GATCAAAGATTCATCTTTAATGGCTTCAATTTCGGCGATCTCTTCCTCGACGGCGTCGCC
GAAGTAACCTCCGATCGTCTTTTGAGACTCACCAACGACTACGACCAACAAATCGGCC
ATGCCTTTTACCCCAATCCCATCTCCTTCAAAACCCCATCAAACAATAATTCCTCGCTTT
CTTCCTTCTCTGCATCTTTCGTCTTTGCTATAATCTCAGAATATGACGATCTCGGCGGCC
ATGGAATCGCCTTCGTCGTTTCTCCGACTAGAGGACTCCCCGGAGCTCGCCCAAGCGA
AAACCTCGGTCTATTCAACGAGTCCAACAACGGGAAAGAGACGAATCATATCTTCGCC
GTTGAACTCGACACGATTCAGAATCTCGATCTTAGAGACATCAACAGGAACCACGTCG
GAATCAATATAAACGGATTAATGTCAGAACAATCGGAGCGAGCAGGGTATTGGGTCGA
CGGAGAATTTAGAAATTTGACGCTAATCAGTGGCGAACGGATGCAAGTTTGGATTGAAT
ACGATGGACTCAAAAAGCAAATTAACGTTACCCTGGCTCCAATTGAAATCAGAGATAA
ACCCAAAATCCCTCTCTTATCTTACCGGCGAGACTTATCGTCCGTCATCAACGACATCAT
GTACATCGGATTCTCGTCCTCAACGGGCTCCATTACGACGTTGCATTACGTTTTAGCTTG
GAGTTTCAACGTCAATGGCGAAGCACAGAAGATTAATCTGTCTCAACTCCCCAAGCTT
CCACCACGTACAAAAAAAAAAACCGAGCCGATCCAAATTGTTAACAATAGGTTTGCCGT
TGGTTTGCGTTGCATTAGCTTTAATGACAGTTTTGGGGTTGATTTATTTCATATACCGGA
GACGGAAATTCGCAGAGATTCTAGAGGATTGGGAGCTGGAATACGGACCGCAAAGATT
CAAATACAAGGATTTATACAAAGCAACAAAGGGATTCAGAGAAAAAGAATTTCTGGGA
TTTGGTGGATTCGGTAGAGTATACAAAGGGGTATTACCAAATTCGAAAATCGAAGTAGC
GGTGAAGAGAATCTCTCACGAATCAAGACAGGGGATGAAGGAATTCGTGGCGGAGAT
AGTGAGTGTAGGGAGGCTCCGGCACCGGAACCTGGTGGCGCTGTTGGGGTATTGCCG
GAGGAAAGGAGAGCTGTTTTTGGTGTATGATTATATGAGAAATGGAAGCTTGGATGCGT
ATTTACACGAGTGGCCAGAGATAACGATAAATTGGGAACAGAGGTTTGAGATAATAAA
AGGAGTTGCGTCGGGATTGTTCTATCTTCATGAACAGTGTGAGAAAGTGGTGATTCATA
GAGATGTGAAAGCGAGTAATGTGCTGCTTGACGATGAGTTTAATGGAAGATTGGGGGA
TTTTGGTTTGGCTAAAATGTACGATCGTGGTGCTGATCCTCGAACCACTCATGTCGTTG
GAACTCTTGGGTATCTAGCGCCAGAGCATATAAGAACAGGGAGAGCCACAACAAGAA
CAGACGTTTACGCATTTGGGGCTTTTCTTCTTGAAGTGGCCTGTGGAAGAAGGCCTATT
CATCCTCAAGAAGACTCCGACGATTTCATTTTAATGGATTGGGTTTTCTCTTGTTGGTCC

AACGGCAACATTCTCCGGACCGCCGACCCCAAATTGGGTGGCAACTTCGAACCTTCAC
AGCTCGAATTGGTTCTCAAACTCGGGTTGCTCTGTTCTCACTCCTCCCCTGCCGTTCGG
CCCACCATGTATCAGGTTCTGCAGTATTTGCAGGCCGAAGCGCCGCTGCCGGACCTCG
CCTCCCTCCGCTGGCGCCTTTCCGGAAATGGGTTTTTCAATTTGACGCCTCGCGACGGC
GATGAGTTGGATGATTTTACCGGCTCTGTTAGACAGTCATCCCTTCTTCTAGATTCAATT
TTCCGGTAA
> gene:Csa3G736960
ATGATGTTGAATTTCTCTTCAATATTCTCATCAATTACCTTCACATTACTTCTATTTTCCAT
TTCCAATACTGTCATTGTCGATTTCACTGCCACCGTCGCCGCCGCGGCCGAATTTGACT
TCGGCACTGTTGAACTCAGTAGTTTAAAGCTACTCGGCGATGCCCACTTGAACAACGG
GAGCGTGAGGCTCACTCGGGACCTCGCGGTCCCAAATTCCGGCTCCGGCAGAGTCCTT
TACGCTAAACCCATCAGATTCCGGCAGCCGGGGATTGATTACCTCGCTAGTTTCTCCAC
ATTTTTCTCTTTCTCTATTACTAATCTCAACCCGTCGTCGATCGGCGGTGGTTTGGCGTT
TGTTATATCCCCGGACGCTGAAACGCTCGGCGGCGCTGGTGGGTTGTTGGGGTTGGCT
GACGAGAGGGGATTGGGATTCGTGGCGGTGGAATTTGATACGTTAATGGACGTGGAAT
TTAAGGACATTAATGGAAACCATGTGGGATTGGATCTGAACGAAATGGTTTCGTTAGAG
GTTGAAGATCTTCAGGGAATTGGAGTCGATCTTAAAAGCGGCGATACGGTGAATGCGT
GGATTAAATACGATGGGTCGGCACGGATCTTCGAAGTATTTGTTTCTTATTCGAATTTGA
AACCGACAGAACCTCTAATGTCGTTCAATCTCGACCTTGATCCGTATTTGAACGATTTTA
TGTACGTTGGATTTTCCGGTTCGACACAGGGGAGTACGGAAGTTCACAGTGTGGACTG
GTGGAGCTTTGCGTCGTCATTTGACTCGGATTCAACACCTGGGTCGGTTCCGCCGCCG
CCAACGACGACGCTGATGAACCCAACGGCGAATGTTGTTCGTTCACCGCCACCGTCGC
AACCGCCATCCGGTTCAGATTCCGTCACGCAAAAGAACTTCAAGTCCACGTCCTGCCA
CAATGGGCTCTGCAAGCAAGGCGCTGGAGCGGTGGTCGGAGTTGTGACGGCGGGGGC
ATTTGTTTTAGCATTATTCGCCGGCGCGTTAATTTGGGTATATTCCAAAAAGATCAAGCG
GGTTAAAAAATCGGATTCCCTCGCTTCAGAAATCATCAAAATGCCTAAGGAATTCACTT
ACAAGGAGCTCAAAATCGCCACCAAATGCTTCAATTCGAACAGAATCATCGGCCATGG
AGCTTTTGGGACTGTCTATAAAGGGATTCTGCCGGAGACCGGCGACATTGTGGCGGTG
AAACGGTGTAGCCATAGTACACAAGGGAAAAATGAATTCCTCTCTGAACTTTCAATCAT
CGGAACTCTCCGCCATCGGAATTTAGTCCGTCTTCAAGGATGGTGCCACGAGAAAGGC
GAAATTTTACTCGTTTACGATTTAATGCCAAATGGGAGTTTAGATAAAGCTCTATTCGAG
GCTAGAACGCCGCTGCCGTGGCCTCACCGGAGAAAAATCCTTCTGGGCGTCGCTTCCG
CTTTAGCCTATCTGCATCAAGAATGTGAAAATCAAGTCATTCACAGGGACGTTAAAACC
AGCAACATAATGTTGGATGAAGGATTCAATGCCCGATTAGGCGATTTCGGATTAGCCCG
ACAAGTAGAGCACGATAAATCCCCAGACGCCACCGTCGCGGCTGGCACAATGGGTTAC
TTAGCACCGGAATATCTCCTCACCGGACGAGCTACGGAAAAAACCGACGTCTTTAGCT
TCGGCGCCGTCGTTCTCGAAGTCGCTAGTGGCAGACGGCCAATTGAGAAAGATAGCAC
CGCAGTTGGCGGTGGCGGAAAATTCGGTGCCAACAGTAATTTGGTAGACTGGGTTTGG
AGTTTACACAGGGAAGGACGGTTGTTGACGGCCGCTGACGGAAGACTGGGCGGCGAG
TTTGAGGAATCAGAAATGAGGAAAGTTTTGTTAGTTGGGTTAGCTTGTTCTCACCCGGA
TCCAATGACCCGACCCACAATGAGAGGCGTAGTGCAAATGTTAATTGGCGATTCAGAG
ATTCCGATCGTGCCTCGGTCTAAACCGTCCACAAGCTTCAGTACCGCTCATTTGCTTCT
CACTCTACAAGACAGCGTGTCCGATCTCAACGGCATGATAGCCATTTCCACTTCCTCCT
CCGAGCATAGCTTCAATGGAGAGGATTTAATTTCACTGGATGATCGGACGGTGAGCAAT

CCATCCATTGTTTGA
> gene:Csa7G067400
ATGGCTGCCATAATCTCCATTTCCAGTTATCTTCTTCCTCTGTTCTTCTTCTTCATCTACA
TCCTATCATTCTTCTCCACACTTACACTTGCAAATTCTTTAGCTTTCAATTTCTCATCTTT
CGATTCAAGTAATACCCACATATTCTACGAGAAAGCATTCCCATCAAATAGAACAATCA
AGCTCACTGGAGAAACTGTTAATAAAAACCAAAATTTCACAGGAAGAGCAACCTACTT
TAAGCCTTTTCATCTTTGGGACAAACCCTCTGGGAATCTATCAAGTTTTCAAACTCATTT
CTCCTTTGCTATTGATTCAGAAGGAGCAGAAAGGTATGGAGATGGGCTTACCTTCTTTT
TTGCTCCTAATAATTCTAGGCTTGATGCTGAGATCTCAAAGGGTAGTGGATTAGGGATT
GGGTATAATCCCAGTTTGACAAATTTAACATATAGTTCATTTTTTGCAATTGAGTTTGATA
TC
TTTAGTAATTTCTTTGATCCGCCTCAAAAAGTTGAACATGTAGGTATTGATATCAATTCA
ATGTCTTCTGTTGCTTACTCAATATGGAAATGTGATATTAAGAGCGGGAGGAGGACGGA
TGTTTGGATTAATTATGATTCTGCTACTTTGAATTTGAGTATTACTTTTACTGGGTATGAG
AATAATAAAACTATATTGCAGAGACTTAATCATGATGTAGACTTTAGGTTGACTCTACCT
GAATGGGTTACTTTTGGGTTTTCTGCTGCGACTGGAACTCTATATGCAACTCACAACAT
ATATTCTTGGGATTTTAAGTCAACTCTAAATTTGAATTCTGATTCTAATTTGGCCCCAAG
TCCTGGTCAGGGTAGCAAGGTAGGCCTAGTAGTTGGAGTGGGTGTTGGTGTTGGTGTA
GTGTTCTTAGTCTGTGGTTTGATTATAGTTTGGATCTGTTTTTTGAAGAAAAGAAAGAA
GAGGATGAAAATGAATTGGGAAGAGGATGTTGTGTTGGATGATAGTGAGTTTGAAAAG
GGTAAAGGACCTCGGAAGTTTTCGTATAGCGAATTGGCTCGAGCAACGAATAACTTTT
GGGAGGATGAAAAGCTTGGGGAAGGAGGATTTGGTGGCGTTTATAAAGGTTTCTTGAG
GGACTTAAATAGCTATGTTGCTGTGAAGAAAGTGTCAAAAGGGTCTAGACAGGGAATA
AAAGAGTACGCATCTGAGGTGAAAATCATCAGCCAGTTGAGGCATAGAAACTTAGTGC
AACTCATCGGTTGGTGCCATGAAAGAGGTGAGCTCTTGCTGGTTTATGAATTCATGTCA
AATGGAAGTTTAGATGCCCATCTTTTTAAAGAAAACAACTTCTTAACATGGGAACATAG
ATACAAAGTTGCTCAAGGCATTGCCTCGGCTTTGTTATATCTGCATGAAGAGTGGGAAA
AATGCGTGATACATCGAGATATAAAGTCAAGTAATGTGATGTTGGATTCAGATTTCAATG
CAAAGCTTGGAGATTTTGGGTTGGCTAGGCTAGTGGATCATGCAATAGGCTCACAAAC
AACAGTTCTTGCTGGCACAATGGGCTACATGGCTCCTGAATGTGCTATATCAGGAAGAG
CCAGTAAAGAATCAGATGTCTTCAGCTTCGGGATTGTAGCATTGGAAATTGCTTGTGGA
AGAAGACCCTACAACCCTAATGTAGAGGAGGCAAAAATGGTGATGGTAGAGTGGGTTT
GGGAGCTTTATGGGAATGGAAGGCTTCTTGAAGCAGCCGACACGAAACTCCATGGAA
GCTTTGAGAATGAACCTCAACAACGTCAACAGATTGAGTGTTTGATGGTTGTTGGACT
GTGGTGTGCTCATCCAGATATAAACTGTAGACCATCAATAAGACAAGCAATTCATGTGA
TGAACTTTGAAGCCTCATTACCTGTTCTTCCTTTACAGTTCCCTACACTTGCTTATCATC
ATCATCCTCTTTCAGTGAACAGACCAATTATTTCTTCTTCATTTTCATCAACTCAAGATA
GTGTTGTTTCTCAAAGTACTGGAAATGGCTTCAACTCTACTAATGTCTTAACTACTTCAC
AGGAGACTACAACTTCTTCAACTTCTTTTTCTGCATCAACTTCACTTTTGAATACACGG
TAA
> gene:Csa7G029930
ATGAATTTGATGAGAAAGATGTTTATGTGTAAGAGAGAATTGAGAACCTTTGTGGTTGT
TGTGACTTCTTTGGTGTTGCTCTTTGGACCTGTGAGTAGTCAACCGATCCTTGAGCATC
GTTTTTCCTATGTTGGATTCAATGAAAGGGAGAACAATCAAGCTTTCACCTTCACACCA

AGTTCTTCCATCGACGGCGGGGCGCTGCAGCTAACACCCGACTCGCAGAACGATGTTG
TTAAGCTTCAAAATACATCTGGCCGCATTATGTACCATGAGCCGTTCAAGTTATGGTTAA
ATGATAGTGATAAAAAGGAGAAAAGTGACACTGTTGCTTCATTCAGCACATACTTTTAC
ATCAAT
ATTTTCCGAAGGGAAGAATGGACTGCTGGTGAGGGACTTACTTTTCTTATAGCTCCGAC
TTCTGTTGTACCTGAACAGAGCTGGGGGCAGTGGATGGGCCTCACCAATGAAACCATC
GACGGCGACGAAAAAAACCAAATTGTAGCTATCGAATTCGATACCCAGAAGCAGGATT
TTGACCCTGACAACAACCATATTGGTCTGAACATCAACTCCGTTAAATCAAGGAAGAC
TGTTCCTCTGAAAGAAGCTGGCATAGTTCTATCCCCAGAGGTTGGAACTAATCACAGTA
TCTGGGTTGAATATGATGGCAAAGCCAAACTTTTGCAGGTTTACATGTCCATAAACAAA
GATCCAAAGCCTAATAAACCTCTTCTCAATGAAACCCTAAACCTGAAAGAATTTGTGA
AGCAAGAATCATTCATTGGATTTTCTGCTTCTACAGGAAGCCCAGAAATTCAGTTGAAC
TGTGTCTTGGAGTGGACACTAGAAATGGAGCGTCTGCCAGAGAAGAAAAATCTAACAT
GGTTGAAGATCCTTGCTGGGGTCGGAATTCCGGTACTGACGATCGCAATTCTGGTAGG
GGTTTGGTTGTTTGTTGGTTATAGGAAGAAGAGGAGAGAGCACGTGGATGAGGAGTC
AAATGTTCAGGGCACGTTGAAGAGATTGCCTGGAATGCCCAGAGAGTTCAAGTACAA
GGAACTAAAAAGAGCGACTCATAACTTCCATGAAAGCATGGTTCTGGGAAATGGAGGA
TTTGGGATTGTGTACAAAGGGGTTCTGCAGGACAAGGATAGGGACATTACCGCATCTT
CAAATTCTGGTTCCAGGCTTGAAATTGCTGTCAAGCAATTCTCCAGGGATAGCATTAAG
AGCAAAAGTGATTTTCTTGATGAGCTCACCATTATTCACCGTCTTCGCCACAGGAACCT
TGTCCGTTTAGAAGGGTGGTGCTACGAGAAAGGGAAGCTTTTATTAGTATATGACTTCA
TGCCGAATGGAAGCCTCGAGAATCACCTATATGATGTTGATGAGCAGAATGTTCTCAAC
TGGGGACATCGCTATAAAATTCTTTGCGGAGTTGCATCTGCCTTGCATTACCTACATAAT
GAGTATGACCAGAAAGTACTGCACCGGGATATCAAATCAAGTAATATCTTGCTTGATTC
TGAATTCAATGCTCGTCTAGGGGATTTCGGCCTTGCTAGAGCCTTGGATCCTGAAAGAA
ACTCTTATGCAGACTTGCATTGTGGTGGAGTTGCAGGCACAATGGGTTATGTTGCTCCT
GAATGCTTCCATGAAGGGAGGGCTACACCTGAATCTGATGTATATGGGTATGGGGCAGT
GGTGCTTGAGATCGTTTGTGGAAGGAGACCAGGAGCTGTTGTTGAAGACGAACAAGA
TCACTATAGCCTAATTGATTGGGTGTGGAAGCTACACCGTGAAGGACACATTGAAAAA
GCTGTGGATAACCAGCTGGGTAATGATATTGTGGTTGATGAAGCTCGAAGGCTTCTACT
ACTTGGGTTGGCATGTTCACATCCCGTGGCAAGTGAAAGGCCACAAACTCAGGCTATA
CTCCAAATTTTAAATGGAGCTGTGCCTCCACCTCATGTACCTCCATTCAAGCCAGTTTT
CATGTGGCCTCCCATGAGCAGTTCTTCCACAAGTTCCATTCTCACTTCACTGTCAAACA
CGAATAACTCTCTCTCATGA
> gene:Csa3G734050
ATGGCTTCTAATTTTGATCGCCCCTTACGAAGAAATGGTGCTTATGATGCTCGGTTACAC
GGAAGAGAGATGAACAAAAATCCCATGTTTTTCAATCTCTCAATTGTGGTGTCTCTGCT
TCTAGTCTCAGTAGTCTCATCAGCTGCTTCTAAAGGTACTGATTTCATCTTCCATGGCTT
CCAATCTGCAAATCTATCACTCGATGCTATGGCTGTTGTTACCTCAAATGGCCTCTTGAA
ACTGACCAACGAGACCAGGCAAAAAATTGGCCATGGGTTTTATCCTAATCCTGTAAATT
TTGTCGATTTCTCTCATGGCGTTTCATCTTTCTCAACTACTTTTGTCTTTGCCATTATTTC
TGAATACCCAAATCTAAGTGGCCATGGAATTGCCTTTGTGGTGGCTCCGACGAAAACTT
TCCCCGGAGCTGAGCCAAGTCAGCATCTTGGCATTTTTAATGACAACAACAACGGTAA
CACAAACAACCATATTTTCGCTGTAGAACTCGATACAATTCAGAATCTAGAGTTGAAAG

ATATAGATGCGAATCATGTTGGTATAGATATAAATGGGTTGATTTCTAAGAAAGCTGAAA
CTGCTGGGTTTTATCCTTCCAACAATGGGGAATTCAGAAATTTGAGTCTTATAAGTGGC
CAACCAATGCAAGTTTGGATCGAATATGATGGTATCAACAAAGAGATCAATGTCACTTT
GGCACCAATTAACATACCCAAACCCAAAATTCCACTTTTATCTTACGTCTGGGACCTTT
CGTCCGTCATAAAAAACTCTTCCATGTTCGTCGGGTTTTCATCCTCCACTGGCTCTGTTT
CAACTTCTCATTATATTTTAGGTTGGAGTTTCAGATTAAACGGCCAAGCTCAAAGTCTT
GACATTTCTCGTCTTCCAAAGCTGCCTCAGAAAAAACATAGATCCAAAGTTTTAACAAT
TGGGTTGCCTTTAATTTCTGGAGGGATTGCTTTATTGGTTATTTTATCCATTGTCCATGTT
ATCAGAAGGAAGAGGAAGTTCGCCGAGCTGCTCGAAGATTGGGAGCTTGATTATGGGC
CTCATAGGTTCAAATACAAAGACTTATACACAGCTACAAATGGATTTAAAGAAAAAGA
AATTCTGGGTTCTGGAGGATTTGGACGAGTCTACAAAGGTGCATTACCAAAATCAAAA
CTTGAAATAGCTGTAAAGAGGGTTTCTCATGAATCAAGACAAGGAATGAAAGAATTTG
TAGCTGAGATAGTTAGTCTTGGGAGGCTTCGTCATAGAAACCTTGTACAACTTTTAGGC
TACTGTAGACGAAAAGGAGAGCTGCTTTTAGTATATGATTATATGCAAAATGGAAGCTT
GGACAAGTACCTGTTTAACGAAACAAATCCAAGTTTGAACTGGAGCCAGAGATTTCGA
ATCATAAAAGGAGTGGCTTCAGGGCTGCTTTACCTGCATGAAGAATGGGAGCAAACTG
TTATTCACAGAGATGTAAAAGCCAGTAATGTCTTACTAGACAATGAGTTAAATGGAAGA
TTAGGAGATTTCGGTTTGGCAAGACTGTATGATCACGGAACAGACCCTCAAACAACTC
ATATTGTAGGAACACTCGGATATTTGGCTCCAGAGCACACGAGATCCGGCAGGCCGAC
GACTCAAATGGATGTGTTTGCTTTTGGGGCATTTCTGCTGGAAGTAGCAACAGGAAAG
AGGCCAATAGAGATTCGAGGGATGATGGAAGATGTAATATTGCTGGATTGGGTATTGTT
ATGTTGGATGAGAGGAGCCATTGTTGAGGCCAAAGATCCAAAGTTGGGAACAGAGTAT
GTGACAGAGGAGATGGAAATGGTTCTGAAACTTGGATTGTTGTGTTCACAATCTAACC
CAATGGCGAGGCCAAGCATGAGGCAAATTGTGCAGTACTTGGAAGGAGATGCTGTCAT
GCCAGAGATGGGTTCTATAGAAACGTTAGGTGGTGGTGGTGGATATGAAGGTTTTGATG
ATCTTGGCATGTCGTATAATTCTTTTTGGATAAAGCTATTGCATATTCTACTTCTTCACT
TGAAAGAGGTTTTGCCCATTCTTCTGATGCGCAGTCTCTCCTGTCAGGTGGCCGTTGA
> gene:Csa2G439150
ATGCTAACCCACATCTTCCTCCTCTTCCTTGTTTTTCCCTATCTTCCTCTCTTAGTTGACT
CAATTTATTTCAAAATCGATCAAATCAAACCAAACGAAAACCGTTTACTTTACCAAGGA
GATGCAGTACCTAATAATGGAGGAATTATATTCAGTGATCCTGCATATTCCTGTCTTGTT
GGTCAAGCTATTTACAAAGATGCTATCCCAATTTGGGACTCTCAAACAGAAAAACTCA
CTGATTTCACAACCCAATTCTCCTTCACCATTGATACTCAAAATGCTTTACACTATGGAA
ATGGGGTTGCTTTTTTTCTTGGCTCCAGCTGGTTTTCATATCCCTCCAAACTCAGCTGGTG
GGTATCTTGGCCTTTTCAATAAGACCTATACTGAATCATCCATAAACCAAATTGTTCATG
TTGAGTTTGATTCTTACCCTAATGAATGGGATCCTAATTTTGAACATGTGGGTATCAACA
TTAACTCTGTTTCTTCCTCTAATTTCACGAAGTGGAATGTCAGCTTACATAGTTTGGACA
CTGTTGACGTGTTTATCTCATATGATTCAACCACCAAATACTTGAGTGTTTCATGGAATT
ATGAAAAGACACCAATTTCTCTTGAAAATACAACTTTGAGTTACATGGTTGATCTTATG
AAGATTCTTCCTCAATGGGCTACAGTTGGATTTTCAGCTGCAACGGGGGCATATTTAGA
GAGACATTTGCTATTTTCTTGGGAGTTCAATTCCAGTTTGGAAATGAAAGAAACAGTTG
GAGTTGGTACTGAAAAAAATGGGAAAAAAGTTGATGTAATTGTGGGTGTGACTGTTTC
AGTAGGAGCTTCAATTCTCATGGCAATTGTAGCTTTTGTGGTACGTCGGAGATTGAAGC
AAAAGAAAAGAAAATCAGAAAAAAAGGTGGCTGAGGAGATCAACTTGACATCCATTA

ATGATGATTTGGAAAGAGGGGCTGGACCTAGAAGGTTTTCTCATAAGCTTCTTGCCATG
GCTACCAACAACTTCTCCAATGAAAGGAAGCTTGGTCAAGGAGGATTCGGAGCAGTGT
ATAGAGGGTATATACCAGACATAGATTTGGCAGTGGCCGTGAAGAAAATCTCAAGGGG
TTCAAGACAGGGGAGGAAAGAGTATATAACTGAAGTGAAGATCATTAGTCGGCTTCGC
CATCGAAATTTGGTGCAACTCGTTGGTTGGTGTCATGATAAGGGTGAGTTCTTGTTGGT
TTACGAATTCATGCCTAATGGCAGTCTTGATTCTCATCTCTTTGGTAAGAGGGCCCATCT
TGCTTGGGCTGTGAGATATAAAGTTGCTTTAGGTTTAGCCTCTGCTTTGCTCTATCTTCA
CGAAGAAGGGGAACAGTGTGTGGTTCATAGAGATATTAAATCAAGTAATGTTATGTTAG
ATTCGAACTTTAACGTCAAGCTTGGAGATTTTGGGTTGGCTCGATTAATGGACCATGAG
TTGGGTGCTCAAACAACTGGGTTGGTTGGAACTTTAGGCTACTTAGCTCCTGAATACAT
CAGCACAGGTAGAGCTAGTAAAGAATCTGATGTGTTTAGTTTTGGGGTTGTTGCTTTGG
AGATTGCCACGGGAAGAATGTCAAGAACCTCCATGGAAGCTGAATCTCATAAGGGTCT
GGTGGAGTGGGTTTGGAATCTTTATGGGAGTGCGCAATTGATTGATGGCATGGATGAGA
AAATGCAATCTGATTTTGACAAAAAACAAGTGGAGTGTTTGATGCTTGTTGGATTATGG
AGTGCTTATCCTGACCCTAATCTTCGACCTTCCATAAAACAAGTAATTCAAGTTCTTAAC
TTTGAGACAACCATGCCAAATCTTCCAAATAAAATGCCTGTTCCTATCTATTCTGCTCCT
CCCACATCGATGAGTTCAAATGAAGCTTCCATTACAGTAAGTCTTGACATGGGTCGTTA
A
> gene:Csa3G730910
ATGTTTGATATAAAATCTGAACCAGTTTCAGATGTTTCTTCTTTCAGCACATCTTTTGTG
TTTGCGATAGTTCCATCAAGCTCTGGTCCACCAGTTGGGTATGGCCTCGCCTTTGTTATG
GCTCCATCAACCCAATTTCCAGATGCTGCAAGTGAACATTACTTGGGATTATTCAACCC
CTCCAACAATGGTGACCCTTCGAATCATATTTTTGCAGTCGAATTTGATACTGTTAATGG
CCACGATGACGAAACGAATTTTAAAGGCAACCATGTTGGAATCAACAAGAATGGTGTG
CGATCAAGTGCGTCTGAATCGGCTGAGTATTCTGACTATGGCTCTGATGTGAAGACGGA
GGTGTATTTGGATACTGGTGATCTAATCCAAGCTTGGATTGATTATGATGGCCGTAGTAA
GGTAGTGAATGTCACAATAGCTCCAGCAAGTGTAATCAGACCAACTGAGCCATTGATTT
CATATCCCATTAACTTGACTTCAGTTTTGAATGAGAGGATGTTTGTGGGATTCTCTGCAT
CAACT
GGAAAAGAAACAAGCTTCCACTACATTTCAGGTTGGAGTTTTGCAATCAACGAATCAG
CACCTCAGTTAGATGTTTCTCAACTTCCTCCACCACTGAAAGTGCAAAGTCCTCCGCCT
TCATCGCCGTCAACATTCAATCCTCTAGTCACTGTTGTCGTTCCAATATTATCTGCCATG
ACACTCATGTTAATTTTGTTCTTAGCCTCCATTTTCAGAAGAAGATTGCGAGGTGAGAA
CCTTGAAGAATGGGAAAGAGATTGTCCTCACAGATTTAGTTACAGAGATCTTTACAAA
GCAACAAAGGGATTTAAAGACTCCGAGCTCATCGGATCAGGAGGCTTTGGCTCAGTAT
ACAGAGGTGTCTTACCTTCCACAGGATGTGAAATTGCTGTAAAGAAGATAACAAGAAA
TGCGAGTCAAGGAATGAGGGAATTCGCTGCAGAAATTGAAAGTTTGGGCCGACTAAG
ACACAAGAACTTGGTAAATCTCCAAGGATGGTGCAAGAAACAGAACGATCTTCTCTTG
GTTTATGACTATGTTCCAAACGGAAGCTTGGATTCTCTCCTTTACCATCCAAAAGACAA
CTTGGTACTGAATTGGGAACAAAGAATCAACATCCTCAAAGGAGTTGCTGGTGGATTG
TTATACTTACACGAAGAATGGGAACAAGTCGTGATCCATCGAGATGTTAAGCCAAGTAA
TATATTGATAGATATTAGCATGAATGCTCGACTAAGCGACTTCGGCCTTGCAAGGTTATA
CGACCATGACCAAATATCACATACAACCAGCGTGGTTGGCACAATCGGGTACATCTCAC
CCGAGTTGGCTCGCACCGGAAAGGCATCGAAAACTACAGATGTCTTTGCCTATGGGGT

ACTAATTCTTGAAATGGCTTGTGGAAGAAGACCTCTAGAATCAGATATATTCATATTGGT
AGATTGGGTAATGGAATGCTATGAAAAAGGTCGTGTTCTTGATGCAGCTGATCCAAAGT
TGAATGGAATTTATGACATGGTTGAGATGGAAATGGTTTTGAAACTTGGACTTCTTTGT
TCCCATTACAACCCTGAATCTAGGCCAAGCATGAGGCAAGTCACAAGGTTTCTTAATGG
AGAAGACCAAATTCTTGCTTTTGATCCTTCACCTTATTCTCAAATCACATTTCAATCAAG
CTCCGGATTCACTCAATTTATTCCACCTTCTTCCCGTACTTCAACCGCCAGCTTTCTCTC
TTCAACTTCCATCGATGTAGGCAGGTAA
> gene:Csa1G073890
ATGGCCCCATACAGGGACGTCCTTCCTGGGCACGGCTTAGTTTTCATTTGCGTACCGAC
TACTGGAATCGAAGGAACTTCTGCTGCTCAGAATTTGGGCTTTCTTAACTTCACCCTTA
ATGGAAACCCCAATAACCATGTCTTTGGAGTTGAGTTCGATGTTTTTGAGAATGAGGAA
TTCAGCGATCCCGATGATAATCATGTCGGAATCAATTTGAATTCCTTGACGTCTTTTATC
ACCGAAGAAGCTGGGTTTTGGTCTGACGGTGGTCCGAATGCGGCGGGTACGCTCAAC
AGACTTAGGCTTAATAGCGGCGAAAATTATCAGGTTTGGATTGACTATTCCGATTTCAG
GATGAATGTTACGATGGCTCCGGCAGGGATGAAGAAGCCGCGGCGACCTTTGATTAAT
ACGTCTCTGAATCTCTCTGGAATTTTCATGGATGAAATGTATATGGGGTTCACGAGTTCA
ACAGGACAGTTGGTTCAAGGTCATAACATTTTAGCTTGGAGTTTCAGTAACACTAATTT
TTCGTTGAGTGAGAGTTTGATCACCACTGGATTACCCTCCTTCCTCCTCCCAAAAGACC
CAATTACCAAATCGAAGTGGTTCATCGCCGGAGTGACAGCCGGCGGATTCCTCGTCGT
TTGTTTCTTCGCAACGATTTTAGGGATTTTGATAGCCGACTACCGGAGAAAAGCGAGGT
TAAGAGCCGAAATGGAAGATTGGGAATTAGAGTATTGGCCACACCGACTGACATATCA
AGAAATCGAATCGGCAACGAAAGGATTTGCAGAAGAAAACGTGATAGGAATCGGAGG
GAATGGGAAAGTACATAAAGGGATTTTGGCCGGAGGAAGTTCAGAAGTTGCCGTGAA
ACGAATCTCTCACGATAACGACGGGATGCGTGAATTCTTAGCTGAAATCTCAAGTATCG
GAAGATTAAAACACAGAAATCTTGTAAGTTTGAAGGGTTGGTGTAAAAAAGAGAAGG
GAAATTTCTTGCTGGTTTACGATTTCATGGAAAATGGAAGCTTAGACAAATGGGTATTT
GATTGTGATGAGAGAAACATGTTGAGTTGGGAGGATCGAATCAGAGTATTGAAGAACG
TAGCGACAGGGGTTTTGTATTTACACGAAGGATGGGAATCAAAGGTTCTTCACAGAGA
CATAAAAGCCAGCAATGTGTTGTTAGACAAAGACATGAATGGAAGGCTAGGGGATTTC
GGGTTAGCTCGAGTCCACGGTCACGGTCAAGTTGCAAGCACGACTCGAATCGTTGGCA
CAATGGGTTACATAGCACCCGAGTTGATTCGAACGGGACGAGCCTCAACTCAATCCGA
CGTGTTTGGTTTTGGGGTATTGATTTTGGAAGTGATGTGTGGGAGGAGGCCAATAGAG
GAAGGGAAGCCGCCATTGGTGGATTGGGTTCGCCAGTTGGCCATTGAAGGGAAAATTA
CGGCAGCGCTTGACAGCCGGCTGAGGAAGAAGGGTGAGTGGAACGAGGAGGAAGTG
GGGAGAGTATTACATTTGGGACTGGTGTGTGCGCATGTTGACCCAAACAATCGGCCGA
CGATGAGGCAAATTGTGAAAGTTTTGGAAGGGAAGATTGAGTTGGATGAGAGTGAGA
GTGAGAGTATGAATGCTTATCTGCTTCAAAGATTGAAGTCAGAAGGAATGTTGTGTGAT
TCTGGTGTTGGTTTTGGGAAAAATCTGCATCCAACATTTGAGGACTTTCTACAATCTTAT
TCAACTTCACTCTCTTGGACCAATTCTTCTGTCACTGGCAGGTGA
> gene:Csa3G115060
ATGCCCATCTTTCTTGGAACTCATAGAAACCACCATTTCCAATCCCAACCTCTTCAATTC
TTCCTCTTCTCTTTAATTCTCTTCAAAGTTCAATCACTTTCCTTCACCTTCCCCAACTTC
CAACAAAACAATCCCAACTTATTCTTTGAAGGCGATAGCTTCACCAGCAATGGTCTTAT
TCAACTCACAAAGAACCAAGCTGATGGCCCCCTCACCGATAGCTCCGGTCGAGCTTCT

TACGCTCAACCCGTTCGCCTATGGGATGCCGCCACAGGACAGGTTACAAATTTCACCA
CCCATTTCTCCTTTAGAGTCACCCAACTTAATCAATCTTCATTTGGTGATGGAATCGCCT
TCTTCATAGTCCCTTACGAATCGAAACTCCCCGCCAACTCAACTGGGGGATTTCTTGGA
TTATTTAGCTCGGATTTGGCTTTTGATTCCTCAAAGAATCAAGTTTTTGGAGTTGAATTT
GACAGTAAACAAGATGATTGGGATACAAGTGATGACCATCTAGGAATCAATGTGAATTC
CATTAAATCCATTAATCATCTTGATTGGAAAAGTAGCATGAAAGATAGCAGAACAGCCA
ATGCATGGATTACTTACAATTCTGCTACTAACAATTTGTCTGTGTTTCTAACTTACGATAG
TGATCCAATTTTTACTGGAACTTTCACCATCTCAACTTTCGTTGACTTGAAAAGCTTCTT
GCCTGAAAGGGTCAGAGTTGGATTCTCCGCAGCCACAGGGAAATGGTTTCAAATACAC
AACATAATTTCTTGGTCTTTCAACTCAACTTTGGATGATAATCTTGGTGGTGGAGACAA
GAACAAAAACACTGGTTTAGCGATTGGGCTTGGTGTTGGGCTTGGTGTTGGGATATGT
GGATTGATTTTGTTGGGATTGTTTTGGTGGGGTAAAAAGTTGCGGAGAATGGAAGACG
ATGTGGATGATTCTATAGATGATGAATTTGAGAAAGGAACGGGCCCAAAAAGGTTTACT
TATAGGGAATTAACTCGAGCAACAAAGAATTTTGATGAGGCTGGTAAGCTTGGAGAAG
GAGGATTTGGAGGTGTTTACAAGGGTTTGCTAACAGAATCCAATACAGAAATAGCTGT
GAAAAGGGTTTCAAGAGGATCAAGACAAGGGAAAAAAGAGTATATTTCTGAAGTGAA
GATTATTAGTCGTTTGAGGCATAGGAATCTTGTTCAACTCTTTGGTTGGTGTCATGAAA
AAGGTGAGTTTCTTTTGGTTTATGAGTTTATGCCTAATGGTAGCTTGGATACTCATCTATT
TAGAGGTCAAACGATGCTAAGTTGGCCTGTTAGATACAAAATAGCAATAGGATTGGCTT
CTTCTTTGCTATATCTTCATGAAGAATGGGAACAATGCGTGGTGCATAGAGACATTAAG
TCCAGCAATGTAATGCTGGACTCAAATTTCAACGCTAAACTCGGCGATTTCGGCCTTGC
AAGGTTTGTGGACCATGAGCTTGGTTCACAGACGACGGTTTTAGCTGGTACTATGGGG
TATCTTGCACCGGAGTGTGTGACAGATGGTAAGGCTAGTAAGGAATCAGATGTTTATAG
TTTTGGAGTAGTGGCTCTTGAGATTGCGTGCGGGCGGCGGCCGGTTGAGTCGAGGGCG
GAACCGGATCAGGTGAGGCTTGTAGAGTGGGTGTGGGAGTCCTATGGCAGAGGAGAA
GTCTTAAAAACTGCAGACAAGAGACTGGAAATGGAGTTTGATGAGCAGCAAATGGAA
GCTTTGATGGTTGTGGGACTATGGTGCTGCCACCCTGACTTCAAGTTGAGGCCTTCCAT
AAGACAGGTGATTAATGCTCTAAATTTTGAAGCTTCATTGCCGACGCTGCCCGCAAAGT
TGCCTGTGCCAATGTACTTTGCACCATCGATGAATTTATGCAACTTCTCCTACACATCGT
CGGGAACGCCGGTGGATAGAAGTCAGTGTTCATGTAGTAACTGTTCGACTTACACCAC
GCAATCTTCAGGATCAGGATCAAGTGTGTCGCTTTTGAAATCCCAAAGACATGAGTAA
> gene:Csa_6G338050
ATGTCAATGAACTTATCTCTTCCGATCATCATCTTGATCTTCTTCTTGTTTCTCTCCCATT
TCCCCTTTCTTTCCCTTTCTTTTCTCCTTAATCAGAACATCACCCTCTCCGGTGATGCCC
ATCTCAGAAACAACGCCATTTTTCTCACTCAAGAACGCGACTGCCTTTCCCTTTCTCCA
AACTCCTCTTCCTCTGCCTCCGGCTTCGGCTCTGCCGTCTACGTCAATCCCGTTCGCTTT
CTTGATTCCTCCACCAATTCTTCTGCTTCTTTCTCCTCTCGTTTCTCCCTCTCCATCCTCC
CCACCCCTCTCTGTTCTTTCCCCGATGGTTTCGCCTTCCTCATTGCCTCCGATCCCGAAT
CTTTTACCCTCTCCAACAGCCATATACCCCTTCCCAACCCCTCTCATAGCTCCCCTTTCT
CCTTCATCGCTGTCGAATTCGATACCAACTTCGACTCTAATCTTGGCGACATTAATGACA
ACCATCTCGGTCTCAATGTCAATTCGCCCACCTCTTTGACCTCTGTGGATTTTCGATCTC
ATGGCATTGTTCTCAAAAATGGCAGAAAGATTACTGCTTGGATTGAGTATCGAGATGAC
TCCAAAACGATTCGGGTTTGGGTTGGTTATTCGCAAACGAGACCTGTGAATCCTCTTTT
AGCTGCACCGATGGATCTTTCTAAGCAATTTAAGGAGTTTATGTATGTGGGATTCTCTGC

TTCCAATGGGCAGGGCTCTGCTCTTTTCATTGTTGATCGTTGGCAGTTCCGAACTTTCG
GGTTGCTTCCTTCTTTGAGTCCTGTTGATACCATTAATGAAGGAGCTGGTTGTTTTATGT
GTTCGTCGGAGGATTTGAATTCTGATAACAGTCGTTTTGTTGATGCTAGTGAACGGAGG
AAGAAGAGTGGCGAAATGAGTCTTGTATTTGGAGGTTTGGCTGCATTTGCTTGTTCTGG
TGCTTTAATACTTGGTTTTGTTAGTTATACTTTGATAAAGAAGTTGAGAAGTAGAGTGTG
TAGAGGAAGAGAGATTGATAGAACTTGTCTTGTTAAGATGAATAGAATTCCTACAAGAT
TGTCTCTTGGCGAAATAAAGTTAGCTACAATGGGGTTTAATCAAAACAGAGTTGTTGG
GGAAGGTGGTTCTGCTACTGTTTACAAAGGTTCTCTTCCTTCAGGTGTAGAAGTGGCT
GTTAAACGTTTCGAGCAAGGTATGGCGAATAACCGTCTACCCAATCCTTTCGCAACGG
AGTTTGCAACGATGGTGGGATGTTTACGACACAAGAATTTAGTTCAGCTTCATGGATGG
TGTTGTGAAGCAAATGAATTAGTGCTTGTTTATGAGTACTTAGCCAATGGAAGCCTTGC
CAAACTTCTTCACGAGACGTCTCCCAATTCACAATTTGTAATTCCATGGAAGAAAAGG
GTTTCCATAGTTCTTGGAGTTGCTTCTGCATTGACATATCTTCATGAAGAATGTGAGAG
ACAAATTATACACAGAGATGTCAAAACTTGCAACATTCTGCTTGATGCAGACTTGAAC
GCGAAACTCGGTGATTTCGGTTTAGCAGAAGTGTACGAACACAGTTCGTTGACAAGAA
TAGCAACCATTCCAGCTGGAACAATGGGGTATCTGGCACCCGAATACCTTTACTACGGC
GTTCCCACCGTGAAAACCGATGTTTACAGCTTTGGTGTGGTGATCCTGGAAGTTGCATC
GGGTAAGAGGCCCGTAGATGAAGGCGGGATGGTGCTTGTGGATTGGATTTGGGTTCTA
TGGGGAGTGAGGAGCCTGATAGAAGCAGCTGATCCAAGGCTGATGGGAAATTACGATG
TGGTAGAAATGGAGAGAATGTTAATGGTGGGACTTTTTTGTGTACATCCAAATAATGAG
AAGAGGCCAACTGTGAAAGAAGCAGTAAGAATTCTGAGAGGGGAAGCACCACTTCCG
GTACTGCCATTAAGAAAACCAATGGTGGGTATTAGGCCTATTTTATCTGATGATTTTGAA
GACTTAGAGAATCCTTGTTCGGATTATATCGCTTTCGAGGAACCGGCGTGGATGACTCC
CAAAAGTGAGTTTGGTTAG
> gene:Csa_3G115090
ATGGCCATGACATCTTATCTATTCTTATTCTTCATTCTCTTTCTTTTTCTTTCTCCTGAAAT
TCATTCCATTTCTTTCCAAATCAACAACTTCAATTCCAATTCCAACATAGTTTATGATGG
AGATGCAAAACCTATAAATGAGTCCATTACATTCAATGGTGATCTTGGTTGGGCCATTTA
CACCCAAGACCTCCTCCTTTGTGATCATACCAATTTCAAAACCCACTTCTCATTTCTCAT
GAAAAACAACAACAATAATAATAGCAATGGAGGATTGGCTTTTTTCCTTGCTCCATTTG
AATTCAGCCCACCATTTAATTCTTCTCCTCCCTTTCTTGGTCTATATAATTCCACTCAATT
AATCCAACCATCTCAATCCCAAATCCTCCATGTAGAATTTGACACTTTCCCAAACCCTG
AATGGGATCCCCCATTCAAACACATTGGCATCAACAAGAACTCAATTTCTTCATCAATT
TATTCTCCATGGAATTCAACAAATCAAAAGACCCTTGTGTGGATTTCTTACAATTCAAC
TGCTAAAAATCTCAGTGTCTCATTCAACAACAATATTTACACAACTCTTTCTCTTCAAAT
AGATCTGATGGAGATTCTTCCTGAAAAAGTGACAATCGGGTTTTCAGCTGCTTTGGTTG
AGGATCTAAGCATTGAATATTGGGAATTCTCAAGTAATTTAGATGGAAATTATGAAAATG
ATAGTGAAAAATCCACAGATATGAATTTATTAGCAGTTCTAATAGCTTGGGTTGGTGTTT
TTGTAATAGCAATCGTTTCCATAATTATAATTTCTTTTATCAGAAAGAAGAAGAAAGATG
ATTATGAAGAACATGGAGTGATGAAATTGGCTTCAATTTATTCTGATTTGAACAAAGAA
GAAGCTTTAAAACCAAGGAGATTCTCTTACACATATCTTGCTATGGCGACTGATAATTTT
GCAAAGAAAAGAAAATTGGGAGAAGGAGGGTTTGGGGAAGTGTTTGAAGCTCATTTG
CCTGGTGCTAATAAAACAGTTGCTGTCAAGAAGATTTTCAAAAGCTCAAGACAGGTAA
AAAGAGAGTACGTTAGTGAAGTCAAGATTATAAATGGGATGAAGCACAAGAATTTGGT

GCAACTTATTGGGTGGTGCCATGAGGGTGATGATAGTGAATTTCTTCTTGTCTATGAGTT
CATGCCAAATGGTACTCTCCATTCTCATCTTTTTGGAGATCTCCCTCCTCTCTCGTGGCC
AATCAGGTACAAAATATCCTTAGGACTGGCATCAGCACTACTATACTTCCACGAAGAGC
GGGAGAATAGTGTTGTCCACAGAGATATCAAATCAAGCAATGTCCTACTAGATTCCAGT
TTCACTGCAAAGCTCAGCGACTTCGGATTAGCACGGCTCGCAAAGCACGAACTCAACT
CGAAAAGACCCAAGTTAGTAGGAACGTTTGGATACATGGCTCCCGAGTACATAAGCAG
CGGTCGGGCGAGCAAAGAGTCAGACATATTCAGCTATGGAGTTGTTTTACTTGAGATTG
TGAGTGGAAAAAAGTGTTGTGATCATTCAGGAAAGGGGCTGATAGAGTTGGTTTGGGA
TGCTTATGGAAGAGGAGAGTTAGTTAAAGCAATATTGGATAAAAAACTTGGAGTGGAA
TTTGTGGAGGCAAGAGAAGTTGAGCGTTTGAGTATGGTTGGACTGTGGTGTGTTCATC
CTGATTCAACTCAAAGGCCATCCATTAAGCAAGTGATTCAAGTTCTTAGTTTTCAAGAG
GCGATGCCTAATTTACCACTGGAAATGCCTCTTCCAACTTTTAATCATGCTTCCAGGATT
TATAAACTCAACGTTGCATATCCAGAGGAAAATTGGAGCATGACTTGCAGTTTAAAGCC
TTTAAGAACATAG
> gene:Csa_G048440
ATGACTCCCAAAATTATCCTTCCTCTCACTCTTTTTCTCCTTATCTTCACTACCCCAAGA
ACAGAGTGTATCGGCTTCGATTTTACTTCTTTCAATATCAGAAACCTGACTCTCCTTGGC
GATTCCCATCTCCGAGATGGCGTCATTGGTCTCACTAAAGAGCTCGGAGTTCCTTCTTC
AAGCGCCGGCACCGTAATTTACAACAAACCCATCGGATTTTACGACGCCGATGCTAACT
TTACTGCTTCTTTCTCTACAAGATTCTCTTTCTCCATCACCAACATCAACCCCACTTCCT
CCGGAGACGGTCTATCCTTTTTCCTCTCCCCCGATAATCAGACTCTCGGCAGCCCAGGC
GGGTATTTGGGTCTCGTCAATTCTTCCCAATTGACCAAGAACAAGTTTCTCGCCGTTGA
ATTTGATACACGGCTTGATTCTGTCTTTAAGGATCCGAATGCTCATCATGTTGGCTTCGA
CATTGAGAGCTTGATTTCGATCAAGACAGCTAATCCTGCTAGTCAAGGTGTTAATCTCA
AGAGTGGGAAGTCGATTACGGCTTGGATTGAGTACAAGAATGAGGAATGTAGGTTGAG
GGTTTTCTTGAGTAATTCTAGTTTGAAACCTTCTAAGGCGCTTCTTGACGTCGGTATCGA
TCTTTCGAGCTATCTTAAGGAGGTTATGTTTGTTGGGTTTTCGGGGTCGACTGAAGGGA
GTACTGAGCTTCATTTGATTGAGAATTGGACTTTTAATACTTCTGGGTTCGTTTCTGCTA
GGCCTAGATTTAACCCTCATAATGTTTCTGATAGCTCTGTGATTGTTTCTCCTAATATTTC
TTTATCTGATTCTGGCAACGGTCGTCACAGTAGGCTCGGATTAGGTCTTGGGATCGCCG
GACCGGCTTTTTTTTGTGCTGTGATTGCTGTTTTTGGTTTCTTTTCACTTATGAAATGGA
GGAGGATTAGGACTCAGAAAAGCATCAAGGCAGAGCTTTTGACAGGTCCTAGAGAGT
TTAGTTACAAAGAACTAAAGACAGCCACAAAAGGATTTCATTCAAGTAGGATCATTGG
CAATGGGGCTTTTGGGACTGTCTATAAAGCTTTTTGCATCTCATCCGGAAACATTTCTG
CGGTTAAAAGATCGAAGCATTCCCACGAAGGGAAGACCGAGTTCCTTGCCGAGTTGTC
GATCATAGCTCGGTTGAGGCACAAGAATTTGGTTCAGCTCCAAGGTTGGTGTGTTGAG
AAAGGTGAACTGCTTCTTGTTTACGACTTTATGCCGAACGGGAGCCTCGATAAGCTGCT
TTACCAGGAATCTAGTGAAGCTAGCTTGTTAAACTGGTCTCACAGGTACAATATTGCTG
TTGGATTGGCGTCTGTGCTGACATATCTACATCAGGAATGTGAGCAGCAAGTCATCCAC
AGAGATATAAAGACGGGTAACGTACTACTCGATGGGAACTTCAATGCGAGATTGGGCG
ACTTTGGATTGGCAAAGCTCATGGATCATGACAAGAGCCCGGTTTCAACTCTAACGGC
AGGAACAATGGGATATCTTGCACCTGAGTATCTTCAATATGGGAAAGCAACTGAGAAA
ACTGACGTGTTCAGCTACGGTGTGGTTATCCTTGAAGTAGCGTGTGGGAGGAGGCCGA
TTGAAAGAGAACCAGGTACTCAAAAGATGGTCAACCTGGTAGATTGGGTTTGGGGATT

GCATTCTCAAGGCAAGATCATTGAAGCAGCTGATAGCAGATTGAATGGGGAGTTCAAG
GAAGACGAAATGAAGAAGTTGTTACTTGTTGGATTAAGCTGTGCAAACCCAGATAGCT
CGACAAGACCTTCAATGAGGAAGGTCCTACAGATTCTCAACAACGAGGCAGAGCCAG
CGTTAGTGCCTAAAATGAAGCCTAGTCTTACATTTTCTTGTGGCTTTTCACTGACCGTG
GATGACATTATAATAGAGGAAGAAGGTGGAGGAGAGTGGGAAACTTCAAGGCCAACC
ATTGTCCAAATAGACTGA
> gene:Csa_3G734040
ATGGCCAACTTCTGTTTCTTTTCTTTTTTAGCCATTTTCTTCTTTACTGTCCCGGCCGCAT
CTCACAACTTCTTCTACGCCGGTTTTCGCGATCCCACCGCAGCCACCAACTTAACCCTA
CCGGATATCGCCAAAATCGAAGAGAACGGACTCTTAACGCTTACAGACGATGGCGGAT
TTCAACAGGGCCATGTGTTCTACGAATCCCCTGTTCGCTTTAGAAACTCTTCCAACGCC
GATCCTTTTTCCTTCTCTACCAATTTTGTTTTCGCCATTGTCAACGAATTCCCGAATCTC
GGTGGCCATGGCCTCGCCTTCACCATTGCGCCCTCCAAAAACCTCCATGCGCTTCCGGT
ACAGTTTTTAGGCCTTCTCAATTCGACCAATCACGGAGACCCCTCGAACCATTTATTTG
CCGTTGAGTTCGACACCTTCAAAAATGCAGAGTTTGAGGACATCGACGACAACCACAT
TGGAATTGATCTCAATAGTTTGATCTCCAGTGCCTCCACTACAGCCTCTTATTTCATTAA
CGACGGGAACACCAAGCAATTTATAAACCTAAAAAGTGGGGCACCAATTCAAGTTTGG
ATTGATTATGATGCCCCTGTAAATTCTCTCACGGTGGCTCTTTCTCCGTTTTCCACCAAA
CCTCAGAAGCCAATTCTGTCGTTTAATGTCGACCTCTCTCCAATTCTTTTCGAGTTCATG
TACATTGGGTTCTCAGCCTCCACGGGCCAAATGTCAAGTTCTCATTATATTTTAGGTTGG
AGTTTCAGTACGGATGGACCATCCCAGTCACTGAATATTGATTCTCTGCCTTCGGTTCC
CGGGCCGAAGAATACATACTCTGATTTAGCGATTGGGATTTCAATTCTCATCATTTTGAT
TGTCATAGCAGGAATCTTTCTTACTCTTTACAAAATAAAAAAGAAGATAGACATAATCG
AAGCCTGGGAGCTTGAAATTGGTCCGCATCGGTACCCTTACAAACAACTGGAACTAGC
AACCAAACAGTTTAGCAACAGAGAGCTACTAGGACGTGGCGGATTTGGAAAAGTTTA
CAGAGGAACATTGCCAAATTCAAAAACCCGCGTGGCTGTGAAGCGAATTTCCCACGAA
TCCAAGCAGGGCCTCCGTGAATTTATGTCGGAAATCACAATCATCGGTCGGCTTCGTCA
CCGGAACTTAGTTCAATTACTGGGATGGTGTCGTCATGGCGGAAATGAAGATCTGCTTC
TTGTTTACGAGTTTATGGTAAATGGGAGTTTGGATAGTTACATATTCGGCAAACCAAAA
GTGATTTTGAGTTGGGAACAAAGGTTTAAGATCATAAACGGCGTTGCTTCCGGCCTTCT
ATACTTGCACGAAGGATACGAACAGGTGGTCATTCATCGAGACGTGAAGGCCAGCAAT
GTTTTACTCGACGACGAAATGAACGGAAAACTAAGCGATTTCGGCCTCGCCAAACTCT
ATGAACACGGCGAGAATCCGACAACAACACGGGTCGTCGGAACTGTCGGCTACCTAG
CGCCGGAGCTGCACAGAACCGGAAAAGCCACAACCAGCTCTGATGTTTACGCGTTCG
GCGCACTTGTGCTTGAAGTGGCCTGCGGCCGCCGGCCAATCGGCCCGAGAGAGGTGC
CGGAGGAGATAGTGCTGGTGGACTGGGTTTGGGAGAAATACAAAGAGAAGAAATTATT
GGAAGTAATGGACGAGAAATTGAAGGGGGATTTCAATGAGGTCGAGGCAGTGATGATT
TTGAAATTGGGGTTGCTGTGTTCGAAGGATTCGGCAGCGGCGCGGCCGAGTATGCGTC
TTGTGATGCGGTGTTTGGACGGTGAGATTGGAGTTCCCGACGAGATTACGGGTCCGAG
AATGGTGGAAGGAGCCGATGAATTTGTGGATTCGTGGAGTGATAACAGAGACATCACT
TCTGCTTCTTTATCCACTTCGTCTCTTTCGATTCTCGACGGTAGAAGTTGA
> gene:Csa7G048050
ATGTCTTCCTCTTCTTCTTCATCATCCTTTCTTTTCTGCGCCGTAATCTCGATTTTATTATT
CATTTCCACCTCTGTTTTTGCTACAGAGTTTGTCTTCAACACCAATTTCACTTCCACTAA

TACTTTGCTCTTCGGCAACGCCACCATTGATTCTTCCGTTCTTATTCTCACTAGAGATTC
CCCTTTCACCATCGGCCGTGCTCTTTACCCTTTCAAAGTTCCCATCCATTTCTCCAATTC
TTTATTTTCCTTTGCATCTTCTTTCATTTTCTCTGTTGCCCCTCAACCAAATCTTTTCCCT
GGCCATGGATTTGCTTTTCTCTTCACACCCTTTACTGGAATCAATGGAACTAGCTCAGC
TCAGAATTTGGGGCTTTTCAATTTCACCAATAATGGGAGCCCCAGTAACCATGTCTTTG
CTGTTGAGTTCGATTCGTTTCAAAATCTGGAGTTCAACGACACCAATGATAACCATGTG
GGTGTGGATTTGAATTCTCTTGAATCTAATGCTTCCTTTGCCGCTGGGTTTTGGAGTGG
ACCTGATGATGGAGAATTCAAGGAATTGAAGATCAATAATGGAGAAACTTATCAGGTTT
GGATTGAATGTCTTGATTCTTTAGTTAATATCACAATGGCTGAAGTGGGAATGAAAAGG
CCTAGAAAACCATTGATATCTCTGTTTGTTGATTTCTCTGGATTGCTTTTGGATGAGATG
TATGTAGGATTTACAGCAGCCACTGGGCAATTAGTTCAAAGCCATAGGATATTATCTTGG
AGTTTTAGTACCTCCAATTTGTCTATAGGTGATGCTTTATTGATAACAGATTTGCCATCAT
TTGTTCCTCAAAAAGAGGGCACCATTTTTAACTCAAGAGCTTTCATATTGGGAATAACT
TTTGGGGGTGTTGGACTTGTGATTATCTGTTTTATAATCTGTGGTGTTGTAATTATCAAA
AGAAGGGGAAGAAAAAAACCAAAGGATGATGAAATTGAAGATTGGGAGTTAGAATAT
TGGCCGCATAGGTTTGCTTATGAAGATGTTTATGAAGCAACTGGCGGGTTCTCAGAAGC
CAATGTAATTGGATCTGGGAGAAACGGGAAGGTCTATAAAGGGACATTAGGGAGATCA
AAAGTTGCTGTGAAGAGAATCTCTGTTGAAGCTGAGAGTGGAATGAGGGAATTTGTAG
CTGAGATTTCAAGTTTAGGAAGATTAAAGCATAGAAATCTAGTTAAGCTTATAGGATGG
TGCAAAAAGGAGAAAGGAAGCTTAATCTTAATGTATGATTACATGGAAAATGGAAGTTT
AGACAAGAAACTATTCGAGTGTAACGAGAATGAAAGGCTGAGTTGGGAGAAAAGAAT
GAAAATTTTAAAAGATGTAGCCACGGGGTTGCTATATCTACACCAAGGTTGGGATTCCA
GGGTTCTGCATAGAGACATAAAAGGAAACAACGTGCTACTTGACAAGGACATGAATGC
AAGGTTGGGAGATTTTGGGTTGGCTCGAATGCAACCCCATGAAAAAACGGCAGACAC
AACTCGTGTAATGGGGACTGTCGGGTATATGGCACCAGAGGTGGTTCGAACAGGGAGA
GTATCAGCACAAGCAGACGTTTTTGGGTTCGGAGTGTTGGTTTTGGAGGTGGTTTGTG
GGAGGAGAGCAGTAGAAGAAGGGAAGCCGTGGTTGATAGATTGGGTAAAAGGATTAA
TGGAGAGGAATGAGATTGGATTAGCAGTTGATGAGAGATTGAGAGTTGAGGTAATAAG
TGGGAATGAGATTGATGAAATGGAGAGAATGGTTTGTTTGGGATTATTATGTGCACATA
ATGAAGCTGGTGCAAGGCCAACAATGCAACAAGTTGTAAATATTTTGTGTGAGAGAAA
TGGAAGTGGTTCAAATGATGGATTGTTAAATAGACTAAGATCAACAAGAATACTCTCTG
AGATTTCTCAAGGCAAGAATTTTCAGCAAAATCATCCAACGTTTGAAGAAATCAAAAC
GTCTTCATCTTCAACATCATTCATTGAATCTGATATTTTAAAGAATGACCGGTAG
> gene:Csa7G067410
ATGGCTGCACAAAATAATCTTCTCATTCATTTGTTCTTTCTTGTTCCATTTGCAACTTCTT
TATCTTTCAATTTCACCTCTTTCAATCAAGGTAATGCCGACATGATCTACGACAGAACAT
TCCCAACAAACCAAGTAATTGAGCTCACTGGAGACTCATCAAACAACAATATGAATTT
TGTTGGACGAGCTACGTATTCGCAACCATTGCATCTTTGGGATGAAGGCTCTGGAAACA
TGTCAAGTTTCCAAACTCATTTCTCCTTTGCCATCAATTCAAGAGGAAGAGCTAACTAT
GGTGATGGCCTTACCTTCTTCTTTGCTCCCAATGGTTCTATCCTCCAGGCCAACATCTCA
AGAGGCAGTGGCTTAGGTATTGGCTATGATCCCGAGTTATGGAATGGGACTGCCACGTT
TTTTGCAGTTGAGTTTGATATCTACAGCAACAATTTTGATCCACCTTTCGAACACGTAG
GTATCGATATTAATTCGATGAAATCTATTGCGTATTCGAGATGGAAATGTGATATCATGGG
AGGGAAGAGGAATGATGTTTGGATTAACTATGATTCCGATACTCATAATTTGAGTGTTGT

TTTTAGTGGGTTTGAGAATAATAATACTCTGTTGCAACACCTTCACCATGTTGTAGATTT
GAGGTTGAATCTACCTGAATGGGTTACTTTTGGTTTTTCTGCTTCTACTGGATATGAATA
TGCAACTCACTCTGTATATTCTTGGTCTTTTCACTCAACCCTTGAGTTGACCCTTGAACC
AACCTTCACTACTGATCCCAACTCGGTTGCAAGTGCCCCAAGTCCTGGCCCAAGCTTG
CCTCCAAACAATAATGATGGTAGCACTAGCAAGACAGGTCTAGAAATTGGATTGGGCA
TTGCTGGAGGAGTTATCTTTGTGGGTGGGTTAGTGATCGTTTGGATTATTGTTTGGAAG
AAGATGGCAGCGATGAAGAACATAGAGGAGGAGATTATGTTGGATGACTCCGAGTTCG
AGAAGGGTAAAGGACCTCGACGGTTTTTGTATAAGGAATTGGCTCGAGCAACGAACA
ACTTTAAGGAGGATAAGAAGCTTGGCGAAGGAGGATTTGGTGGGGTTTATAAAGGTTT
CTTAAGGGAACTGAACTGCAATGTTGCTGTGAAGAGAATATCAAAAGGGTCTAAACAA
GGGATAAAGGAGTATGCATCTGAGGTGAAAATCATAAGCCAATTAAGGCATAGAAACT
TGGTTCAACTAATTGGTTGGTGTCATGAGAAAGACGAACTTCTATTGGTTTATGAATTC
ATGCCAAATGGAAGCTTAGATACCCATCTTTTCAAACCAAACAATTTCCTGACATGGGA
GTTAAGGTACAAAATCGGTCAAGGAATTGCCTCTGCTTTACTGTATCTTCATGAAGAAT
GGGAAATGTGTGTGCTGCATAGAGATATAAAGTCAAGTAATGTGATGTTGGATTTGAAC
TACAATGCAAAGCTTGGAGATTTTGGTTGGCTAGGTTAGTGAACCATGGCAAAGGTTC
ACAAACAACTGCTCTTGCTGGGACACTCGGTTATTTGGCTCCTGAATGTGCAACAACA
GGAAGGGCTACAAAGGAAACAGATGTGTATAGTTTTGGGATTGTTGCTTTGGAAATTG
CTTGTGGAAGAATGCCATTCAATCCCAATGTAGAGGAAGAGAAGATGGTTATGGTTGA
ATGGGTTTGGAAGCTTTATGGCTGTGGGAAGGTTCTAGATGCAATTGACTCAAAACTTA
GAAAAGAAATTAGATCATTTGGAGATGAGGAGAAGATGATGGAGTGTTTGATGGTGGT
GGGTCTATGGTGTGCTCATCCAGATAGCAATGCTAGGCCTTCAATAAGGCAAGCCATTA
ATGTGTTGAACTTTGAAGCACCATTGCCAATTCTTCCTTCTCACTTGCCAGCACCCACT
TATGATTTTAGACCAATTGCTTCTTCATCTACTTCATCTTCAACAACTCAAAGTGGCGTT
GCTTCTCTTGCTAGTAATAGTTCCAACTTATAG
> gene:Csa1G004050
ATGGAACCACGACGTCGACGCCGTCCTCCCTTACTTCTACTTCTACTTCAGCTTTGTATC
TTCTTTCTCTTCTTCTTTTCTTCTTATGCAGTCGATTTCTTCTTCAATTCCTTCTCAAACG
AATCGAATGGCACTGATTTCATTCTCATTAGCGACGCCAGGTTCGATTCCCCTGTAATCC
GCCTCAACAACGACTCAAACCAGAATTCAATCGGTCGTGTTTTCTATCACACGAAGCT
AACTATGAAACCCACTTCGAATTTCTCAAAGATTTCTTCCTTTTCAACTTCTTTTGTGTT
CTCGATCTTGCTGGAAGACGCTACTAGTCCAGGCTTCGGTTTGGCCTTCGTTCTTTCCA
ATTCTACTTCCCCTCCCGGTGTCATCGCCGGCCAATACTTTGGTCTCTTTACTAATTCAA
CAACCCACGTCGTTGCTCCACTTCTCGCCGTCGAATTCGACACTGGCCGAAACACGGA
GTTCAACGACCCAGATGATAGTCACATCGGAATCGATTTGAACAGTGTGTTGTCGAGTA
AAATACACGGCGCTGGGTATTTTGGTTCCAATGGGCAATTTATTCCGATTCAGATGAGA
AATGGGCAAAACCTTCGCGCTTGGATCGAATTCGATGGAGCAAATTTCGAAATTAATGT
CACAATCGCCCCAATTGGAATTCCTCGTCCTTCGATACCCACTTTGAGTTTTAGAGATC
CTTTAATTGCAAATTATGTATCGACTGAAATGTTTGTGGGATTTTCAGCATCGAAGACAA
AATGGGTTGAGGCGCAAAGGATTTTAGCTTGGAGTTTTAGTGACACTGGGGTTGCTAG
AGACATTAACACCACAAATTTCCCTGTGTTTATGAGAGAATCACCACCATCTCCTTTGT
CGCCAGGATCGGTTGCCGGTATTGTAATTGGCTGTGTTCTGTTTGTGATCATAGTTTCTG
GGTTTGGATTTTTTTGGTACTGGCGAAAGAACAGAGCAAAAGAAGAAGACGAAGAAA
TTGAGGATTGGGAACTTGAATATTGGCCTCATAGATTCTCCAACGAGGAGCTTTCACAA

GCTACTGATAAATTTTCCGAAGAAAATCTGTTGGGATCCGGCGGATTTGGAAGGGTATT
CAAAGGAACTTTACCAAACCACACGGAAATCGCCGTTAAATGTGTAAATCACGATTCA
AAACAGGGGTTAAAGGAATTCATGGCAGAAATCTCAAGTATGGGTAGGCTTCAACATA
AGAATCTAGTTCAAATGCGAGGATGGTGTCGAAAAAAAAATGAATTAATGTTGGTTTAT
GATTATATGCCGAATGGAAGTCTTAATCGGTGGATATTTGACAAGCCCACAACGTTACT
CAGCTGGAAGCAGCGACGCCGTGTACTCGGCGACGTTGCGGAAGGTTTGAACTATCTC
CATCATGGTTGGGATCAGGTGGTGATTCACAGAGACATAAAATCGAGCAACGTGTTGTT
AGATTCAGAAATGAGAGGCAGAGTCGGCGATTTTGGGCTGGCGAAGTTATACCAACAC
GGCGAAACTCCGAACACCACTCGAATTGTTGGGACTTTGGGGTATTTGGCGCCAGAGA
TTGCGACGGTGGCTACTCCAACAGCGGCTAGCGATGTTTATAGTTTTGGGGTTGTGGTT
CTTGAAGTTGTGTGTGGGCGACGGCCGATTGAGTTGGCAGCGGAGGAAGAAGAAATG
GTGCTTATTGATTGGGTGAGAGATCTTTACTCTGCCGGCCGGTTGATTGCGGCGGCAGA
CTCGAGAATCAGAGAAGAGTATGAGACTGAAGAGATTGAATTGATGTTAAAGCTTGGT
TTAGCTTGTTGTCATCCGAACCCTGAACGCCGGCCTACAATGAGAGAGGTGGTGGCAG
TTTTGATCGGAGAACAGCCATCAGCGGAGTCGGTTGAGCTTTTATCAGGATTGGCCGG
TGGCATGGTTGACGACACTCGAAATGTTGATATTTGA
> gene:Csa3G734030
ATGGCCCGTCTCTGTTTACTCTGTTTCTTCTTCTTCCTCTTTCTCGCCGCCCCCGCCGCA
TCTCAGCAGCTTTACTTCTCTGGATTTCAAGATGATGCCGCCGTCGCTGCCAACTTAAC
TCTCACTGACATCGCCAAGATCGAGCAGAACGGAATCTTGAAACTGACAAATGATACC
AGTCGATTACAGGGTCATGCCTTTTACAGTTCCCCCGTTCGTTTCAAAAACTCATCCGA
CGGTAAAGTTTTTTCGTTCTCCACCGCTTTTGTCATCGCCGTCGTCCCTGAGTACCCGA
CTCTAGGTGGCCATGGCCTCGCCTTCACCATTGCCCCCTCGAAGAACCTCCGTGGACTT
CCCAGTCAGTATTTAGGCCTTCTCAATGCGAAAGACGTCGGAAACTTTACGAACCATCT
CTTCGCGGTTGAGTTCGATACTGTTCAAGATTTCGAGTTTGCCGATATCAACGATAACC
ACGTCGGAATTAATCTCAACCATATGATTTCTAATGTCTCCACCACCGCTTCCTATTTCG
TCGACGATGGACCCACCAAGCAGAACCTAACCTTGAAAAGTGGGAGGCCGATTCAAG
CTTGGGTCGATTACGATTCCTCTGTGAATTCCCTCACGGTGGCACTTTCTCCGTCTTCCA
CCAAACCCAAGAAGCCGATTCTGTCATTTAATGTAGATCTCTCTCCGATTCTTGACGAG
TTTATGTATGTTGGGTTCTCGGCCTCCACTGGGCTTCTTGCAAGTTCGCACTATGTGTTG
GGGTGGAGTTTCAGTATGAATGGACAAGCCCGGTCTCTGGATCTGTCTTCTTTGCCTTC
TGTTCCAGGTCCGAAGAAAAAGCACACTGCTTTTACAATTGGGATTTCTGTTGCGGCT
GTTTTGATTGTGATAATCGCGATTTGTGTTGCTGTTTTAATCATTTGGAAGATAAAAAAC
GCTGATATAATCGAGGCCTGGGAGCACGAAATCGGTCCTCATCGGTACTCTTACAAGGA
GCTCAAGAAAGCCACTAAGCGATTTAGGGATAAAGAGCTTCTAGGGCGCGGTGGGTTT
GGGAAAGTTTACAAGGGAACTTTGCCGAATTCAAAAATCCAAGTGGCTGTGAAGCGA
ATTTCTCACGAATCGAAACAGGGCCTAAGGGAATTTGTGTCGGAAATCGCTAGCATTG
GCCGGCTTCGTCACCGGAACTTGGTTCAGTTACTGGGATGGTGTCGCCGCCGTGGCGA
TCTGCTTCTCGTTTATGATTTTATGGCTAATGGGAGCTTGGATAACTACATTTTCGACGA
CCCGGATGTGAATTTGAGCTGGCAACAGAGATTCGGAATCATTAAGGGTGTCGCTTCC
GGTTTGCTCTACTTGCACGAAGGCTACGAGCAGGTGGTGATTCATAGAGATGTGAAAG
CCAGTAATGTCCTGCTCGACAGCGAGATGAATGGAAAGCTCGGCGATTTCGGCCTCGC
TCGGCTATATGAACACGGGGCCAACCCGAGCACGACTCGGGTCGTCGGAACTCTTGGA
TATCTGGCACCGGAGCTTCCCAGAACCGGAAAAGCCACCACCAGCTCAGATGTGTATG

CCTTCGGCGCACTTCTGCTAGAAGTGGCCTGCGGGCGGCGGCCAATCGACCCGAAATC
TTCGTCAGAAGAGCTGGTGCTGGTGGATTGGGTGTGGGAGAATTACAGGGAAGGGAA
ATTGCTGGACGTAATGGATCCAAAATTGAAAGGGGATTTCAATGTAGTTGAGGCAATGA
TGATATTGAAACTGGGGCTGTTTTGTTCGAACGATTCCGCGGCGGCACGACCGAGTAT
GAGGCAGGTGGTGAGGTTTCTGGAGGGAGAAATGGGAGTTCCGGAGGAGATATCAGC
GCCGAAAGTGATGGAAGGAGGGAGGAATGGAGAAGGGTTCGATGATTTTGTGAATTC
GTTTGCTTCTTCTTCGTTTAACAAGTTTAGTTCATATTCATCGACTGGTAATAAGGATATG
GATATGAGTTTTGCTTCATTTTCAACTTCTCCTCTTTCGCTTCTCAACGGCAGAGACTAA
> gene:Csa2G439210
ATGGCTAATTTCTTGTATGTCTTTTCAATGCTAAGCCATTTCATCCTGTTTCTCAATCTTC
CTCTCTCTGTTATCTCTGTTTCTTTCAAAATAGACCAATTCAAAAGCGATGATAACACCA
TACTTTATCAAGGAGATGCTGTTGTTCTTGGTGGAGAGATTTTACTAAGTGATCCTGAAT
TTTCTTGTCATGTTGGTCGTGCTATTTACAAAGATCCAATCCAAATTTGGGATTCTGAAA
CAGCAAAACTCACTGATTTCACAACCCATTTCACCTTCACCATTGATACACAAAAAGTT
CCAGACTATGGCCAAGGCTTTGTTTTTTTCTTGGCTCCATCTGGTTTTCAAATCCCTCCA
AATTCAGCTGGTGGGTTTCTTGGCCTTTACAACAAAACGTACAGCAATTCAGTTACTAA
TCAAATTGTTCATGTTGAGTTTGATACTGGCTCAAATGGCTGGGATCCTCCATATGCACA
CGTGGGTATAAATATTAACTCTGTTACTTCTTCCAATGACACTCGTTGGAATGTTAGCTT
GCACAGTGGGGATTTGGCTGAAGTGTGGATTTCCTATAATTCAACAATCAAACTCTTGA
GTGTATCTTGGAAATACCAAAAGACATCCACTTTATTAGAGAATACCACTCTGAGTTATC
CTATTGACTTAACGACCGTTCTTCCACAACAAGCGACTGTGGGATTCTCAGCCGCAAC
TGGTGCTCATTTAGAGAGACATTCTGTATCTTCCTGGGAATTCAACTCTACTTTGGACAT
GAAGCCAACAAGTATAAGTGCTGGAAACAAAGTTAGTGTAATTGTAGGTGTAACAGTG
TCGGTTGGAGGCTTAATTCTTGTGGGAATTATAGTATTTGTAACACTTTCAAGGTTGAA
GGAAAAGAAAAGGAAAAAAGATCAAGAAAATCTTGAGGAAGTAAATTTGACATCCAT
TAATGATGATTTGGAAAGAGGGGCTGGACCTAGAAGGTTTTCTCACAAGCTTCTTGCTA
TGGCAACAAACAACTTCTCAAATGAAAGAAAGTTAGGGGAAGGAGGATTTGGTGCTG
TGTATAGAGGCTACATACAAGATTTGGATTTGAATATAGCTGTGAAGAAAATCTCAAGG
GGTTCGAGGCAGGGGAGAAAAGAGTATATAACCGAAGTGAAGATCATTAGTCGACTTC
GTCATCGAAATTTGGTGCAACTCATTGGTTGGTGTCATGATAAGGGTGAGTTCTTGTTG
GTTTATGAATTCATGTCTAATGGTAGTCTTGATTCTCATCTCTTTGGTAAGAGAACCCCT
CTTGCTTGGAGTGTGAGATACAAGATTGCGTTAGGTTTGGGCTCCGCTTTGCTGTATCT
TCATGAAGAAGGGGAACAATGCGTGGTTCATAGAGATATCAAATCTAGTAATATCATGT
TGGATTCAAACTTCAATGTCAAGCTTGGAGATTTTGGACTCGCCCGATTAATGGACCAT
GAGTTGGGTGCTCAAACAACTGGGTTGGTAGGAACTTTGGGCTACTTAGCTCCTGAAT
ACATAAACACAGGTAGAGCTAGTAAAGAATCTGATGTGTTCAGTTTTGGAGTTGTTGCT
TTGGAGATTGCCACTGGGAGAGTGTCAAGAACCTCGATGGAAAAGGAATCTCACAAG
GGTTTGGTGGAGTGGGTTTGGGATCTTTATGGGAGTGGACAATTACTTGAGGGTGTGG
ATGCGAAATTACAATCTAATTTTGACAAAAAACAAGTTGAATGTTTGATGGTTGTTGGA
TTATGGAGTGCTTATCCAGACCCTAATTTTAGACCTTCCATAAAACAAGTGATTCAAGTT
CTTAACTTTGAGGCTGCAGTGCCAAATCTTCCAAATAAAATGCCTGTTCCTACATATAAT
GCTCCTTCCACATCAATGAGCTCAAATGAACCTTCCTTTACTGTCAGTCTTGACATGGG
TCGTTGA
