# Supplementary material for: Genome-Wide Identification and Characterization of Lectin Receptor-Like Kinase Gene Family in Cucumber and Expression Profiling Analysis under Different Treatments
Source: Genes (Basel). 2020 Sep 2;11(9):1032. doi: 10.3390/genes11091032 (PMC7564967; doi:10.3390/genes11091032)
Supplement: Supplementary file 1 [file genes-11-01032-s001.zip › genes-891962-supplementary/Additional file5 Table S3.pdf]

C-Type

gene:Csa1G056960
MAPTEVKTLFLSLFVLIWLSRMGVSDTMFNESGIGYSVVSRKEISKGRCPHGWIISPSKTK
CFGFMSSPKSWNDSETQCNSFGGNLAALVTYQEFSYAQNLCNGTLGGCWVGGRAFNSLN
DFVWKWSDNVSKWNDSIFPSATLQSNCKNASCLRNDSVETCTLIFGGPATPFLRDEKCNSS
HPFICMINLDDRCHRMHCHKEYLVILAVVSGLIFCTTLAVVIWLLAHKRSKKRRRSRKPSN
PAASALVPPLWRVFTKEELRSMTKNFSEGNRLLGDAKTGGTYSGLLPDGSRVAIKRLKKSS
FQRKKEFHSEIARVARLRHPNLVALKGCCYDHGDRYIVYEFIVNGPLDRWLHHVPRGGRS
LDWTMRMKIATTLAQGIAFLHDKVKPHVVHRDIRASNVLLDEEFGAHLMGVGLSKLVAY
EVMHERTVMAGGTYGYLAPEFVYRNELTTKSDVYSFGVLLLEIVTGRRPAQAVDSVGWQ
SIFEWATPLVQAHRYLDLLDPHITATSTSEIPEAGIVQKVVDLVYACTQHVPSMRPRMSHVV
HQLQQLAPSPLTTK

G-Type

>gene:Csa5G550210
MFFDAAPLSILLLLPLASAQPTTNPRKFSSFSISQSPWRPTQNLTLLSPNSLFAAGFHPLPNN
SNLFIFSVWYFNISTDNVVWSANRLHPVNRSAALVITATGQLRLNDASGRNLWPSNNVSA
HSNSTQLILRDDGDLIYGTWESFQFPTNTFLPNHTFNGTSIVSNNGKYSFVNSANLTFGTET
YWSSGNPFQNFQIDGQIIINNQIPVIPSDFNSTRFRKLVLDDDGNLRIFSFNPNWPRWDVVW
QAHVELCQILDTCGPNSVCMSSGSYNSTYCVCAPGFSPNPRGGARQGCHRKLNVSNKPK
FLQLDFVNFRGGVKQISLQTPNISVCQADCLKNSSCVGYTFSFDGNGNAHAQCVLQLDIL
SNGLWSPGMKAAAFVKVDNSETDRSNFTGMMYKLQTTCPVRITLRPPPVNKDNTTRNILI
ISTIFVAELITGAVFFWAFLKRFVKYRDMARTLGLESLPAGGPKRFNYAELKTATNDFSTCI
GRGGFGEVFKGELPDKRVVAVKCLKNVAGGDRDFWAEVTIIARMHHLNLLRLWGFCAEK
GWAFEKAFVEEKMKEILDGRIREEYERGGNVCIVNRMVETAMWCLQNQPEKRPSMGKV
VKMLEGKLEIPPPEKPSIYFLSQ
>gene:Csa5G550230
MSLAVAFSALFLLFFPSPAAAQSPKPTNFSAFSISQSPWRPSHNLLLLSPNSLFAAGFRPLPN
NSNLFIFSVWYFNISTDNIVWSANRLHPVTRSAALVITATGQLRLNDASGRNLWPSNNVSA
NSNSTRLILRDDGDLIYGTWESFQFPTNTILPNQTLNGTTIISNNGKYSFVNSVNLTFGTER
YWWTDNPFKNFENTGQINRDNQNPIYPTDFNSTRLRKLVVDDDGNLKILSFNPNSPRWD
MVWQAHVELCQIFRTCGPNSVCMSSGSYNSTYCVCAPGFSPDPRGGARQGCNRKLNVSN
KSKFLQLDFVNFRGGANQIFMETPNISVCQANCLKNSSCVGYTFSFEGNDQCVLQLDILSN
GFWSPGMKTAAFVKVDNSETDQSNFTGMMYKLQTTCPVHISLRPPPDNKDNTTRNIWIIV
TIFIAELISGAVFFCAFLKRFIKYRDMARTLGFESLPAGGPKRFSYDELKIATNDFSNPVGKG
GFGEVFKGELPDKRVIAVKCLKNVSGGDGDFWAEVTVIARMHHLNLLRLWGFCAEKGQR
MLVYEYIPNGSLDKFLFVKSSFSDSIEIDGENPLLDWGIRYRIAIGVARAIAYLHEECLEWV
LHRDIKPENILLDNDFCPKLADFGLSKLKENDGTAVSMSRIRGTPGYVAPELVKLGSNSITP
KADVYSFGMVLLEIISGTRNFDTKEGSTVESAFWYFPSWAFEKAFVEEKIEEVLDSRIRNE
YDSGGHFAIVNRMVQTAMWCLQSQPEMRPSMGKVVKMLEGKLEIPNPEKPSIYFLSEGQ
EGPKHQIAMVVDSVDSMDSDFPPAEYSSTSLSFG
>gene:Csa1G071270
MNSAFPLLLLLSLVTTFFSSKIFAYGSTDTITSTNFIKHPSTIISNADSFQLGWFSPLNSTAQY
VGIWYHQISIQTLVWVANKDTPLNNTSGIFTISNDGNLVVLDEYNTTIWSSNITSPTANTTA
RILDSGNLVLEDPVSGVFIWESFEHPSNLLLPAMKLVTNKRTQQKLQYTSWKTPSDPSKEG
ILEQQFWNQSKGNWEQSWSAFSTECDYYGVCGAFGVCNAKATPVCSCLTGFKPKDEDE
WKRGNWSNGCERITPLQCESSARNNSRVEEDGFLHLETVKVPFLVEWSNSSSSGSDCKQE
CFENCLCNAYAYENGIGCMLWKKELVDVQKFENLGANLYLRLANAELQKINDVKRSENK
GTVIAIVLPTTLVIFIIIVIYFCWRWKANKNEYIKNGKRLKLRKDDMIGDESELKELPLYDFE
KLAIATDSFDLSKKLGQGGFGPVYKGTLLDGQEIAIKRLSRASNQGYEEFINEVIVISKLQH
RNLVQLLGCCIEGEEKMLIYEYMPNSSLDAFIFGKIYRCLSSEFQDFSIFCFRITNHTNHNA
NILHVGSAKQKLLDWRKRFNIINGIARGLLYLHRDSRLRIIHRDLKASNILLDKDMNPKISD
FGMARIFGSNEVEANTIRVVGTYRNYPTNND
>gene:Csa1G605750
MIPPTVSLLLFLTSLLLYAQSNATQIPTGSSLIAGTSSLHPWLSPSNHFAFGFQNLDNDNRYL
LAIWFYKVPENNIVWFAKSDDDDNNNNPVFAPKGSKIQLTASTGLVLRNPNGEEIWKSKPI

TSSISFATLNDTGNFMLVDSINGSVWESFSYPTDTLLPSQKLEVGGVLSSRKSLGNFSLGKF
QFRLLEDGNAVLNTINLPYGYHYDAYYISNTFDPASTQNSGSEVIFDEVGFLYVLKRNGVQ
VNITQFSVGNPVEAFYYKATMNFDGVLTVSSYPKNTNGVVANGSWKDLFRIPDNICLSNE
NPITRLGSGICGFNSICSLKSNGRPSCNCAQGYSFVDPNNEFSNCKPFIAQGCEDEDDKFNQ
NLYEMVDLQYTNWPMYDYERFPTMNEQTCKSSCLEDCFCVLAVFGGRDCWKKRLPLSN
GRQDASITSISFLKLRKDNVSLESFPNGGGAQKKQTTIILVITVLLGSSVLMIILLCFFVLKR
EILGKTCTKNFSLECNPIRFAYMDIYKATNGFKEELGRGSCGIVYKGTTELGDIAVKKLDR
MFEAEREKEFRTEVNAIGQTHHKNLVRLLGYCDEGNNRMLVYQFMSNGSLSTFLFNNDP
KPSWKLRTQIAYEIARGLLYLHEECGTHIIHCDIKPQNILLDDNYNAKISDFGLAKLLKMD
QSRTQTGIRGTKGYVAPDWFRSSPINAKVDVYSYGVLLLEIICCRRNVEMEVGDGAQGER
GVLSDWAYDCYEQGRLDILIEGDTEAIDDIVRVERFVKVAIWCIQEEPSRRPTMENVMLML
AGNLEVSLPPCPYHSFSSIV
>gene:Csa1G071170
MGKLIWRFSVSIFLFFWMTMAFLSRKSLAIDSIKAGESINGNTQILVSAQQKFVLGIFNPKD
SKFGYLGIWYKNIPQTVVWVANRDSPLVDSSARLTLKGQSLVLENESDGILWSPTSSKFLK
DPIAQLLDNGNLVIRESGSEHYVWQSFDYPSDNLLPGMKVGWDLKTRMNWKLTSWKSS
NDPSSGDFTYGMDPAGLPQLETRRGNVTTYRGGPWFGRRFSGTTPFRDTAIHSPRFNYSA
EGAFYSYESAKDLTVRYALSAEGKFEQFYWMDDVNDWYLLYELPGDACDYYGLCGNFG
VCTFSTIPRCDCIHGYQPKSPDDWNKRRWIGGCVIRDNQTCKNGEGFKRISNVKLPDSSG
DLVNVNMSIHDCKAACLSNCSCLAYGMMELSTGGCGCLTWFNKLVDIRILPDNGQDIYV
RLAASELESDKRKLTVVLCLSVASLISFLIFVACFIFWRRRTIKGNEVQSHENEAEMPLYDFS
MLVNATNDFSLSNKIGEGGFGPVYKGVLPCGQEIAVKRQAEGSSQGQTELRNEVLLISKLQ
HRNLVKLLGFCIHQQETLLVYEYMPNKSLDYFLFGWCLT
>gene:Csa1G605730
MACMISHIFLLLPSVVYAQSNSMLNVGGSLIAGDASASPWISPADHFAFGFREVDDGLFLL
CIWYNKIDEKNIVWFAQHDQNPVPKGSKVEVTASNGLLLKSSQGGELWKSGPISSVVAFG
TIYDTGNLVLLDSNTTPLWESFNQPVDTLLPTQKMEVKDFLSSRKSQNTYSLGKFQLRFSE
GNLVLNMRSLPTTYAYEPYHVIQAFEGNQVVFDEDGFLYIIQRNGKRVNISEPESAYPANT
HYYQVTLNFDGVVTVSHHTRNPSAFNATWIHFKTIPNNICVAMRGNLSSGACGYNSICTL
NNDQRPSCNCAPGYSLIDLNDKYSDCKPIIQPICEDGENNSTTDLYRLQDLPNTDWPTQDY
ELFKPFTIEECKNACLLDCFCVAVVYRDNSCWKKKLPLANGRKDSGEKSISFLKLRRNISSI
GQDSNLPRSKGKKNHDTLVLALSILLSSSLLIILVLASFISRGFISHHRKKHTSDFLPRGNFG
SMQKFTFKELREATNGFKEELGRGSCGVVYKGVTEVGSVAVKIFNDMFEDSEKEFKTEVI
VVGEAHHKNIARLHGYCDDGKRCMLVYEFLSNGSLASFLFGDSKLSWDLRTKITYGIARG
LLYLHEECNTEIIHCDIKPQNVLLDEHYNPKISDFGLAKLLKMDQSRNRVETNIKGTTGYIA
PDWFKSTPVTTKVDVYSFGVLMLEIICCRRNGDMEVYEQGREILVDWAYDCYQQGRLDV
LVEGDFEAIDDMGRLERFVVVAIWCIQEDPYQRPTMRQVIPMLEGIVPVSTPPSPCSFSSTS
> gene:Csa1G605740
MACIIPHIFLFLPSIIYAQSDSMLYIGSFLIAGDPSSSPWRSPADEFAFGFKQVEGDLFLLSIW
YNKLDEKSIVWYAIHDQNPAPRGSKLEVTASNGLLLQSSQGGEPWKPSPISGVVAFGKIND
DGNLVLLDSNSNTVWESFKQPANILLPTQTIEVNDLLSSRKSQNSYALGKFQLRLSEGNLV
LNIISLPSTYTYEPYHVIQAYEGNQIVFDKGGFLYIMQKNGTRVNISEPESAYPANTHYYQV
TLNFDGVITVSHHTRNPSAFNATWMDFKKIPHNICVTMRGNYSSGICGYNSICTLNNDQRP
SCKCPPGYSLIDPNNKYSDCKPNIQPTCEGDENNLTNNLYSLRVLPNTNWPTQDYELFWPF

TVEECKNACLLDCFCVVAVYRDNSCWKKKLPLSNGREDNNETSVSYLKLSTSSIGQGFDL
PMPKGKKKPNTLVLVLSTLLGSFVLIVLILVSLICRGYTFDHKKQLMGNFHPRESFGSSMQ
KFTFKELSEATNEFEEELGRGSCGIVYKGTMEIGPIAVKKFHMSEDGEKEFKTEINVLGQT
HHKNIVRLFGYCDDNKIYFLIYEFMSNDNLARFLFSDTKPSWDIRTKITYGIARGLSYLHD
ECNTQIIHCDIKPQNVLLDECYNSKISDFGLAKLPKMDQSRTRIETNIKGTTGYIAPDWFKS
TLVTTKVDVYSFGVLLLDIICCRRNGEDVEVSEEGREILADWAYDCFEQGRLNVLVEGDL
EAIGDKERLERFVKVAIWCIQEDTSRRPTMKEVMYMLEEVVPVSTPPSPCPFNSIC
> gene:Csa1G071160
MAKLISICLFFWTTTALFPRKSLAIDSIKAGESISASAQILVSAQQKFVLGIFNPEGSKFKYL
GIWYKNIPQRTIVWVANRDNPFVSSSAKLTFNEEGNVILVDETDGVLWSSTSSIYVKEPVA
QLLDNGNLVLGESGSENYVWQSFDYVSDTLLPGMKLGRDLKAGMTWKLTSWKNQNDP
SSGDFTYVMDPGGLPQLEIHRGNVTTYRSGPWLGSRFSGGYYLRETAIITPRFVNNSDEAF
YSYESAKNLTVRYTLNAEGYFNLFYWNDDGNYWQSLFKSPGDACDDYRLCGNFGICTFS
VIAICDCIPGFQPKSPDDWEKQGTAGGCVRRDNKTCKNGEGFKRISNVKLPDSSAKNLVK
VNTSIQDCTAACLSDCSCLAYGRMEFSTGDNGCIIWFERLVDMKMLPQYGQDIYVRLAAS
ELESPKRKQLIVGLSVSVASLISFLIFVACFIYWRKRRRVEGNEVEAQEDEVELPLYDFAKIE
TATNYFSFSNKIGEGGFGPVYKGMIPLGQEIAVKRLAEDDKKRSLLSWKKRMDIIIGIARGL
LYLHRDSRLIVIHRDLKVSNILLDNEMNPKISDFGMARMFGEDQTMTQTKRVVGTYFLT
> gene:Csa6G516770
MGSDCRKVVGFLQFFVISFFLCSSPLFCDAADSITKGRGLRDGSNETLVSLDDSYELGFFSP
INSSLRYVGIWYHKIEEQSVIWVANRDRPLRNRNGVLIIGDDGNLVVLDGNNSVWTSNITA
NSFEPRNLTLLNHGALVLSSGDDLSKVHWSSFEHPTDTFLPNMVVKVNPQMGEKRMFMS
WKSETDPAVGNYCLGVDPRGAVQIIVWNGNNRWWRSGHWDKQIFSGIPTMRSTSLYGFKI
TSDDGNNISVTFEALNDLDKLKFQIQWDGKEAQQRLNETTRKWDTIRLLPSNDCDFYNFC
GDFGVCSENSRLKCSCPQGFIPKNKERWDKGIWSDGCRRKTPLLEQRMKSSPNGTIEDSE
QDGFVDVLFVKLPDFITGIFVVESCRDRCSSNSSCVAYSDAPGIGCATWDGPLKDIQRFEG
AGNTLHLRIAHSDLTPVDSESKLSTGVIVAICFGGAAAIAIIALLLWKFRGKTKAATTSEPQ
NKTEVPMFDLSKSKELSAELSGPYELGIEGENLSGPDLPMFNFNCIAAATDNFSEENKLGQ
GGFGPVYKGKLPCGQEIAVKRLSVRSGQGLEEFKNEIILIGKLQHRNLVRLLGYCIQGEDK
LLLYEYMPNKSLDWFLFDPNKQALLDWKKRLSIVEGIARGLLYLHRDSRLLIIHRDLKASN
ILLDEDMNPKISDFGMARIFGGNQNEATNTIRVVGTYYVSLETLE
> gene:Csa6G052130
MNFHTHLFPCFIAFAFAVSFSEAAITLGSSLRASDPNQAWNSSNGDFSLSFTPLGSSSFKAGI
VFTGGVPTIWSAGGGATVDASSALHFQSDGNLRLVSGSGAVVWESHTTGLGVSSAVLEDT
GNLVLLNSSSQPVWSSFDHPTDTIVPSQNFTLGMVLRSGQYSFKLLDVGNITLTWNGDEG
DVIYWNHGLNTSIGGTLNSPSLRLHSIGMLAVFDTRIPAGSFVAYSNDYAENAETTFRFLKL
TSDGNLEIHSVVRGSGSETTGWEAVSDRCQIFGFCGELSICSYNDRSPICNCPSANFEPFDS
NDWKKGCKRKLDLGNCSNGINMLPLENTKLLQYPWNFTGIQQYSMQISGCQSNCRQSA
ACDSSTAPSDGSGFCYYIPSGFIRGYQSPALPSTSFLKVCGDVDLNQLESSDVSRPGDKVK
VWVLAVVVLVTLFAMIAFEAGLWWWCCRHTSNFGGMSSQYTLLEYASGAPVQFSYKEL
HRVTNGFKDKLGAGGFGAVYKGVLTNRTVVAVKQLEGIEQGEKQFRMEVATISSTHHLNL
VRLVGFCSEGRRRLLVYELMKNGSLDGLIFKGEEGQSGKFLSWEDQTNHKRFSLWAYEEF
EKGNLIEIVDKRLVDQEIDMDQVSRVVQVSFWCIQEQPSQRPTMGKVVQMIDGVIDIERPP
APKVTSMVSTSGTTSTYISSNLSNFSTTPTTETPASFSSSHAALDMTPGGSKIEKTSSSLLQS

RYD
> gene:Csa3G733880
MFFSFPFLSSLLLASTAVWAAASAGLQSLTPGNSIAVEDENQFLISPNGTFSSGFYPVGNNS
YCYSIWYTKSFEKTVVWMANRDKPVNGAKSRLTLNIDSNLVLTDADGTIVWSTDTVSNG
EIQLRLLETGNLVVMNQSQNFIWQSFDFPTDTLLPQQRFLKTSTLVSMQNRGVYLSGFYFF
KFNDYNVLNLLYNSPSLSGIYWPDTMVTVFVNGRSPYNSSRIAILDEMGGFESSDKLKFNA
TDYGLGPKRRLTVDFDGVLRLYSLVESTGNWTVTWIPSGARIDPCLVHGLCGDYGICEYD
PLPTCSCPPGFIRNDPSDWTKGCKPLVNLTCNSINPSKEMDFIALPNTDYFGHDWGYVDKF
SIEMCKDWCLSSCECTGFGYALDGTGQCYPKMALRNGYRKPSTAVRMFIKVTKDEYSLSL
ALRHSTNELNCSVSQIVLGTEHVYAEKSNKFRSMGLLVGVVVAIGISELIFVGFGWWNVF
RKRVNEELVNMGYIVLAMGFKRFSYDELKRATKNFKQEIGKGGFGTVYKGELDDGRVVA
VKRLDGVLQGEAEFWAEVSIIGKINHKNLVKLWGFCADKHHKMLVYEYVKNGSLDKFLF
SDSSQVLGLEQRYEIAVGTAKGLSYLHEECLEWVLHCDVKPQNILLDESMEPKVADFGMS
KLFREINESGFSKVRGTRGYLAPEWMMNLKIDAKADVYSYGIVVLELLSGKTAYGFESST
VCKDGGRNIDMVKWVMEVAEKGEVEKVMDPRLKVEDKQNKKKIDILLKVALLCVKEDR
NMRPAMSRVVELLTGYEEPSSHGDVC
> gene:Csa3G733860
MFISALLISLLLSPSLAWPEGTTTLTQGNSIDVEDENQFLTSTNGIFSSGFYKVGNNSFSFSI
WFARSADKTVVWMANRDNPVNGKQSKLRLNFNGNLVLTDADGSFTWSTNTITTQQVEL
KLLDNGNLVLVNQIGVFLWQSFDFPTDTLLPQQQFLKNSTLVSIKTPGTYSSGFYFFKFND
DNVLNIIYNSPSLSSIYWPDPGKNVFDNGRSRYNSSRVAILNDMGRFESTDNLNFNAIDYG
FGPKRRLTMDFDGVLRLYSLVESTGSWEITWLPDGPLDACLVHGLCGEFGICSYTPLPTCIC
PPGFIRNHPSDWSKGCKPSFNLSCDSKDLDFIQLPRTDYYGYDLVGFARGVSVETCRNSCL
NSCQCLGFGYSTDGLGLCFPKGVLRNGNRKPDTMRLMHIKIPKGRPKTELKEEFSNDLKC
SASEIVRNTEIFPENKIKFRYMGLLIAFVAIAGFIELIFFGFGWWNVFRKRVNEELVNMGYIV
LAMGFKRFTYAEMKRATRNFKQVIGKGGFGTVYRGELDDGRIVAVKRLEGILQGDAEFW
AEVSIIGKINHKNLVKLWGFCAEEKHKILVYEFVKNGSLDKLLFSNNSSQPL
GLEQRYEIAVGTAKGLAYLHEECLEWVLHCDVKPQNILLDEELEPKVADFGMSKLFKEID
ENGFSRVRGTRGYLAPEWMMDQKIDAKADVYSYGIVLLELVSGKSASNFQSSSNSMDFR
YSNLVSWMIDNVEKGKMEDAIDPRLEESEKDVRKIEMLRHFLELKIEAEETPESHRISISYY
FFIPFPAFLRRLPHPHRLYSLFYSIIWILAASVIEFVTATVSFFGFFGKAEL
> gene:Csa3G099580
MDSFSATLLAFNLALLLFRSAAISDSLTAQNPYLRDGLSLVSTNGTFELGFFSPGLPSNRYL
GIWYKNRRGPTSVWVANRKTPISGSSGVLVMNITTGNLTLFGHNSTVVVVWSARLLRKVPN
GVLQLLDTGNLVLRDRDDENPQNYSWQSFDYPSDTLLPGMKLGWDLRYNIERRLEAWN
NLNDPSPGDLSWRMELHEYPETVMWKCSRKYVRHGPWNGVRLSSRPLAAAPILNFNFVS
NENEVYYQISVVNKSHSVMLVMNQSTYTRILYLWSAAERRWRVYTSLPRDYCDNYALCG
PYGYCDIRVTPSCKCLEGFKPRSPDTWKTGEFADGCERNKLMNCGDEVGFAHLNQMKLP
DTKHTWVNKSMNLEECRQKCLRNCSCMAYANTNISGSGSGSGCALWIGDLIDLKLIPDAG
QDLYVRMLASELVMHRKAHKTGRLNSKVKIALFAISGFGLAIIFFIGVYIFRRRSTIKDGHE
KIEAHHLELPLFDLSLINSATDNFSLNNKLGEGGFGPVYKKYIYKSLNSRVSLQMDKMLHI
WCLGVYLSFLLDLVNFKLTDKTQRQLLSWSNRYRIICGVARGLMYLHQDSRLRIIHRDLK
ASNVLLDMDMNPKISDFGLAKTCGGDQTGGRTLRVIGTYCLLQFFYSKFYDWKKNSTLH
FLNDRHGDYGKKEICINISLLCVQQHPNDRPTMSSVVMMLGCEIPLSQPKQPGFFIENEGIE

MKRCSSEDKSTSTNELTVTLPDPR
> gene:Csa4G289630
MASHQNAFSFFFLFLFLFLPSFSVSAQPYKNVTLGSTLTALNNNNDSYWSSLSGDFAFGFL
QFESKGFLLAIWFNKIPQQTIVWSAKPSALVPAGSTVQLTNTQLVLKDPAGKQIWSSNDNN
NVGLGSVSYAAILDTGNFILTATDSQVLWQSFDHPTDTILPSQTLNSNLVSSYSKTNYTEGR
FLFSMGTDGNLVSSYPRIVPMRWSPLIYWESETSGSGFNLVFNLSGSIYISAPNGSVVKNLS
SNTPSTDDFYHRAILEYDGVFRQYVYPKTAKITGNATPSPWPKDWSQVSDSIPPNMCLPIT
NGLGSGACGYNSYCRIGDDQRPTCHCPQGYDLLDPNDEIQGCKPIFTPQSCDDEETDAFEF
FSIENSDWPDADYEAFYGVNEDWCRRVCLDDCYCSAVVFRGTHCWKKKFPLSFGRIDLE
FKGKALIKVRKQNSTSIIVNQAYKKVKDKTLVLVGSIFLGTCGFLIATLLIAYQFNIKRTELL
IEKNLPVLQGMNLRIFSYEELHKATSGFTEKLGSGAFATVYKGVIDDCMDKEIKNLVAVKK
LENMVKEGDQEFKAEGSLADYLFGCTKKPNWYERIEVILGTARGLCYLHEEWYVAPEWF
RNLAITTKVDVYSFGIVLLEIISCRKSLEVEGEDELVVLADLAYDWFQERKLEMLVRNDEE
AKEDMKRVEKFVKIAIWCVQEEPSFRPSMKKVVQMLEGAVEVSTPPHPYSFITAIH
> gene:Csa4G290150
MASLCFPSPSLLLLLLLLLTPAFTVAQTTNPNITLGQSLTAHSANSFWSSASGDFAFGFRQSG
GGDYLLAIWFNKIYDKTVVWSANRNKLAPEGSTVLLTTTGQLLLNDPAGNLIWASPTNQS
VSFAALLDNGNFILAANNSEIVWQSFDYPTDTILPSQILNQGDSLVASYSETNYSSGRFEFS
VQPDGNVMLYTRNFPSELISQAYWSTGTVSFGFQVVFNLSGSIVLIAENKTILNTLSSNNPT
AQTFYQRAILDHDGVFRHYIYRKGDTGSTSSWPKAWSLSKSIPSNICLAISQGSDSGACGF
NSYCRLGDDQKPFCSCPEGYALFDPNDVTRSCKPNFVPQSCDKSFPETDDFYFVSMDNTD
WLLGDYGHYLPVNEDWCRNECLNDCFCAAAIFRDGNCWKKKFPLSFGRMDYSVGGKA
LIKVRRGNSTLQSQNLDRNCNNKTKIIIGSVLLGSSLFLNILLFLLTLLISYRFSKRKLLKFN
GGDPFILGVNLRAFSYEELNKATKGFKEQLGSGAFATVYKGTTLGSVDDNNLVAVKKLEN
IVNEGSGENEFKAEVSAIARTNHKNLVKLVGFCNEGEHRMLVYEFMENGSLADFLFKPSR
PTWYRRIQLVLGIARGLSYLHEECRKMEKLIREDEEARSDMKRVERFVKIGIWCIQEDPSL
RPSMKKVIQLLEGDKETWCMWISSCFKT
> gene:Csa4G289640
MAFPSFFLLLLLPLFSLPSFSFSQPYKNITLGSSLIASPRNHTNHSYWSSPSGDFAFGFLDTG
TNGFLLAIWFNKIPENTIVWSANPNHLVPSGSILQLTTHGQLVLNDSAANQIWAANFQTEN
TTVSHAAMLDTGNFILAAANNNSQVVLWQSFDEPTDTILPSQVMKPDTILIARFSKTNYSD
GRFHLRMESDGNLVLYTRIVPLGSQGNPYWSSNTVGSGFNLVFDLSGSIYVSAKNGTALTY
LTSKNPSSNQHNFYHRAIFEYDGVFRQYIYSKSDEAWKSVSDFIPLNICASINNGLGSGVCG
YNSYCVTGEDQRPICKCPQGYYMVDPNDEMQGCRPSFIPQICSLAEANSFDFFSIERSDWT
DSDYEGYSGTNEDWCRRACLDDCFCAAVVFETGNCWKKKFPLSFGRVNPDFRGKALIKI
RRDNSTLIDDNLVKRGKDKTLLIIGLVLLGSSGFLIFISLLAVLIVYRIKKKRSEGVMGKVAA
SIGVNVRAFSYEELNKATNGFTEKLGSGAFATVYKGILDDDDCLDKDNKLVAVKKLEIEV
KEGEQEFKAEVSAIARTNHKNLVRLLGFCNEHLHRLIVYEFMPNGCLADFLFGPSQLNWY
ERIQLARETARGLCYLHEECKTQIIHCDIKPQNILLDESLRARISDFGLAKLLKENQTRTTTA
IRGTKGYVAPEWFRSNLPITVKVDVYSFGIVLLEIISCRRSFELEVEDENEMVLADWAYDC
FKERRVDMLVRKDDDEAKGDMKTVEKLVMIAIWCIQEEPSLRPSMKKVLQMLEGVVEVS
IPPDPSSFISTIQ
> gene:Csa4G005510
MGFFKIATFLFVFSLLFIQTNTAIVKSQSIDQINPGFRASASEFNHTNGVFLLSKRSVFALGF

YAGAKDNTFSLGIIHIFSSRVIWTANRDSLVNDSAFFVFNETGDAYLDVSGQNQTTVWSTE
TANEGVVSMQLLDSGNLVLKSKNGSFIWQSFHFPTDTLLPGQIFWEGLKLKSYPNDNDHS
NFLEFKQGDLVLSAGYQNPQIYWALSNDSRKIQRATTGGSGYSLFAILESNYWNFYGTNG
ELLWSFKIFWQLNRKDRWISVLNTDGTISFLNLENRKSAEPEPIRIPAEICGVPEPCNPLFIC
YFDNHCQCPSTVFEKNFNCKLPSVPCNGSSNSTELLYLGENLDYFALRFSTPAFNSDLSSC
KTACSSNCSCNVMFYEPVSRNCYFFNEIGSFRRSEGGSGGYISYMKTNLPINGNNSETNPS
PNRRKHIVLMSLLMAAMTLGFMGLLCFLFYRQKMKELLSSIDEATEEDIFLNEISGGPIRYS
YRQLRRATKNFSTKIGDGGFGSVYLGKMGDGSRLAVKKLERIGQGGREFRAEVSLIGGIH
HVNLVKLKGFCSESLHRLLVYEYMSNGSLDKWIFNKKEDDLFLDWSTRFNIALGTGRALA
YLHQECESKIIHCDIKPENILLDENFTPKLSDFGMAKLMDKQHTSIFTQLRGTRGYVAPEWI
TTLAISDKSDVYSYGMLLLEIIAGRKSYDADYPPEMAHLPSYATRMVGEQKGFRVLDSRV
AGEAEGDWRVEAAVQVAVWCVQEEPSLRPPMRKVVQMLEGVSPVPMPPCTAEMGANF
WWSSDGLGMKLNGCYSEVRLSDVRLSGPR
> gene:Csa4G289650
MASHCFPFPSLLLLLLFLLTPSFTVAQATSPNITLRKSLTARSSDSFWSSASGAFAFGFRQAV
GGDYLLAIWFNKIDEKTVVWSANRDKLAPGGSTVVLTTSGQLILNNPAGKQIWSSTSTAP
NKSVSSAVLLDNGNFILAANDSEIVWQSFDDPTDTILPSQILKKGNKLVASYSETNYSSGRF
EFYMQTDGNLLLYIRNFPYDAISNYYWSTDTVNFGFQVVFNLSGSIVLIAENKTILNTLSSN
NPTAQTFYQRAILDHDGVFRHYIYPRGGTGRNSSWPKAWSISKSIPSNICMTIGQSSDGGV
CGFNSYCKLGDDQKPFCSCPEGYVLFDPNDVTQSCKPNFVPQSCAFPELDDFDFVSLDNS
DWPQSDYGDYGHNIPVNEDWCRNECLNDCFCVAATFRDGNCWKKKFPLSFGRMDYSVG
GKALIKVRRRNSTLQSRNLDKNCNNETKIIIGSILLGSLFLNILLLLLTLLIGCRFSKRKLKFN
GGDPFILEQLGSGAFATVYKGTTLGSVDDNNLVAVKKLENIVNEGSGENEFKAEVSAIART
NHKNLVKLVGFCNEGEHRMLVYEFMENGSLANFVFKPSKPTWYTRIQLVLGIARGFGIML
LEMICCRKNFEMETEDEDERILSDWAYDCMNEGKMEKLIREDEEGRSDMKRVERFVKIGI
WCIQEDPSLRPSMKKVIQMLEGVVEVSTPPDPSSFISAIN
>gene:Csa7G446780
MFLFPQLLILFCCFFVALFMAKFSHGHTTLANDVLAQGQHLSIAFYIPPSSNSIYLGISDNTN
DQKPIWIANRNSPFPNNSASISLTIDVNGSLKIQSGNCSFSLFNGGQPTTSSAILQDNGNFVL
RELNRDGSVKQIVWQSFDHPTDTLLPGMKIGINHKTNSTWSLISWRNYKSPKPGGLSLGM
NPNNTYELVVCVRGELLWRTGNWKEGSFEFLEKDKGFNFVRVSNENETYFIYYAREPNGY
SLYRNSYYHGESGELILSQIRLENNGNVRINNEIYDSPCLLTSNEIRGACVWRELDKIPECR
NKLSHGYGPYISQINGYELERINGSDYYYKLSGNLTMFECRSICINDCDCIAFGIPAYESDSG
CEFWKSGANFIPENDSLQMLWSLDTDSEFLDTDHEFSNTNDESPNGKWKVWVQITVALTL
PATFLLLCFIIYTKWRTQIFKAIGKVKKGFLRGMGMISECYNILRIMIIQIRDGKKNPELQFF
DFETILSATNNFGEEYSEKKLIFDWEKRLHVVQGIVQGLLYLHYYSRVRIIHRDLKVSNILL
DDEMNAKISDFGMARVFKPSDNEANTSRVVGTHQKNYHNYDTERPLNLIGYVIKNDNVC
IFQAWELWVNGRGEELIDLGLCNSDDQKAKALRCIHVSLLCVQQIPGNRPTMLDIYFMIN
NDSAQLPSPKQPAFFIAQSPSSSQREIEEVDSETHTTHRSNLFIEFYDTLNDGCKSSMADILH
QGQELTTGSQLIPATVIFVLGFNYYPPNTNTMTLPIDTTSAMLQDDGDFVWRELKPRWISK
ANSVAEL
> gene:Csa1G071150
MVLFPRKSFAIDSIKAGESINGSNQILVSAQQKFVLGIFNPKDSIFHYLGIWYMNIPQTVVW
VTNRDNLLLNSSVILAFKGGNLVLQNEREGIIWSSISSEFVKVPVAQLLDNGNLVIRESGSE

NYVWQSFDYPSDTLLPGMKLGWDSKTGMKWKLTSWKSLNDPSSGDFTFGMDPDGLPQF
ETRRGNITTYRDGPWFGSRFSRSSFFSEVEITSPQFDYNAEGAFFSYESVNNLTVIYALNAQ
GYFQELYWKDDANDWFSLNELPGDGCDDYGHCGNFGICTFSFIPLCDCVHGHRPKSPDD
WGKHNWSGGCVIRDNRTCKNGEGFKRISNVKLPDSSWDLVNVNPSIHDCEAACLSNCSC
LAYGIMELPTGGNGCITWFKKLVDIRIFPDYGQDIYVRLAASELVVIADPSESESPKRKLIV
GLSVSVASLISFLIFFACFIYWRRRAEGNEVEAQEGDVESPLYDFTKIETATNYFSFSNKIGE
GGFGPVYKGMLPCGQEIAVKRLAEDDKKRSLLGWKKRLDIIIGIARGLLYLHRDSRLIIIHR
DLKVSNILLDNEMNPKITDFGMARMFGEDQAMTQTERVVGT

> gene:Csa4G289620

MTSQQKPTWKVKRNLYTNCYLSINAIHLHKSLTFLQLMAFKITPSYFLFPPFLHSLLLLLLV
LPTCSFSQLFKNVTLGSSLTATQLNDHHNYWVSQSGDFAFGFLPLGSQGFLLAIWFYKIDE
KTVVWSANRDKLVSKGSTVQFTSAGQLVLNDPGGNQIWTATASSSGNTNRSVSYAAMLD
SGNFVLAATDSEILWQRFDVPTDTILPSQTLNMGGALVARYSETNCKSGRFQLLMQTDGD
LVLFPHPLEKTNISYWASNTTRSGFQLVFSLAGSIYVIAKNNTILTTVVPNTLSPQNYYLRAI
LEHDAVFRLYVYPKVTSNSTMPKAWTQVSDPVNICIMVSNGTGSGVCGFNSYCKVAQRC
DKSFIETDDFEFVAMENTNWPHGDYANFNPVSEDWCKNECLNDCFCALATFRNESKEQN
NNHCISSSRKLYFPKLSLNFILFLLTLFICYHFRKRKSDVVEKDPSILGVLNIRIFSYEELNNA
TGGFIQHLGRGSFATVYKGIIDSDNNNNLVAIKKFDNVVPDGEQEFKAEVIAIARTNHKNL
VRLLGFCNEGEHRMMVYEFMPNGSLADFLFGTSKPNWHSRIRIILETARGLCYLHEGCST
QTIHCDIKPQNILLDESFSARIADLGLAKLLKKDGARTTPMTMTMTNGESKGYVAPEWFR
GLPITVKVDVYSFGVVLLETICCRRSLEEKEENEKQKVLRDWGYECLKEMKVEMLVEKD
EEAKMELKKVKKFVMIAIWCIQEEPSLRPSMKKVLQMMEGGIEVSFPPHPSSFISSIS

>gene:Csa7G045520

MEEDRNSGEGTCLLHNRSSEMVAKRSSVKKLVTISWFAEHLMSFFHLYSFVFLIFVVNCFA
KDTLEFKSCISHGSGDTLVSAGSRFELGFFQPYGSSHSRRYLGIWYYKSNPITVVWVANRD
RPLPSSDGVLKIEDDGNLKVYDGNQNLYWSTNIGSSVPDQRTLKLMDNGNLVLSYVDQE
DLSEHILWQSFDYPTDTFLPGMLMDDNLVLASWKSYDDPAQGNFTFQLDQDGGQYVIWK
RSVKFWKSGVSGKFITTDKMPAALLYLLSNFSSKTVPNFSVPHLTSSLYIDTRLVLNSSGQL
HYLNWEDHKVWSQIWVEPRDRCSVYNACGDFASCNSECGMACKCLPGFEPTSPGSWNI
GDYSGGCIRKSPICSVDADSDTFLSLKMMKAGNPDFQFNAKDDFDCKLECLNNCQCQAY
SYLEANITRQSGNYNSACWIWSGDLNNLQDEFDDGRDLNVRVAVRDLESTARNCGTCGT
NLIPYPLSTGPKCGDPMYFNFNCNLASGQVNFEAAGGTYKVKFIDSEARKFYIQTKEPGD
CGDKNWITKALQLNQSSPFRVTSWCNFKETNLEENFSLKTSNEVEISWEPPLEPICSSTTDC
KDWPYSTCNMSKDGNKRCLCITDFHWNGWILNCTTDHNKGKDGKGKTTFSVIIVATSLC
MVLLMILSCTVFYIYFSKSGLIERQESRGNSQKDLMLHLYDNERRVKDLIESGRFKEDDTN
GIDIPFFDLETILVATDNFSNANKLGQGGFGPVYKMSVALDWDMRFNVILGIARGLLYLHQ
DSRLRIIHRDLKTSNILLDEEMNPKISDFGLARIFGGKETATNTKRVVGT

> gene:Csa4G288620

MALKITSSYFLFPPFLHSLLLLFILLVLPTCSFSQLFKNVTLGSSLTATQLNDHHNYWVSQS
GDFAFGFLPLGTNTFLLAIWFDRIDEKTVLWSANRDNLVPKGSTFQFTNGGQLVLNDPGG
NQIWTATVSSSGNSNRSVSYAAMLDSGNFVLAAADSEILWQSFDVPTDTILPSQTLNMGG
TLVARYSESTYKSGRFQLVMQTDGNLVIYPRAFPLDKASNAYWASNTMGSGFQLVFNLSG
SVDVIANNNTVLSTVLSTTLSPRNFYLRAILEHNGIFGLYAYPKPTHSSSMPRAWSQVSDSI
NICILVQTGWGSGVCGFNSYCRLGDDQRPFCSCPPGYILLDPNDEIKGCIPNFVAQSCDQSF

HETDNFEFVAMENTNWPSANYGYFKVVSEEWCRNECLNDCFCAVAFFRNGECWKKRFP
LGDGRMDPSVGGRALLKVRKQNSSFQPNDLVHKPTIVVVGSVLLGSSVFLNFFLFLLTLFI
GYRLKKRKSKPVQRDPSILDVNLRIFSYEELNKATSGFIHQLGRGSFATVYKGTIDSEDNN
NLVAVKKLDNLVQEGDQEFKAEESTECWCMNSCIMGLLQISFLGLRNQIDQTRTLTAIRGT
KGYVAPEWFRSLPITVKVDVYSFGIIMLEIICCRRSYEKKVEDEEQMVLTDWAYDCFKDM
KVEMLVENDEEAKMDLKRVKKFVMIAIWCIQEEPSLRPTMKKVLQMLEGAIEVSFPPDPC
SFTSSSTII

L-Type

> gene:Csa5G648630
MAPLTFLILFFTVPPYFFADSKFLYNGFHEGKGLNLDGAAIVKPSGALCLTSNSQNVVGHA
FYPDPVMLFDPRSPSNTSSFSTTFVFAIDPSIPGHGGHGLAFTLAPSTRFDEAESGHYLGLFN
PLNDGNPSNHIFAVEFDTVKGHGGVTNSRGNHIGININGISSVKSQLAASSYYVDDTVWKE
IQIDSGDPIVAWIDYDGRSKNLSVTIGLLELKPEKPLILCHIDLTSVMKNQMFVGFAASTGIE
TSAHYILGWSFAVDATARQLKYSQLPNRPNEQNISSSSNNNSQLKSVLAVSSIIVLMAIVILT
FLFIRMKKAESLEDWEKDCPHRFNFKDIYTATNGFNDSAQIGIGGFGSVYKGKLSSTGAEI
AVKRVKRDSSQGMKEFAAEIESLGRLRHKNLVNLQGWCKKQNDLLIVYDYIPNGSLHSLL
HTSKQSVILKWEQRFNILKGIAAGLLYLHEDWEQVVIHRDVKPSNVLIDADMNARLSDFG
LSRQYDHNEMSHTTRVVGTIGYIPPELFRTGKASKSADVFAYGVLLLEVACGRKPLGSNQF
ILMDWVMEWYETGDILHVADPKLDSIYKVEEMEMVLQLGLLCTHWKQEARPSMRQVM
RFLNREDPLPASDAWTNSQSIFESSSRLTMTDRSSSMSVGPISSASINEGR
> gene:Csa7G067430
MSHMEVSILTSPSCLLLLILCYSNIFWSLTNPGSVSAIISYENLTFNLTDFGPNDHDIHYEGDT
YPSNNVIQLTMNQRDMPLNGSVGRATYRDPFHLWESGHRNLADFTTQFTFTIDSQHSRTY
GDGFAFFIAPVESRLPPHSGGGNFGLLSSNKSDPDVVPTANFVAVEFDTYTNAWDQSENHV
GVDVDNVKSLSSTSWWWSDIENGGKVKAAISYNSSYHNLTVFLVDERDSEVSPTNSSTFT
FNIDLREHLPEWVTIGFSGSTGSFFEIHTISSWSFSSILQVEVNVTTTTEPASSPVNSKKGINM
KWFGIIFTVALSLFLILGFVWFGVWMKRTSRRKSMRRNQEEDFENETGPRKISYKDLLAAT
NKFSDENVLGQGGFGKVYRGFLDNKELDVAVKRITPNNLHQGSREFASEVKTISKLRHKN
LVELIGWCCCSKDQEYLIVYKFMPNKSLDFHLFQQNNLLTWDHRYKIAIGLALALHYLQE
EQDPYILHRDIKSSNILLDAEFNAKLGDFGLAKLVDHGKQSITTILRGTEGYVAPEYLESSV
ASKESDIYSFGIVCLEIACGKQALGEAREDGKRRLIKLVEWVWDYYRRSVEEAADPKLRQ
NFKREEMKQLLIVGLACAQPDFRVRPSIKQVIDMLNFKSPLPNLPLEYPGLSRSAVFLSAE
MESLRTSSCLQSGNLHGKSISSKNSTASSTFSNMG
> gene:Csa3G730920
MAIAAHLALLSVFIFFEAHALPSSFIYPGFNNTSLDREGASVVKPYGALRLTNISQNVIGHA
FHPTSFRMFEQSSDSSPNVLSFSTTFVFAIEPSSPGQGGYGLAFAIAPSTKFSGAGSGHYLGL
FNSSNNGNPSNHIFAIEFDTVNGHGEERNTKGNHVGIDINDISSVTSKPASYSDYGEAHEH
DLQMDSGDPIIVWVEYDGPKKIVNVTIAPLKHKRKPTKSLLSYPIDLKPFLKEQMFVGFSA
STGDKTSSHYILGWSFAMNEPAPPLDYSLLPNPPKEQDPPSSSPNSRYKVFVAVVSVIAILGI
FFLAFWYRKTWHTERLEDWERDCPHRFHYTDLYTATKGFKSSELIGIGGFGSVYKGQIRST
GIEIAVKRVRRNSGQGMKEFAAEIESLGRLRHKNLVNLQGWCKKKNDLLIVYDYIPNGSL
YSLLYHPKNNIILNWKQRFNILKGIAAGLLYLHEEWEQVVIHRDVKPSNVLIDADMNPRLS
DFGLARQYDHDEASHTTGVVGTIGYIAPELVRTGKASKSTDVFGYGVLLLEVACGRKPLK
SDNFILVDWVMEQYEKGKILEAADPKLNWEYEAEEMKMVLVLGLHCSHQIAEARPTMR
RVMRILDGDDKIAAVEGWDCSQSYSKSNSRMTEVISATSYRSSSIGDISETSIDAGR
> gene:Csa4G296250
MASSSFLFIIFFLSLIFLSTHTLSSHYTQLFYPGFYGSIDITLKESAEIESNGVLRLTSRNSRNN
IGQAFYSSPIQFKNSSSDGGRGPSFSTCFVFCIIPENEGGHGFTFAIVPSKDLKGISQRFLGLF
NESNFYGILSNHIFAVEFDTIFDVGIKDIDNDHVGIDLNSLISNATVHAAYFDELGKVHNLSL
QSGKPIKVWIDYDSDEITLNVTISPFNSKPRNPILSYRVDLSSIFYEEMYIGFTASTGLLSRSS

QFILGWSFAINGQARDLDISSLPLPKKKKTREKISLPVYVSITTASVFVISVFVLGFYLLRKC
KKSEEIEPWELQLGPHRYSYRELKKATRNFSEKELLGYGGSGKVYRAILPISKTQIAVKRIC
HDSKQGLREFMTEIATIGMLRHRNLVQLLGWCRRERDLLLVYEFMENGSLDNYLFDDPV
RILDWEERFKVIKGVASALLYLHEGYKQVVIHRDVKASNVLLDGELNGKLGDFGLAKVY
EHGSAPDTTRVVGTLGYLAPELPRTGKSTTSSDVYAFGALMLEVACGRRPVEVKALPEEM
TLVDWIWDKYREGQVLSVVDSKLQGVYDEVELTMVLKLGVMCSNNVPEQRPSMRQVV
RCLDGEIGVVDEWKSPGGGSKGGGVGDFLGSFTSTSISGESSG
> gene:Csa4G296230
MAAIVFLTLILLISISPASADQRFIFNGFNFGDLFLDGVAEVTSDRLLRLTNDYDQQIGHAFY
PNPISFKTPSNNNSSLSSFSASFVFAIISEYDDLGGHGIAFVVSPTRGLPGARPSENLGLFNES
NNGKETNHIFAVELDTIQNLDLRDINRNHVGININGLMSEQSERAGYWVDGEFRNLTLISG
ERMQVWIEYDGLKKQINVTLAPIEIRDKPKIPLLSYRRDLSSVINDIMYIGFSSSTGSITTLH
YVLAWSFNVNGEAQKINLSQLPKLPPRTKKKPSRSKLLTIGLPLVCVALALMTVLGLIYFIY
RRRKFAEILEDWELEYGPQRFKYKDLYKATKGFREKEFLGFGGFGRVYKGVLPNSKIEVA
VKRISHESRQGMKEFVAEIVSVGRLRHRNLVALLGYCRRKGELFLVYDYMRNGSLDAYLH
EWPEITINWEQRFEIIKGVASGLFYLHEQCEKVVIHRDVKASNVLLDDEFNGRLGDFGLAK
MYDRGADPRTTHVVGTLGYLAPEHIRTGRATTRTDVYAFGAFLLEVACGRRPIHPQEDSD
DFILMDWVFSCWSNGNILRTADPKLGGNFEPSQLELVLKLGLLCSHSSPAVRPTMYQVLQ
YLQAEAPLPDLASLRWRLSGNGFFNLTPRDGDELDDFTGSVRQSSLLLDSIFR
> gene:Csa3G736960
MMLNFSSIFSSITFTLLLFSISNTVIVDFTATVAAAAEFDFGTVELSSLKLLGDAHLNNGSVR
LTRDLAVPNSGSGRVLYAKPIRFRQPGIDYLASFSTFFSFSITNLNPSSIGGGLAFVISPDAET
LGGAGGLLGLADERGLGFVAVEFDTLMDVEFKDINGNHVGLDLNEMVSLEVEDLQGIGV
DLKSGDTVNAWIKYDGSARIFEVFVSYSNLKPTEPLMSFNLDLDPYLNDFMYVGFSGSTQ
GSTEVHSVDWWSFASSFDSDSTPGSVPPPPTTTLMNPTANVVRSPPPSQPPSGSDSVTQKN
FKSTSCHNGLCKQGAGAVVGVVTAGAFVLALFAGALIWVYSKKIKRVKKSDSLASEIIKM
PKEFTYKELKIATKCFNSNRIIGHGAFGTVYKGILPETGDIVAVKRCSHSTQGKNEFLSELSII
GTLRHRNLVRLQGWCHEKGEILLVYDLMPNGSLDKALFEARTPLPWPHRRKILLGVASAL
AYLHQECENQVIHRDVKTSNIMLDEGFNARLGDFGLARQVEHDKSPDATVAAGTMGYLA
PEYLLTGRATEKTDVFSFGAVVLEVASGRRPIEKDSTAVGGGGKFGANSNLVDWVWSLHR
EGRLLTAADGRLGGEFEESEMRKVLLVGLACSHPDPMTRPTMRGVVQMLIGDSEIPIVPRS
KPSTSFSTAHLLLTLQDSVSDLNGMIAISTSSSEHSFNGEDLISLDDRTVSNPSIV
> gene:Csa7G067400
MAAIISISSYLLPLFFFFIYILSFFSTLTLANSLAFNFSSFDSSNTHIFYEKAFPSNRTIKLTGET
VNKNQNFTGRATYFKPFHLWDKPSGNLSSFQTHFSFAIDSEGAERYGDGLTFFFAPNNSRL
DAEISKGSGLGIGYNPSLTNLTYSSFFAIEFDIFSNFFDPPQKVEHVGIDINSMSSVAYSIWKC
DIKSGRRTDVWINYDSATLNLSITFTGYENNKTILQRLNHDVDFRLTLPEWVTFGFSAATG
TLYATHNIYSWDFKSTLNLNSDSNLAPSPGQGSKVGLVVGVGVGVGVVFLVCGLIIVWICF
LKKRKKRMKMNWEEDVVLDDSEFEKGKGPRKFSYSELARATNNFWEDEKLGEGGFGG
VYKGFLRDLNSYVAVKKVSKGSRQGIKEYASEVKIISQLRHRNLVQLIGWCHERGELLLVY
EFMSNGSLDAHLFKENNFLTWEHRYKVAQGIASALLYLHEEWEKCVIHRDIKSSNVMLDS
DFNAKLGDFGLARLVDHAIGSQTTVLAGTMGYMAPECAISGRASKESDVFSFGIVALEIAC
GRRPYNPNVEEAKMVMVEWVWELYGNGRLLEAADTKLHGSFENEPQQRQQIECLMVV
GLWCAHPDINCRPSIRQAIHVMNFEASLPVLPLQFPTLAYHHHPLSVNRPIISSSFSSTQDSV

VSQSTGNGFNSTNVLTTSQETTTSSTSFSASTSLLNTR
> gene:Csa7G029930
MNLMRKMFMCKRELRTFVVVVTSLVLLFGPVSSQPILEHRFSYVGFNERENNQAFTFTPS
SSIDGGALQLTPDSQNDVVKLQNTSGRIMYHEPFKLWLNDSDKKEKSDTVASFSTYFYINI
FRREEWTAGEGLTFLIAPTSVVPEQSWGQWMGLTNETIDGDEKNQIVAIEFDTQKQDFDPD
NNHIGLNINSVKSRKTVPLKEAGIVLSPEVGTNHSIWVEYDGKAKLLQVYMSINKDPKPN
KPLLNETLNLKEFVKQESFIGFSASTGSPEIQLNCVLEWTLEMERLPEKKNLTWLKILAGV
GIPVLTIAILVGVWLFVGYRKKRREHVDEESNVQGTLKRLPGMPREFKYKELKRATHNFH
ESMVLGNGGFGIVYKGVLQDKDRDITASSNSGSRLEIAVKQFSRDSIKSKSDFLDELTIIHR
LRHRNLVRLEGWCYEKGKLLLVYDFMPNGSLENHLYDVDEQNVLNWGHRYKILCGVAS
ALHYLHNEYDQKVLHRDIKSSNILLDSEFNARLGDFGLARALDPERNSYADLHCGGVAGT
MGYVAPECFHEGRATPESDVYGYGAVVLEIVCGRRPGAVVEDEQDHYSLIDWVWKLHRE
GHIEKAVDNQLGNDIVVDEARRLLLLGLACSHPVASERPQTQAILQILNGAVPPPHVPPFKP
VFMWPPMSSSSTSSILTSLSNTNNSLS
> gene:Csa3G734050
MASNFDRPLRRNGAYDARLHGREMNKNPMFFNLSIVVSLLLVSVVSSAASKGTDFIFHGF
QSANLSLDAMAVVTSNGLLKLTNETRQKIGHGFYPNPVNFVDFSHGVSSFSTTFVFAIISEY
PNLSGHGIAFVVAPTKTFPGAEPSQHLGIFNDNNNGNTNNHIFAVELDTIQNLELKDIDANH
VGIDINGLISKKAETAGFYPSNNGEFRNLSLISGQPMQVWIEYDGINKEINVTLAPINIPKPK
IPLLSYVWDLSSVIKNSSMFVGFSSSTGSVSTSHYILGWSFRLNGQAQSLDISRLPKLPQKK
HRSKVLTIGLPLISGGIALLVILSIVHVIRRKRKFAELLEDWELDYGPHRFKYKDLYTATNGF
KEKEILGSGGFGRVYKGALPKSKLEIAVKRVSHESRQGMKEFVAEIVSLGRLRHRNLVQLL
GYCRRKGELLLVYDYMQNGSLDKYLFNETNPSLNWSQRFRIIKGVASGLLYLHEEWEQT
VIHRDVKASNVLLDNELNGRLGDFGLARLYDHGTDPQTTHIVGTLGYLAPEHTRSGRPTT
QMDVFAFGAFLLEVATGKRPIEIRGMMEDVILLDWVLLCWMRGAIVEAKDPKLGTEYVT
EEMEMVLKLGLLCSQSNPMARPSMRQIVQYLEGDAVMPEMGSIETLGGGGGYEGFDDLG
MSYNSFLDKAIAYSTSSLERGFAHSSDAQSLLSGGR
> gene:Csa2G439150
MLTHIFLLFLVFPYLPLLVDSIYFKIDQIKPNENRLLYQGDAVPNNGGIIFSDPAYSCLVGQAI
YKDAIPIWDSQTEKLTDFTTQFSFTIDTQNALHYGNGVAFFLAPAGFHIPPNSAGGYLGLFN
KTYTESSINQIVHVEFDSYPNEWDPNFEHVGININSVSSSNFTKWNVSLHSLDTVDVFISYD
STTKYLSVSWNYEKTPISLENTTLSYMVDLMKILPQWATVGFSAATGAYLERHLLFSWEF
NSSLEMKETVGVGTEKNGKKVDVIVGVTVSVGASILMAIVAFVVRRRLKQKKRKSEKKV
AEEINLTSINDDLERGAGPRRFSHKLLAMATNNFSNERKLGQGGFGAVYRGYIPDIDLAVA
VKKISRGSRQGRKEYITEVKIISRLRHRNLVQLVGWCHDKGEFLLVYEFMPNGSLDSHLFG
KRAHLAWAVRYKVALGLASALLYLHEEGEQCVVHRDIKSSNVMLDSNFNVKLGDFGLAR
LMDHELGAQTTGLVGTLGYLAPEYISTGRASKESDVFSFGVVALEIATGRMSRTSMEAES
HKGLVEWVWNLYGSAQLIDGMDEKMQSDFDKKQVECLMLVGLWSAYPDPNLRPSIKQV
IQVLNFETTMPNLPNKMPVPIYSAPPTSMSSNEASITVSLDMGR
> gene:Csa3G730910
MFDIKSEPVSDVSSFSTSFVFAIVPSSSGPPVGYGLAFVMAPSTQFPDAASEHYLGLFNPSN
NGDPSNHIFAVEFDTVNGHDDETNFKGNHVGINKNGVRSSASESAEYSDYGSDVKTEVYL
DTGDLIQAWIDYDGRSKVVNVTIAPASVIRPTEPLISYPINLTSVLNERMFVGFSASTGKETS
FHYISGWSFAINESAPQLDVSQLPPLKVQSPPPSSPSTFNPLVTVVVPILSAMTLMLILFLA

SIFRRRLRGENLEEWERDCPHRFSYRDLYKATKGFKDSELIGSGGFGSVYRGVLPSTGCEIA
VKKITRNASQGMREFAAEIESLGRLRHKNLVNLQGWCKKQNDLLLVYDYVPNGSLDSLL
YHPKDNLVLNWEQRINILKGVAGGLLYLHEEWEQVVIHRDVKPSNILIDISMNARLSDFGL
ARLYDHDQISHTTSVVGTIGYISPELARTGKASKTTDVFAYGVLILEMACGRRPLESDIFILV
DWVMECYEKGRVLDAADPKLNGIYDMVEMEMVLKLGLLCSHYNPESRPSMRQVTRFLN
GEDQILAFDPSPYSQITFQSSSGFTQFIPPSSRTSTASFLSSTSIDVGR
> gene:Csa1G073890
MAPYRDVLPGHGLVFICVPTTGIEGTSAAQNLGFLNFTLNGNPNNHVFGVEFDVFENEEFS
DPDDNHVGINLNSLTSFITEEAGFWSDGGPNAAGTLNRLRLNSGENYQVWIDYSDFRMN
VTMAPAGMKKPRRPLINTSLNLSGIFMDEMYMGFTSSTGQLVQGHNILAWSFSNTNFSLS
ESLITTGLPSFLLPKDPITKSKWFIAGVTAGGFLVVCFFATILGILIADYRRKARLRAEMEDW
ELEYWPHRLTYQEIESATKGFAEENVIGIGGNGKVHKGILAGGSSEVAVKRISHDNDGMRE
FLAEISSIGRLKHRNLVSLKGWCKKEKGNFLLVYDFMENGSLDKWVFDCDERNMLSWED
RIRVLKNVATGVLYLHEGWESKVLHRDIKASNVLLDKDMNGRLGDFGLARVHGHGQVAS
TTRIVGTMGYIAPELIRTGRASTQSDVFGFGVLILEVMCGRRPIEEGKPPLVDWVRQLAIEG
KITAALDSRLRKKGEWNEEEVGRVLHLGLVCAHVDPNNRPTMRQIVKVLEGKIELDESES
ESMNAYLLQRLKSEGMLCDSGVGFGKNLHPTFEDFLQSYSTSLSWTNSSVTGR
> gene:Csa3G115060
MPIFLGTHRNHHFQSQPLQFFLFSLILFKVQSLSFTFPNFQQNNPNLFFEGDSFTSNGLIQLT
KNQADGPLTDSSGRASYAQPVRLWDAATGQVTNFTTHFSFRVTQLNQSSFGDGIAFFIVPY
ESKLPANSTGGFLGLFSSDLAFDSSKNQVFGVEFDSKQDDWDTSDDHLGINVNSIKSINHL
DWKSSMKDSRTANAWITYNSATNNLSVFLTYDSDPIFTGTFTISTFVDLKSFLPERVRVGFS
AATGKWFQIHNIISWSFNSTLDDNLGGGDKNKNTGLAIGLGVGLGVGICGLILLGLFWWG
KKLRRMEDDVDDSIDDEFEKGTGPKRFTYRELTRATKNFDEAGKLGEGGFGGVYKGLLT
ESNTEIAVKRVSRGSRQGKKEYISEVKIISRLRHRNLVQLFGWCHEKGEFLLVYEFMPNGS
LDTHLFRGQTMLSWPVRYKIAIGLASSLLYLHEEWEQCVVHRDIKSSNVMLDSNFNAKLG
DFGLARFVDHELGSQTTVLAGTMGYLAPECVTDGKASKESDVYSFGVVALEIACGRRPV
ESRAEPDQVRLVEWVWESYGRGEVLKTADKRLEMEFDEQQMEALMVVGLWCCHPDFK
LRPSIRQVINALNFEASLPTLPAKLPVPMYFAPSMNLCNFSYTSSGTPVDRSQCSCS
NCSTYTTQSSGSGSSVSLLKSQRHE
> gene:Csa6G338050
MSMNLSLPIIILIFFLFLSHFPFLSLSFLLNQNITLSGDAHLRNNAIFLTQERDCLSLSPNSSSS
ASGFGSAVYYVNPVRFLDSSTNSSASFSSRFSLSILPTPLCSFPDGFAFLIASDPESFTLSNSHIP
LPNPSHSSPFSFIAVEFDTNFDSNLGDINDNHLGLNVNSPTSLTSVDFRSHGIVLKNGRKITA
WIEYRDDSKTIRVWVGYSQTRPVNPLLAAPMDLSKQFKEFMYVGFSASNGQGSALFIVDR
WQFRTFGLLPSLSPVDTINEGAGCFMCSSEDLNSDNSRFVDASERRKKSGEMSLVFGGLA
AFACSGALILGFVSYTLIKKLRSRVCRGREIDRTCLVKMNRIPTRLSLGEIKLATMGFNQNR
VVGEGGSATVYKGSLPSGVEVAVKRFEQGMANNRLPNPFATEFATMVGCLRHKNLVQLH
GWCCEANELVLVYEYLANGSLAKLLHETSPNSQFVIPWKKRVSIVLGVASALTYLHEECE
RQIIHRDVKTCNILLDADLNAKLGDFGLAEVYEHSSLTRIATIPAGTMGYLAPEYLYYGVP
TVKTDVYSFGVVILEVASGKRPVDEGGMVLVDWIWVLWGVRSLIEAADPRLMGNYDVV
EMERMLMVGLFCVHPNNEKRPTVKEAVRILRGEAPLPVLPLRKPMVGIRPILSDDFEDLE
NPCSDYIAFEEPAWMTPKSEFG
> gene:Csa3G115090

MAMTSYLFLFFILFLFLSPEIHSISFQINNFNSNSNIVYDGDAKPINESITFNGDLGWAIYTQD
LLLCDHTNFKTHFSFLMKNNNNNNSNGGLAFFLAPFEFSPPFNSSPPFLGLYNSTQLIQPSQ
SQILHVEFDTFPNPEWDPPFKHIGINKNSISSSIYSPWNSTNQKTLVWISYNSTAKNLSVSFN
NNIYTTLSLQIDLMEILPEKVTIGFSAALVEDLSIEYWEFSSNLDGNYENDSEKSTDMNLLA
VLIAWVGVFVIAIVSIIIISFIRKKKKDDYEEHGVMKLASIYSDLNKEEALKPRRFSYTYLA
MATDNFAKKRKLGEGGFGEVFEAHLPGANKTVAVKKIFKSSRQVKREYVSEVKIINGMK
HKNLVQLIGWCHEGDDSEFLLVYEFMPNGTLHSHLFGDLPPLSWPIRYKISLGLASALLYF
HEERENSVVHRDIKSSNVLLDSSFTAKLSDFGLARLAKHELNSKRPKLVGTFGYMAPEYIS
SGRASKESDIFSYGVVLLEIVSGKKCCDHSGKGLIELVWDAYGRGELVKAILDKKLGVEF
VEAREVERLSMVGLWCVHPDSTQRPSIKQVIQVLSFQEAMPNLPLEMPLPTFNHASRIYKL
NVAYPEENWSMTCSLKPLRT
> gene:Csa3G048440
MTPKIILPLTLFLLIFTTPRTECIGFDFTSFNIRNLTLLGDSHLRDGVIGLTKELGVPSSSAGTV
IYNKPIGFYDADANFTASFSTRFSFSITNINPTSSGDGLSFFLSPDNQTLGSPGGYLGLVNSS
QLTKNKFLAVEFDTRLDSVFKDPNAHHVGFDIESLISIKTANPASQGVNLKSGKSITAWIEY
KNEECRLRVFLSNSSLKPSKALLDVGIDLSSYLKEVMFVGFSGSTEGSTELHLIENWTFNTS
GFVSARPRFNPHNVSDSSVIVSPNISLSDSGNGRHSRLGLGLGIAGPAFFCAVIAVFGFFSLM
KWRRIRTQKSIKAELLTGPREFSYKELKTATKGFHSSRIIGNGAFGTVYKAFCISSGNISAVK
RSKHSHEGKTEFLAELSIIARLRHKNLVQLQGWCVEKGELLLVYDFMPNGSLDKLLYQES
SEASLLNWSHRYNIAVGLASVLTYLHQECEQQVIHRDIKTGNVLLDGNFNARLGDFGLAK
LMDHDKSPVSTLTAGTMGYLAPEYLQYGKATEKTDVFSYGVVILEVACGRRPIEREPGTQ
KMVNLVDWVWGLHSQGKIIEAADSRLNGEFKEDEMKKLLLVGLSCANPDSSTRPSMRKV
LQILNNEAEPALVPKMKPSLTFSCGFSLTVDDIIIEEEGGGEWETSRPTIVQID
> gene:Csa3G734040
MANFCFFSFLAIFFFTVPAASHNFFYAGFRDPTAATNLTLPDIAKIEENGLLTLTDDGGFQQG
HVFYESPVRFRNSSNADPFSFSTNFVFAIVNEFPNLGGHGLAFTIAPSKNLHALPVQFLGLL
NSTNHGDPSNHLFAVEFDTFKNAEFEDIDDNHIGIDLNSLISSASTTASYFINDGNTKQFINL
KSGAPIQVWIDYDAPVNSLTVALSPFSTKPQKPILSFNVDLSPILFEFMYIGFSASTGQMSSS
HYILGWSFSTDGPSQSLNIDSLPSVPGPKNTYSDLAIGISILIILIVIAGIFLTLYKIKKKIDIIEA
WELEIGPHRYPYKQLELATKQFSNRELLGRGGFGKVYRGTLPNSKTRVAVKRISHESKQGL
REFMSEITIIGRLRHRNLVQLLGWCRHGGNEDLLLVYEFMVNGSLDSYIFGKPKVILSWEQ
RFKIINGVASGLLYLHEGYEQVVIHRDVKASNVLLDDEMNGKLSDFGLAKLYEHGENPTT
TRVVGTVGYLAPELHRTGKATTSSDVYAFGALVLEVACGRRPIGPREVPEEIVLVDWVWE
KYKEKKLLEVMDEKLKGDFNEVEAVMILKLGLLCSKDSAAARPSMRLVMRCLDGEIGVP
DEITGPRMVEGADEFVDSWSDNRDITSASLSTSSLSILDGRS
> gene:Csa7G048050
MSSSSSSSSFLFCAVISILLFISTSVFATEFVFNTNFTSTNTLLFGNATIDSSVLILTRDSPFTIGR
ALYPFKVPIHFSNSLFSFASSFIFSVAPQPNLFPGHGFAFLFTPFTGINGTSSAQNLGLFNFTN
NGSPSNHVFAVEFDSFQNLEFNDTNDNHVGVDLNSLESNASFAAGFWSGPDDGEFKELKI
NNGETYQVWIECLDSLVNITMAEVGMKRPRKPLISLFVDFSGLLLDEMYVGFTAATGQLV
QSHRILSWSFSTSNLSIGDALLITDLPSFVPQKEGTIFNSRAFILGITFGGVGLVIICFIICGVVI
IKRRGRKKPKDDEIEDWELEYWPHRFAYEDVYEATGGFSEANVIGSGRNGKVYKGTLGR
SKVAVKRISVEAESGMREFVAEISSLGRLKHRNLVKLIGWCKKEKGSLILMYDYMENGSL
DKKLFECNENERLSWEKRMKILKDVATGLLYLHQGWDSRVLHRDIKGNNVLLDKDMNA

RLGDFGLARMQPHEKTADTTRVMGTVGYMAPEVVRTGRVSAQADVFGFGVLVLEVVCG
RRAVEEGKPWLIDWVKGLMERNEIGLAVDERLRVEVISGNEIDEMERMVCLGLLCAHNE
AGARPTMQQVVNILCERNGSGSNDGLLNRLRSTRILSEISQGKNFQQNHPTFEEIKTSSSST
SFIESDILKNDR
> gene:Csa_7G067410
MAAQNNLLIHLFFLVPFATSLSFNFTSFNQGNADMIYDRTFPTNQVIELTGDSSNNNMNFV
GRATYSQPLHLWDEGSGNMSSFQTHFSFAINSRGRANYGDGLTFFFAPNGSILQANISRGS
GLGIGYDPELWNGTATFFAVEFDIYSNNFDPPFEHVGIDINSMKSIAYSRWKCDIMGGKRN
DVWINYDSDTHNLSVVFSGFENNNTLLQHLHHVVDLRLNLPEWVTFGFSASTGYEYATH
SVYSWSFHSTLELTLEPTFTTDPNSVASAPSPGPSLPPNNNDGSTSKTGLEIGLGIAGGVIFV
GGLVIVWIIVWKKMAAMKNIEEEIMLDDSEFEKGKGPRRFLYKELARATNNFKEDKKLGE
GGFGGVYKGFLRELNCNVAVKRISKGSKQGIKEYASEVKIISQLRHRNLVQLIGWCHEKDE
LLLVYEFMPNGSLDTHLFKPNNFLTWELRYKIGQGIASALLYLHEEWEMCVLHRDIKSSN
VMLDLNYNAKLGDFGLARLVNHGKGSQTTALAGTLGYLAPECATTGRATKETDVYSFGI
VALEIACGRMPFNPNVEEEKMVMVEWVWKLYGCGKVLDAIDSKLRKEIRSFGDEEKMM
ECLMVVGLWCAHPDSNARPSIRQAINVLNFEAPLPILPSHLPAPTYDFRPIASSSTSSSTTQS
GVASLASNSSNL
> gene:Csa_1G004050
MEPRRRRRPPLLLLLLQLCIFFLFFFSSYAVDFFFNSFSNESNGTDFILISDARFDSPVIRLNN
DSNQNSIGRVFYHTKLTMKPTSNFSKISSFSTSFVFSILLEDATSPGFGLAFVLSNSTSPPGVI
AGQYFGLFTNSTTHVVAPLLAVEFDTGRNTEFNDPDDSHIGIDLNSVLSSKIHGAGYFGSN
GQFIPIQMRNGQNLRAWIEFDGANFEINVTIAPIGIPRPSIPTLSFRDPLIANYVSTEMFVGFS
ASKTKWVEAQRILAWSFSDTGVARDINTTNFPVFMRESPPSPLSPGSVAGIVIGCVLFVIIVS
GFGFFWYWRKNRAKEEDEEIEDWELEYWPHRFSNEELSQATDKFSEENLLGSGGFGRVF
KGTLPNHTEIAVKCVNHDSKQGLKEFMAEISSMGRLQHKNLVQMRGWCRKKNELMLVY
DYMPNGSLNRWIFDKPTTLLSWKQRRRVLGDVAEGLNYLHHGWDQVVIHRDIKSSNVLL
DSEMRGRVGDFGLAKLYQHGETPNTTRIVGTLGYLAPEIATVATPTAASDVYSFGVVVLE
VVCGRRPIELAAEEEEMVLIDWVRDLYSAGRLIAAADSRIREEYETEEIELMLKLGLACCH
PNPERRPTMREVVAVLIGEQPSAESVELLSGLAGGMVDDTRNVDI
> gene:Csa_3G734030
MARLCLLCFFFFLFLAAPAASQQLYFSGFQDDAAVAANLTLTDIAKIEQNGILKLTNDTSRL
QGHAFYSSPVRFKNSSDGKVFSFSTAFVIAVVPEYPTLGGHGLAFTIAPSKNLRGLPSQYLG
LLNAKDVGNFTNHLFAVEFDTVQDFEFADINDNHVGINLNHMISNVSTTASYFVDDGPTK
QNLTLKSGRPIQAWVDYDSSVNSLTVALSPSSTKPKKPILSFNVDLSPILDEFMYVGFSAST
GLLASSHYVLGWSFSMNGQARSLDLSSLPSVPGPKKKHTAFTIGISVAAVLIVIIAICVAVLII
WKIKNADIIEAWEHEIGPHRYSYKELKKATKRFRDKELLGRGGFGKVYKGTLPNSKIQVA
VKRISHESKQGLREFVSEIASIGRLRHRNLVQLLGWCRRRGDLLLVYDFMANGSLDNYIFD
DPDVNLSWQQRFGIIKGVASGLLYLHEGYEQVVIHRDVKASNVLLDSEMNGKLGDFGLA
RLYEHGANPSTTRVVGTLGYLAPELPRTGKATTSSDVYAFGALLLEVACGRRPIDPKSSSEE
LVLVDWVWENYREGKLLDVMDPKLKGDFNVVEAMMILKLGLFCSNDSAAARPSMRQV
VRFLEGEMGVPEEISAPKVMEGGRNGEGFDDFVNSFASSSFNKFSSYSSTGNKDMDMSFA
SFSTSPLSLLNGRD
> gene:Csa_2G439210
MANFLYVFSMLSHFILFLNLPLSVISVSFKIDQFKSDDNTILYQGDAVVLGGEILLSDPEFSC

HVGRAIYKDPIQIWDSETAKLTDFTTHFTFTIDTQKVPDYGQGFVFFLAPSGFQIPPNSAGG
FLGLYNKTYSNSVTNQIVHVEFDTGSNGWDPPYAHVGININSVTSSNDTRWNVSLHSGDL
AEVWISYNSTIKLLSVSWKYQKTSTLLENTTLSYPIDLTTVLPQQATVGFSAATGAHLERH
SVSSWEFNSTLDMKPTSISAGNKVSVIVGVTVSVGGLILVGIIVFVTLSRLKEKKRKKDQE
NLEEVNLTSINDDLERGAGPRRFSHKLLAMATNNFSNERKLGEGGFGAVYRGYIQDLDLN
IAVKKISRGSRQGRKEYITEVKIISRLRHRNLVQLIGWCHDKGEFLLVYEFMSNGSLDSHLF
GKRTPLAWSVRYKIALGLGSALLYLHEEGEQCVVHRDIKSSNIMLDSNFNVKLGDFGLAR
LMDHELGAQTTGLVGTLGYLAPEYINTGRASKESDVFSFGVVALEIATGRVSRTSMEKESH
KGLVEWVWDLYGSGQLLEGVDAKLQSNFDKKQVECLMVVGLWSAYPDPNFRPSIKQVI
QVLNFEAAVPNLPNKMPVPTYNAPSTSMSSNEPSFTVSLDMGR
