# Supplementary figures and images for: Genome-Wide Identification and Characterization of Lectin Receptor-Like Kinase Gene Family in Cucumber and Expression Profiling Analysis under Different Treatments
Source: Genes (Basel). 2020 Sep 2;11(9):1032. doi: 10.3390/genes11091032 (PMC7564967; doi:10.3390/genes11091032)

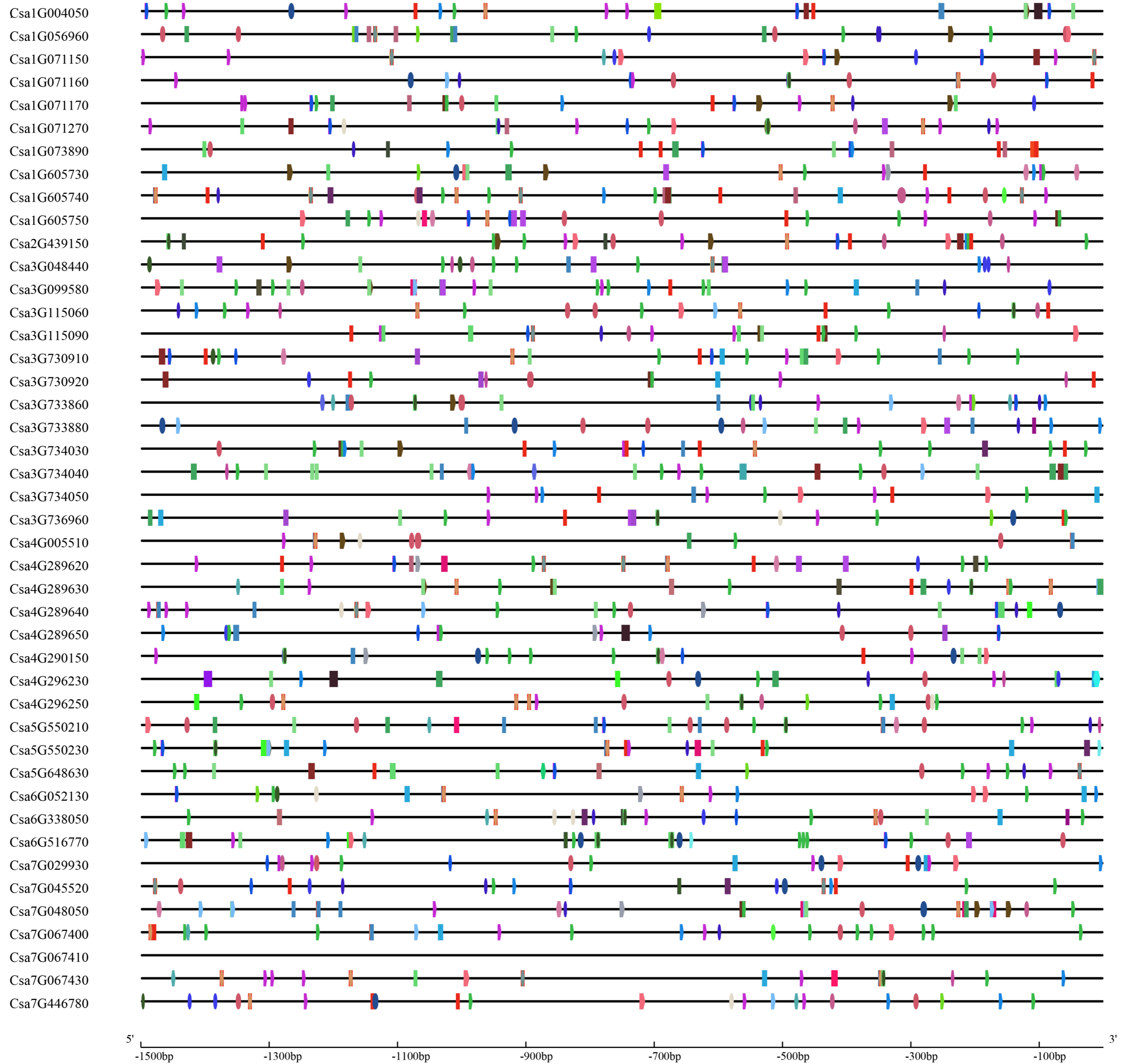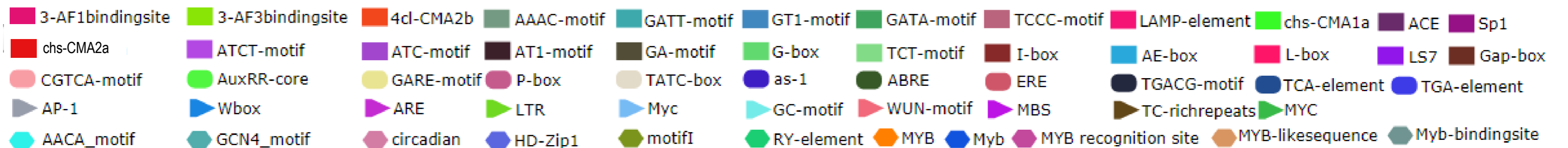

Supplement: Supplementary file 1 [file genes-11-01032-s001.zip › genes-891962-supplementary/Additional file2 Figure S2.pdf]
